# Near-infrared fluorescence imaging-guided surgery using cRGD-ZW800 to improve surgical resection margins in oral cancer: a phase I/II feasibility trial

**DOI:** 10.1038/s41467-026-73554-7

**Published:** 2026-05-22

**Authors:** B. E. Zweedijk, L. J. Lauwerends, H. A. Galema, D. J. Robinson, H. S. de Bruijn, H. Abbasi, T. L. March, A. R. P. M. Valentijn, M. Pool, H. Mast, B. P. Jonker, J. A. U. Hardillo, D. Monserez, A. Sewnaik, S. Koljenovic, C. Verhoef, R. J. Baatenburg de Jong, J. V. Frangioni, S. A. Koppes, D. E. Hilling, A. L. Vahrmeijer, S. Keereweer

**Affiliations:** 1 Department of Otorhinolaryngology, Head and Neck Surgery, Erasmus MC Cancer Institute, University Medical Center Rotterdam, Rotterdam, The Netherlands; 2 Department of Surgical Oncology and Gastrointestinal Surgery, Erasmus MC Cancer Institute, University Medical Center Rotterdam, Rotterdam, The Netherlands; 3 Department of Imaging Physics, Delft, University of Technology, Delft, The Netherlands; 4 Department of Surgery, Leiden University Medical Center, Leiden, The Netherlands; 5 Department of Clinical Pharmacy and Toxicology, Leiden University Medical Center, Albinusdreef 2, Leiden, The Netherlands; 6 Department of Oral and Maxillofacial Surgery, Erasmus MC Cancer Institute, University Medical Center Rotterdam, Rotterdam, The Netherlands; 7 Department of Pathology, Antwerp University Hospital/ University of Antwerp, Antwerp, Belgium; 8 Curadel Surgical Innovations, 11 Erie Drive, Natick, MA USA; 9 Department of Pathology, Erasmus Medical Center, University Medical Center Rotterdam, Rotterdam, The Netherlands

**Keywords:** Surgical oncology, Phase II trials, Oral cancer detection, Fluorescence imaging

## Abstract

In oral squamous cell carcinoma (OSCC) surgery, inadequate tumor margins are reported in up to 85% of cases, adversely affecting outcomes. In this prospective, single-center study (n = 31; NCT04191460), we evaluated the safety and imaging feasibility of cRGD-ZW800-1, a near-infrared fluorescent integrin-targeted tracer. The secondary objective was to determine whether intraoperative fluorescence imaging could identify inadequate resection margins and inform surgical decision-making. Patients received 0.01, 0.025, or 0.05 mg/kg cRGD-ZW800-1, and tracer uptake was quantified using in vivo multi-diameter single-fiber reflectance and single-fiber fluorescence spectroscopy to optimize dosing and timing. All doses were well tolerated, achieving tumor-to-background ratios exceeding 4.5, with optimal performance at 0.025 mg/kg. Fluorescence imaging detected all 23 inadequate margins, including nine undetected by conventional assessment (sensitivity 100% versus 70%), altered surgical plans in five cases, and avoided adjuvant radiotherapy in three cases. These findings demonstrate that cRGD-ZW800-1 is safe, tumor-specific, and facilitates intraoperative margin assessment in OSCC.

## Abstract (web summary)

Incomplete tumor removal during oral cancer surgery remains a major clinical challenge. Here, the authors show, in a feasibility trial, that fluorescence imaging using the integrin-targeted tracer cRGD-ZW800 is safe, achieved a patient-level sensitivity of 100%, enabled additional fluorescence-guided resections and prevented postoperative radiotherapy in some patients with oral squamous cell carcinoma.

## Introduction

Achieving complete tumor removal in oral cancer surgery remains a significant clinical challenge. Although preoperative imaging improves surgical planning, intraoperative margin assessment still relies on the surgeon’s visual and tactile judgment. As distinguishing tumor from healthy tissue can be very difficult, inadequate margins, defined as tumor-positive ( < 1 mm) or close ( ≤ 5 mm), are reported in 67–85% of cases, with up to 40% being tumor-positive^1–4^. These margins are strongly associated with local recurrence, the need for adjuvant therapy, and poor survival outcomes^2,5–9^. Selective frozen section analysis is currently the gold standard to assess surgical margins intraoperatively. However, this technique is time-consuming, can only examine a small part of the specimen, and is subject to sampling errors, thus frequently resulting in a high false-negative rate^10^. Therefore, there is a clear need for more accurate, objective, real-time, intraoperative techniques for tumor margin identification.

Fluorescence imaging (FI), using tumor-specific near-infrared (NIR) fluorescent agents, can be of additional value to guide the surgeon in the complete resection of tumors^11^. Several clinical studies have recently shown promising results regarding targeting specificity, imaging contrast, and intraoperative usability^11–19^. While FI has achieved high detection rates for tumor-positive margins in oral cancer (up to 100%), sensitivity for close margins remains limited (43–67%)^19^. As previously mentioned, close margins significantly increase recurrence and lower survival^8,9^, making the exploration of alternative fluorescent agents to mitigate close margins imperative. Additionally, NIR FI offers significant advantages over label-free techniques (e.g., autofluorescence), including enhanced tissue penetration and minimized interference from blood, making it particularly effective for imaging deep tumor tissue. These advantages have been confirmed in recent studies, underscoring the potential of NIR FI to improve the accuracy of tumor detection during surgery^20,21^.

cRGD-ZW800-1 is a zwitterionic, integrin-targeted fluorescent agent consisting of a cyclic pentapeptide (cRGD) conjugated to an 800 nm fluorophore ZW800-1^22,23^. Because of their low non-specific uptake in normal tissue, zwitterionic targeting ligands tend to exhibit higher tumor-to-background ratios (TBRs). cRGD, a clinically well-known peptide, binds to a wide range of integrins that are associated with neoangiogenesis. The αvβ6 integrin is also well-suited as a target for tumor imaging, as it is highly overexpressed in most invasive epithelial cancer types, including oral cancer, whereas its expression in normal tissue is limited^24,25^. cRGD-ZW800-1 has shown tumor-specific imaging without toxicity in healthy participants and colon cancer patients^14^. Integrin expression levels are variable among different tumor types and different anatomical sites involve distinct tissue optical properties (e.g., scattering and light absorption) that affect the fluorescent signal in unpredictable ways. Therefore, a feasibility study in oral cancer is required, including correction of the fluorescent signal intensity to tissue optical properties^10,16^.

Multi-diameter single-fiber reflectance and single-fiber fluorescence (MDSFR/SFF) spectroscopy corrects for tissue optical properties to quantify intrinsic fluorescence, enabling objective comparison between tumor and healthy tissue. While previously used in clinical fluorescence imaging trials^15,24,25^, true in vivo quantification of intrinsic fluorescence over time has never been achieved, primarily due to technical limitations. In this study, we applied MDSFR/SFF spectroscopy to measure cRGD-ZW800-1 concentrations in tumor and healthy tissue at multiple time points, providing valuable pharmacodynamic data. The oral cavity’s accessibility allowed direct contact measurements, offering unique insights into the tracer’s temporal behavior in vivo.

The aim of this study is to evaluate the safety and feasibility of cRGD-ZW800-1 in patients with oral squamous cell carcinoma (OSCC), and to identify the optimal timing interval and dose for intraoperative detection and margin assessment. Building on these findings, we further examine the potential of FI with cRGD-ZW800-1 in detecting inadequate resection margins and assess its impact on surgical decision-making.

## Results

Thirty-one patients with biopsy-proven OSCC scheduled for standard-of-care surgery were included in this study. The patients consisted of eight women and twenty-three men, with an average age at inclusion of 66 years (range, 34–91). Patient and study characteristics are summarized in Table 1.

**Table 1:** Study characteristics and inadequate resection margin detection using FI with cRGD-ZW800-1

Pt	Dose(mg/kg)	Tumor site	T- stage^a^	Intraoperative margin status	Inadequate margin found with IOA?	Inadequate margin found with FI?	Extra resection performed?	Postoperative margin status
1	0.05	Lateral tongue	cT3	Close	Yes	Yes	Yes	Clear
2	0.05	Lateral tongue	cT1	Close	Yes	Yes	No	Close
3	0.05	Posterior tongue	cT2	Positive	Yes	Yes	Yes	Close
4	0.05	Retromolar trigone	cT2	Close	No	Yes	Yes	Close
5	0.05	Lateral tongue	cT2	Close	Yes	Yes	Yes	Clear
7	0.05	Lateral tongue	cT2	Close	Yes	Yes	No	Close
8	0.05	Lateral tongue	cT4a	Positive	Yes	Yes	Yes	Close
6	0.01	Floor of mouth	cT2	Clear	n/a	n/a	n/a	Clear
9	0.01	Lateral tongue	cT2	Close	No	Yes	Yes	Close
10	0.01	Floor of mouth	cT1	Close	-**	Yes	No	Close
11	0.025	Alveolar process mandibula	cT2	Clear	n/a	n/a	n/a	Clear
12	0.025	Retromolar trigone	cT2	Positive	Yes	Yes	Yes	Close
13	0.025	Alveolar process mandibula	cT2	Close	No	Yes	No	Close
14	0.025	Alveolar process maxilla	cT4b	Clear	n/a	n/a	n/a	Clear
15	0.025	Floor of mouth	cT1	Close*	Yes	Yes	Yes	Clear
16	0.025	Lateral tongue	cT2	Close*	Yes	Yes	Yes	Close
17	0.025	Floor of mouth	cT1	Positive	No	Yes	Yes	Close
18	0.025	Alveolar process	cT2	Close	Yes	Yes	Yes	Clear
19	0.025	Alveolar process	cT4	Positive	Yes	Yes	Yes	Close
20	0.025	Lateral tongue	cT2	Positive	Yes***	Yes	Yes	Clear
21	0.025	Lateral tongue	cT3	Clear	n/a	n/a	n/a	Clear
22	0.025	Ventral tongue/floor of mouth	cT2	Clear	n/a	n/a	n/a	Clear
23	0.025	Lateral tongue	cT2	Close	Yes	Yes	Yes	Close
24	0.025	Lateral tongue	cT2	Clear	n/a	n/a	n/a	Clear
25	0.025	Lateral tongue	cT3	Close	No	Yes	No	Close
26	0.025	Lateral tongue	cT3	Positive	No	Yes	Yes	Clear
27	0.025	Buccal mucosa	cT4a	Clear	n/a	n/a	n/a	Clear
28	0.025	Lateral tongue	cT2	Clear	n/a	n/a	n/a	Clear
29	0.025	Floor of mouth	cT2	Positive	No	Yes	No	Positive
30	0.025	Floor of mouth	cT3	Close	No	Yes	No	Close
31	0.025	Lateral tongue	cT1	Close	No	Yes	Yes	Clear

^a^ TNM staging was based on American Joint Committee on Cancer (AJCC). Cancer Staging Manual, 8th edition (2017). Springer.

* Second primary lesion present.

** No IOA performed.

*** Partially, of the multiple inadequate margins only one was detected using IOA.

*IOA* intraoperative assessment, *FI* fluorescence imaging, *n/a* not applicable.

### Safety and tolerability

No signs of acute or chronic toxicity were observed following administration of cRGD-ZW800-1, and no hypersensitivity reactions occurred. Across the study cohort, twelve adverse events (AEs) and five serious adverse events (SAEs) were reported in eight patients (Supplementary Table 1). All reported AEs and SAEs were unrelated to cRGD-ZW800-1 and were attributed to the expected postoperative course. Importantly, no AEs occurred between tracer administration and surgery. Furthermore, the use of FI did not contribute to further morbidity. Additional resections were performed only when histopathologically justified and involved only a few millimeters of extra tissue, which did not necessitate unplanned reconstructions and therefore did not prolong hospital stay.

Vital signs and ECGs remained stable following tracer injection, with no clinically significant changes observed. A decrease in hemoglobin (Hb) levels was noted in three patients, likely resulting from pre-existing malnutrition and dehydration frequently associated with oral cavity cancer due to pain and impaired oral intake. Given that all patients received intravenous fluids prior to tracer administration, the Hb decrease is attributed to hemodilution rather than any tracer-related effect, consistent with prior reports^26,27^. All administered doses (0.01, 0.025, and 0.05 mg/kg) were well tolerated.

### In vivo quantification of cRGD-ZW800-1

**Fig. 1:**
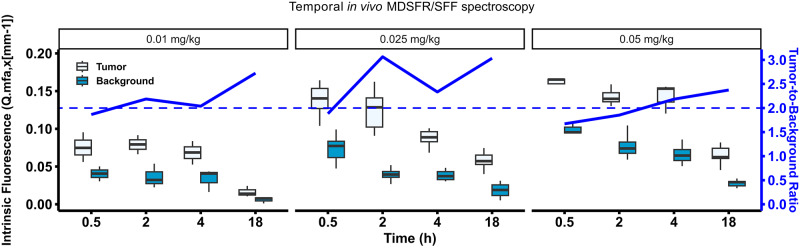
Temporal quantification of intrinsic fluorescence with MDSFR/SFF spectroscopy at 0.5, 2, 4 and 18 h post-administration for dose groups 0.01 (*n* = 3), 0.025 (*n* = 21) and 0.05 mg/kg (*n* = 7) (left to right), where “n” denotes the number of patients allocated to each dose group. Measurements were performed in triplicate in the center of the tumor and on the contralateral side of the oral cavity. This step consisted of direct contact fiber probe measurements at three time points after administration (0.5, 2, 4 and 18 h). Q.μ^f^_a,x_ was significantly higher in the tumor compared to healthy tissue for all time points in dose groups 0.025 and 0.05 mg/kg (*p* < 0.001). Blue lines represent the mean in vivo tumor-to-background ratios (weighted quotient of the tumor and background region Q.μ^f^_a,x_), that increase over time in dose groups 0.025 and 0.05 mg/kg. The horizontal dashed line represents the TBR threshold of 2. Box plots show the median (center line), the interquartile range (box bounds; 25–75th percentiles), and whiskers extending to the most extreme data points within 1.5× the interquartile range from the quartiles. *MDSFR/SFF* multi-diameter single-fiber reflectance and single-fiber fluorescence. The source data is available as a Source Data File.

Temporal quantification of the intrinsic fluorescence (Q.μ^f^_a,x_) values with MDSFR/SFF spectroscopy revealed TBRs of 2.72 ± 2.27 (*n* = 3), 3.03 ± 1.36 (*n* = 21) and 2.37 ± 0.59 (*n* = 7) for dose cohorts 0.01, 0.025 and 0.05 mg/kg, respectively, measured 18 hours post-administration. Although TBR (i.e., imaging contrast) after 18 hours was relatively high for the group that received 0.01 mg/kg, the Q.μ^f^_a,x_ of tumor tissue was very low (0.02 ± 0.01), and not significantly greater than the intrinsic background fluorescence (*p* = 0.114). Importantly, Fig. 1 demonstrates sufficient contrast (TBR ≥ 2.0) after only two hours for the group that received 0.025 mg/kg, suggesting that administration on the day of surgery is feasible, making it logistically more favorable.

### Dose finding and injection time window

The first dose group (*n* = 7) received 0.05 mg/kg cRGD-ZW800-1. Excellent intraoperative contrast and tumor-specificity were found in all cases (Fig. 1). These results allowed for dose de-escalation for the second dose cohort, which was set to receive 0.01 mg/kg. After inclusion of three patients, interim analysis clearly showed adequate TBRs, further increasing over time (Fig. 1). However, absolute in vivo fluorescence intensity was consistently relatively low, indicating less optimal intraoperative usability. It was therefore decided to include a third dose cohort (*n* = 7) that received an intermediate dose of 0.025 mg/kg.

### In vivo FI before resection

**Fig. 2:**
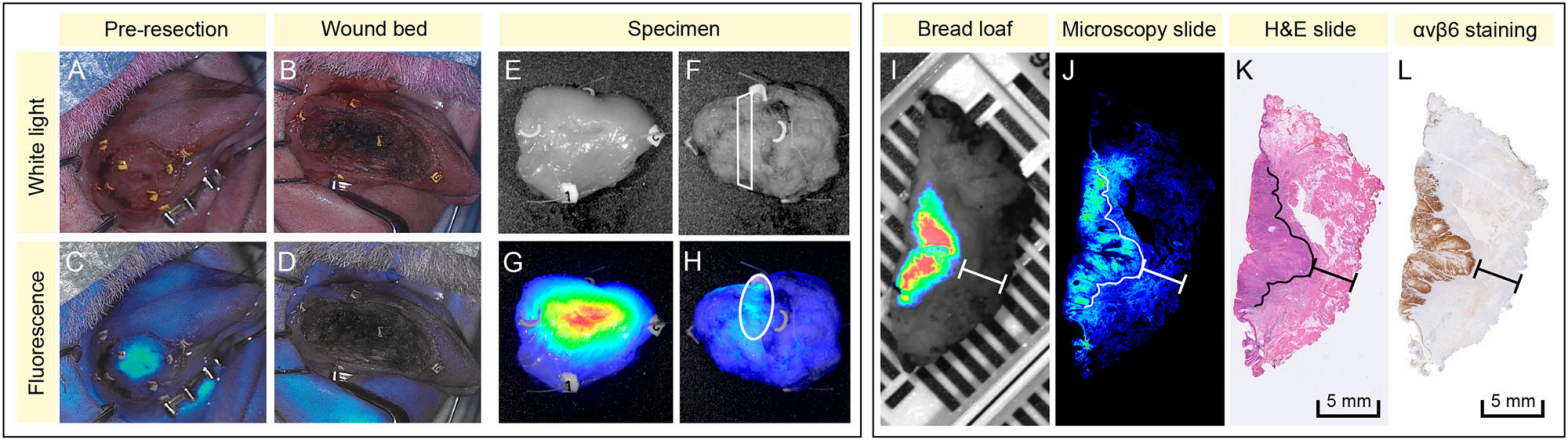
Intra- and postoperative fluorescence imaging of an oral squamous cell carcinoma of the lateral tongue from a patient with a close surgical margin. In vivo fluorescence imaging of the outlined tumor before resection (**A**, **C**), and of the wound bed, after resection (**B**, **D**), by ex vivo fluorescence imaging of the resected specimen in a closed-field system (**E–H**). The specimen is imaged from different angles, e.g., mucosal (**E**, **G**) and the deep margin (**F**, **H**), where a fluorescence signal is visible in the close margin (solid oval). Finally, fluorescence in formalin-fixed bread loaves (**I**, outlined in **F**) and deparaffinized microscopy slide (**J**) are correlated with H&E histopathology (**K**) and αvβ6 immunohistochemistry (**L**). In (**I**, **J**, **K**, **L**) the tumor is delineated, and close margin indicated in white/black. Images (**A–D**) were obtained with the Quest Spectrum Platform, (**E–I**) were obtained with the Pearl Trilogy, **J** was obtained with the Odyssey CLx Imaging System, **K** and **L** were digitally scanned with a virtual microscope (Nanozoomer 2.0 HT, Hamamatsu Photonics, Shizuoka, Japan), and the scale bar was determined using NDP.view2 Viewing software (Hamamatsu Photonics, Shizuoka, Japan). *H&E: Hematoxylin and Eosin*.

Intraoperative FI did not interfere with the standard procedure, and the total incremental operation time related to in vivo FI never exceeded 10 min (mean 5.52 minutes, range: 4–10 min). Strong mucosal fluorescence was observed in all tumors before resection in all patients (Fig. 2C). Referencing the initial mucosal outline defined by the surgeon prior to imaging, the fluorescent areas never suggested inadequate margins, supporting the surgeon’s accurate assessment, subsequently confirmed by histopathology. This indicates that mucosal delineation can be achieved with well-defined boundaries and high contrast using FI, resulting in adequate identification of all mucosal margins. Supplementary Movie 1 presents an example of a clearly fluorescent tumor obtained intraoperatively, aiding the surgeon in its identification and delineation during the procedure. Although a consistent fluorescence signal was observed in healthy gingiva (Supplementary Fig. 1), ex vivo FI revealed sufficient mucosal contrast with a mean TBR of 2.04 (SD 0.79) in gingival tumor specimens. Furthermore, in vivo spectroscopy measurements confirmed that all individual patients with gingival tumors achieved adequate TBRs, with the lowest measured TBR being 2.1, indicating that physiological fluorescence in healthy gingival did not impose an issue for mucosal tumor delineation. In one patient, a second primary lesion with a diameter of 5 mm was found by the surgeon during inspection, also showing a clear fluorescent signal.

### In vivo FI of the wound bed

Residual fluorescence in the wound bed was observed in 4 of 31 cases (13%). In one patient with a tumor located in the alveolar process, the surgeon suspected residual malignancy immediately post-resection. Intraoperative FI corroborated this suspicion by revealing a localized fluorescent signal in the wound bed. Following pathological confirmation by IOA, a limited additional resection was performed at the corresponding site. In the remaining three cases, fluorescence was detected in non-suspicious anatomical structures: the mandibular bone marrow at the resection plane (*n* = 2) and lingual salivary gland tissue (*n* = 1). None of these areas were deemed clinically suspicious, and the corresponding margins on the tumor specimen were histologically tumor-negative.

### Intraoperative ex vivo closed-field imaging of the specimen

Consistent with pre-resection FI, a well-demarcated fluorescent signal was seen in the tumor on the mucosal side of the freshly resected specimen in all imaged (*n* = 31) cases. Intraoperative closed-field resection specimen FI could not be performed for one case, due to a malfunction of the imaging device. Fluorescent areas were also observed on the deep surface (i.e., the non-mucosal margin of the specimen which is exposed after resection) of all imaged specimens. The vast majority of these visible fluorescent spots were integrin-specific, indicating the presence of either salivary glands (*n* = 14) or inadequate margins (*n* = 23). While fluorescent, salivary gland tissue was macroscopically easily distinguishable by the surgeon and pathologist, posing no clinically relevant challenges. In the two cases of partial mandibulectomy mentioned above, the corresponding surgical cut on the specimen showed fluorescent signal too, but there was no inadequate bony margin.

The regions of highest fluorescence on the deep margin were identified and correlated with final histopathology (i.e., the sentinel margin approach^28,29^). All close (1-5 mm) and positive ( < 1 mm) resection margins exhibited fluorescence intensity-peaks (example shown in Fig. 2H). In the full cohort (n = 31), 15 close and 8 positive margins were all successfully detected using FI, whereas nine inadequate margins were missed by standard IOA by surgeon and pathologist (three positive and six close margins). Resulting in a detection rate of 100% for FI (23/23), compared with 61% for IOA (14/23). The case in which closed-field inspection could not be performed due to malfunction of the device had a close margin (1–5 mm, *n* = 1), which exhibited a clear fluorescence signal during postoperative FI. Focusing on the optimal-dose cohort (*n* = 21), all 14 inadequate margins (six positive and eight close margins) were again detected by FI, while IOA missed seven cases (three positive, four close). This corresponded to a detection rate of 100% for FI (14/14), compared with 50% for IOA (7/14). Consequently, FI demonstrated a significant higher inadequate margin detection rate compared to standard IOA for both the full cohort (100% vs. 61%, Exact binomial McNemar’s test, *p* = 0.004) and the optimal-dose cohort (100% vs. 50%, Exact binomial McNemar’s test, *p* = 0.008).

### Diagnostic performance of FI

To assess diagnostic accuracy, all surgical resection planes were included, together with any separate tumor lesions identified within the specimen. In total, 155 surgical resection planes were assessed in 31 patients (5 resection planes for each patient). Of these resection planes, 56 exhibited fluorescence, yielding a total of 63 distinct fluorescent spots. In 47 (74.6%) of these spots, an inadequate margin was confirmed on definitive histopathological assessment. The remaining 16 (25.4%) fluorescent spots corresponded to histopathologically adequate resection margins and originated from non-tumor tissue, specifically salivary gland tissue (*n* = 14) or bone marrow (*n* = 2). Ninety-nine resection planes were histopathologically adequate and showed no fluorescent signal. In addition, one second primary lesion (5 mm in diameter) demonstrated clear fluorescence both in vivo and ex vivo. In another case, a second primary lesion measuring 1.4 mm exhibited a distinct fluorescent signal ex vivo using closed-field flatbed imaging (Supplementary Fig. 2, top row), whereas no fluorescence was detected in vivo. Furthermore, one patient presented with multiple microsatellite lesions within the specimen, at a distance of >5 mm from the surgical margin, consisting of several tumor foci with diameters ranging from 50 to 300 µm (total area <0.8 mm), none of which showed fluorescence (Supplementary Fig. 2, bottom row). Overall, these findings resulted in a sensitivity of 98.0% (95% CI, 90.2–99.6%) and NPV of 99.0% (95% CI 94.5–99.9%) for FI. Additional diagnostic performance metrics included a specificity of 86.1% (95% CI 79.1–91.1%), a PPV of 75.4% (95% CI, 63.7–84.6%), and an overall accuracy of 89.7% (95% CI 84.9–94.5%). These results demonstrate robust performance of FI, even when including areas that would not have impacted surgical decision-making. The reduced specificity was largely attributable to fluorescence originating from salivary gland tissue (*n* = 14), which can be readily distinguished from tumor tissue during surgery.

For standard IOA, each surgical specimen was systematically evaluated across the same five tissue-tissue resection planes, yielding a total of 155 assessed planes. As noted above, definitive histopathology confirmed that 47 of these margins were inadequate. IOA correctly identified 34 of these inadequate planes (72.3%), while 13 (27.7%) were missed. The remaining 108 planes were histopathologically adequate and were all classified as adequate during IOA. Notably, standard IOA detected the previously described larger second primary tumor (5.0 mm in diameter) but failed to identify both the microsatellite lesions and the small second primary tumor (1.4 mm in diameter). Consequently, IOA demonstrated a sensitivity of 70.0% (95% CI 57.2–80.5%), while maintaining a specificity of 100% (95% CI, 96.6–100%). This performance reflects a substantially higher false-negative rate compared with FI, highlighting that, despite similar overall accuracy, IOA misses a significantly greater number of tumor foci (Exact binomial McNemar’s test, *p* < 0.001). Importantly, minimizing false negatives is clinically more critical than minimizing false positives, as undetected tumor tissue poses a direct risk to patient outcomes^2,5–9^, whereas false-positive findings primarily result in additional tissue sampling without compromising patient safety. Further details on the diagnostic performance of both IOA and FI are provided in Table 2.

**Table 2:** Diagnostic performance of FI with cRGD-ZW800-1 compared to standard-of-care IOA across all histopathological findings, using definitive histopathological assessment as reference standard

	FI	IOA
True positive (TP)	49^a^	35 ^d^
False positive (FP)	16^b^	0
False negative (FN)	1^c^	15^e^
True negative (TN)	99	108
Sensitivity (95% CI)	98.0% (49/50) (90.2-99.6%)	70.0% (35/50) (57.2-80.5%)
Specificity (95% CI)	86.1% (99/115) (79.1-91.1%)	100% (108/108) (96.6-100%)
Accuracy (95% CI)	89.7% (148/165) (84.9-94.5%)	90.5% (143/158) (85.5-94.1%)
Positive predictive value (PPV) (95% CI)	75.4% (49/65) (63.7-84.6%)	100% (35/35) (90.0-100%)
Negative predictive value (NPV) (95% CI)	99.0% (99/100) (94.5-99.9%)	87.8% (108/123) (81.4-92.2%)

FI: fluorescence imaging; IOA: intraoperative assessment; CI: confidence interval.

^a^ true positive fluorescent signal originating from inadequate (i.e. positive ( < 1 mm) and close (1–5 mm)) resection margins (*n* = 47), and second primary tumors (*n* = 2).

^b^ false positive fluorescent signal originating from bone (marrow) (*n* = 2) and salivary gland (n = 14).

^c^ false negative finding caused by a small tumor focus (total area <0.8 mm) consisting of multiple microsatellite lesions with diameters ranging from 50 to 300 µm (*n* = 1).

^d^ true positive referring to inadequate (i.e. positive ( < 1 mm) and close (1-5 mm)) resection margins (n = 34) and second primary tumors (*n* = 1) correctly identified by IOA.

^e^ false negative findings caused by inadequate (i.e. positive ( < 1 mm) and close (1–5 mm)) resection margins (n = 13), second primary (*n* = 1), and the aforementioned small tumor focus containing multiple microsatellite lesions (*n* = 1).

95% CIs were calculated using the ANOVA-type Wilson score.

### Clinical performance of FI

When evaluating the potential clinical impact of the FI performance, we found that all inadequate margins were consistently detected, yielding a patient-level sensitivity of 100% (95% CI 85.7–100%) and a NPV of 100% (95% CI 61.6–100%). In addition, the specificity was 75.0% (95% CI 40.9–93.6%), the PPV was 92.0% (95% CI 75.2–98.3%), and an overall accuracy was 93.5% (95% CI 80.5-98.5%). The observed specificity of 75.0% was entirely attributable to two instances of a false-positive fluorescent signal in bone marrow, which were clinically non-suspicious but fell within the resection plane of the primary tumor and were therefore included in the analysis. In the subgroup analysis, which assessed the detection of both positive and of close margins separately, FI achieved a sensitivity of 100% and specificity of 75.0% in both analyses. When compared with standard IOA, overall accuracy for FI and IOA was broadly similar; however, patient-level sensitivity was markedly higher for FI compared to IOA (100% vs. 70.0%, respectively), reflecting that, in our study population, FI prevented false-negative findings altogether. Further details on the clinical evaluation of FI are provided in Table 3.

**Table 3:** Patient-level evaluation of FI with cRGD-ZW800-1 for the detection of inadequate surgical margins, using definitive histopathological assessment as reference standard

	FI	FI	FI
	*Close* and *positive (<1-5 mm)* vs. *clear* margins	*Close (1-5 mm)* vs. *clear* margins	*Positive (<1 mm)* vs. *clear* margins
True positive (TP)	23^a^	15^c^	8 ^d^
False positive (FP)	2^b^	2^b^	2^b^
False negative (FN)	0	0	0
True negative (TN)	6	6	6
Sensitivity (95% CI)	100% (23/23) (85.7-100%)	100% (15/15) (81.7-100%)	100% (8/8) (67.7-100%)
Specificity (95% CI)	75.0% (6/8) (40.9-93.6%)	75.0% (6/8) (40.9-93.6%)	75.0% (6/8) (40.9-93.6%)
Accuracy (95% CI)	93.5% (29/31) (80.5-98.5%)	91.3% (21/23) (74.5-97.8%)	87.5% (14/16) (62.7-97.4%)
Positive predictive value (PPV) (95% CI)	92.0% (23/25) (75.2-98.3%)	88.2% (15/17) (66.8-96.9%)	80.0% (8/10) (51.8-94.6%)
Negative predictive value (NPV) (95% CI)	100% (6/6) (61.6-100%)	100% (6/6) (61.6-100%)	100% (6/6) (61.7-100%)

FI: fluorescence imaging; CI: confidence interval.

^a^ true positive fluorescent signal originating from inadequate (i.e. positive ( < 1 mm) and close (1–5 mm)) resection margins (*n* = 23).

^b^ false positive fluorescent signal originating from bone (marrow) at the resection margin (*n* = 2).

^c^ true positive fluorescent signal originating from close resection margins (*n* = 15).

^d^ true positive fluorescent signal originating from positive resection margins (*n* = 8).

95% CIs were calculated using the ANOVA-type Wilson score.

### Effect of FI on intraoperative decision making

As all dose cohorts showed a detectable fluorescence signal using both in vivo and ex vivo FI, all patients (*n* = 31) were included in margin assessment. As previously reported, FI combined with standard IOA revealed inadequate margins in 23 patients (74%, 23/31), including eight patients with positive margins and 15 with close margins (Table 1). Additional resections were performed in 70% of the patients with inadequate margins (16/23), comprising seven positive margins and nine close margins. Notably, 31% (5/16) of these resections were based exclusively on FI findings, which had been overlooked during the initial IOA. In contrast, no further resections were undertaken in 30% (7/23) of patients with inadequate margins, which included one with a positive margin and six with close margins. In one patient with a close margin, an additional resection was not feasible due to tumor proximity to critical anatomical structures. Furthermore, in one patient no IOA was performed; in this case, a pre-emptive additional resection had already been carried out during surgery and was deemed sufficient at the time. However, definitive pathology later revealed a close margin elsewhere, which, in retrospect, was apparent on FI. Importantly, in four patients, the tissue samples for assessment were mistakenly taken from areas immediately adjacent to, rather than directly from, the fluorescent spots identified by FI. This sampling error resulted in seemingly clear margins during standard IOA. To ensure a thorough and accurate evaluation, these cases were carefully reviewed in collaboration with the dedicated head and neck pathologist. Each corresponding histological section was examined individually, and the spatial relationship between the fluorescent signal and the inadequate margin was precisely mapped. This detailed review confirmed that the fluorescence signal had accurately indicated the location of the inadequate margin in all four cases. Accordingly, these cases were classified as true positives with respect to FI. These cases highlight an important limitation of relying solely on frozen section analysis. If fluorescence findings had been acted upon without the mandatory frozen section confirmation required by the study protocol, the inadequate margins would have been identified and addressed intraoperatively. Lastly, in one patient, additional resection was not performed because achieving clear margins would have required a marginal mandibular resection, and the periosteum was clinically intact. In accordance with our standard practice, the surgeon deliberately opted against further resection.

Fluorescent signals detected on the deep margins of the specimens, areas not deemed clinically suspicious during IOA, prompted further examination with frozen sections. This identified nine inadequate margins that had been missed by IOA (three positive, six close). In two positive and three close margins, this resulted in additional resections. Final histopathological analysis confirmed that these extra resections were free of malignant tissue, thereby ensuring that all five patients ultimately achieved wider surgical margins. Notably, in three of these cases, the FI-guided additional resections allowed for the avoidance of adjuvant radiotherapy. In the remaining four cases, no additional resections were performed due to wrong tissue sampling (*n* = 3) and tumor proximity to critical structures (*n* = 1), as previously explained.

With a p-value of less than 0.001, the null hypothesis, that the adequate margin rate is 15% or less, was strongly rejected. This provides robust statistical evidence that the proportion of adequate surgical margins following the implementation of FI is significantly higher. The pre-specified A’Hern single-stage Phase II design was based solely on the optimal dose group (*n* = 21), calculated under the assumption that only the optimal dose group would be eligible for analysis, given the initial uncertainty regarding fluorescent signal in other dose cohorts. Within the optimal dose group, 14 patients had in inadequate surgical margin, of which only 7 (50%) were identified using IOA. In contrast, FI identified all inadequate margins (100%). As previously mentioned, additional resections could not be performed in all cases due to various reasons. Had these additional resections been feasible, the percentage of adequate margins following the implementation of this technique would have been even higher, further strengthening the statistical significance of the findings.

### Postoperative closed-field imaging of bread loaves

**Fig. 3:**
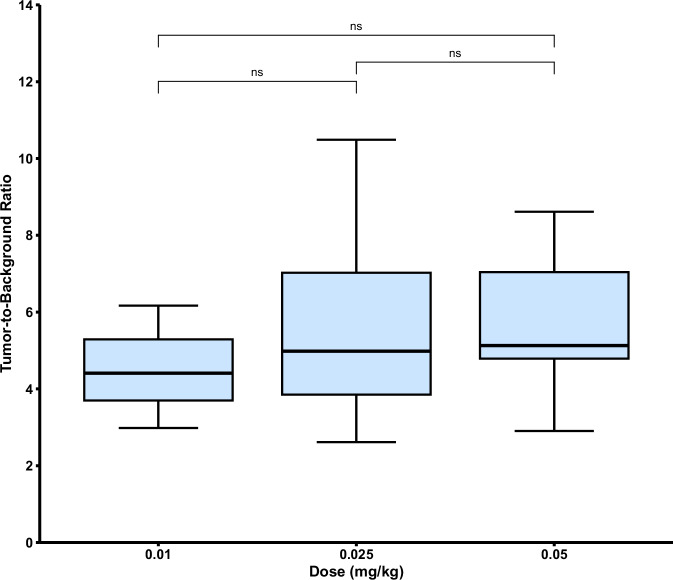
Ex vivo Tumor-to-Background Ratios (TBR) in bread loaves, for doses 0.01 mg/kg (*n* = 3), 0.025 mg/kg (*n* = 21) and 0.05 mg/kg (*n* = 7), where “n” denotes the number of patients allocated to each dose group. For all dose groups TBRs were adequate. Box plots show the median (center line), the interquartile range (box bounds; 25th–75th percentiles), and whiskers extending to the most extreme data points within 1.5× the interquartile range from the quartiles. The source data is available as a Source Data File. ns: non-significant (two-sided Mann-Whitney U-test).

Absolute fluorescence intensities increased for both tumor and background tissue with increasing dose (Supplementary Fig. 3*)*. The Mean Fluorescence Intensity (MFI) was significantly higher in tumor than in background tissue for all doses (*p* < 0.05). The mean ex vivo TBRs were adequate for all cases at 4.52 ± 1.60 (*n* = 3), 5.73 ± 2.75 (*n* = 21) and 5.76 ± 2.03 (*n* = 7) for dose cohorts 0.01, 0.025 and 0.05 mg/kg, respectively (Fig. 3).

### Correlation between final histopathology and FI

Final histopathological analysis revealed 15 cases with close margins and 1 case with a positive margin. This specific positive margin was detected using FI, but no additional resection could be performed as the frozen section was taken adjacent to the fluorescent area. Had we relied solely on FI, even this single positive margin could have been addressed, resulting in zero positive margins postoperatively. Without the use of FI, final histopathology would have revealed three positive margins, as two positive margins were missed during standard IOA. All inadequate margins were located at the deep resection plane, and all were successfully visualized intraoperatively using FI. IHC staining showed a high correlation between tumor localization, αvβ6 expression and fluorescence signal (Fig. 2, image J-L), emphasizing the specificity of cRGD-ZW800-1. Some mild fluorescence was also observed outside the tumor boundaries (Fig. 2, image J), originating from a desmoplastic reaction at the tumor border. This region exhibited αvβ6 expression, although at substantially lower levels compared to tumor. Importantly, this low-level signal was not detectable during imaging of the fresh specimen or bread loaves and did not interfere with intraoperative margin assessment.

Additionally, the previously mentioned second primary tumor that demonstrated fluorescence both in vivo and ex vivo, was confirmed to be malignant. The second primary tumor that only exhibited a fluorescent signal ex vivo showed colocalized αvβ6 expression during IHC staining (Supplementary Fig. 2, top row). Furthermore, despite the absence of detectable fluorescence both in vivo and ex vivo on the patient with multiple micro-satellite lesions, IHC staining revealed elevated αvβ6 expression (Supplementary Fig. 2, bottom row).

### cRGD-ZW800-1 uptake in non-tumor tissues

Fluorescent gingival tissue exhibited αvβ6 expression at the junctional epithelium (Supplementary Fig. 1). Salivary gland tissue was, regardless of presence of inflammation, consistently strongly fluorescent, with IHC staining revealing αvβ6 expression in the ductal cells (Supplementary Fig. 4). Ex vivo images revealed increased fluorescence in bone (marrow). Here, IHC staining showed no αvβ6 expression (Supplementary Fig. 5).

## Discussion

This study demonstrated that cRGD-ZW800-1 was safe, well-tolerated, tumor-specific, and effectively detected inadequate margins during FI of oral cancer. Most importantly, FI altered surgical decision-making in five cases (16%, 5/31) by identifying inadequate margins that were overlooked during routine IOA, underscoring the clinical value of intraoperative FI. Tumors fluoresced intensely both in vivo and ex vivo, and visible fluorescence (excluding salivary glands) in the deep margin was in all cases congruent with margin inadequacy. Study proceedings did not meaningfully interfere with the standard of care, nor resulted in significantly prolonged surgery time. Given the use of FI in this study (pre-resection, wound bed and on the resected specimen), this is a type E study when considering the effect of FI on intraoperative decision-making^10^.

To comprehensively evaluate the performance of FI, we conducted two complementary analyses within this study. First, a diagnostic accuracy analysis evaluated all histopathological findings, both tumor-positive and tumor-negative, in relation to fluorescence, irrespective of their impact on margin status. Second, a patient-level analysis focused exclusively on clinically relevant margins to explore the potential implications of fluorescence for intraoperative decision-making. While specificity was higher in the diagnostic accuracy analysis compared with the patient-level analysis (86.1 vs. 75.0%, respectively), sensitivity remained consistently high in both analyses (98.0% vs. 100%, respectively). The lower specificity in the patient-level analysis resulted from the more stringent definition of true negatives, as only patients with completely adequate margins across all five surgically assessed resection planes were classified as true negatives, whereas any inadequacy in a single plane excluded the case from the true negative count. Within the study population, only one tumor focus was not detected (microsatellite lesions, total diameter <0.8 mm); however, this focus had no clinical impact on surgical management, as it was located at the specimen at a distance of >5 mm from the resection plane and therefore did not affect margin status. In comparison, IOA demonstrated substantially lower sensitivity (70%, 95% CI 57.2-80.5%). When comparing the diagnostic accuracy and patient-level analyses, the patient-level approach is particularly informative for surgical practice, as it captures the added value of FI in detecting clinically relevant inadequate margins and guiding intraoperative decisions. The consistently high patient-level sensitivity of FI is clinically significant, since missing inadequate margins is closely linked to local recurrence, the need for adjuvant therapy, and poorer survival outcomes^2,5–9^. Despite comparable overall accuracy between FI and IOA (93.5% vs. 90.5%, respectively), FI demonstrated markedly higher patient-level sensitivity (100% vs. 70.0%), underscoring its ability to substantially reduce false negatives and enhance detection of inadequate margins.

Building on these findings, it is noteworthy that, in addition to the high patient-level sensitivity for detecting all clinically relevant inadequate margins, FI also achieved 100% sensitivity in detecting both close and positive margins separately.These findingsunderscoretheabilityof FIto reliably detect clinically relevant inadequate margins thatmight otherwise have been missed.Although IOA is regarded as the gold standard for intraoperative margin assessment at our institution, it isresource-intensive and routinely available only in selected centers^2,7^. Consequently, the present comparison may underestimate the true added value of FI, as in typical clinical practice its benefit would likely be even greater, reflected by the high rate of close or positive margins after initial resection prior to IOA (23/31, 74%).Collectively,our findingshighlight a distinct performance profile compared to earlier reports. For instance, de Wit et al.^19^ reported a patient-level sensitivity of 100% for tumor-positive margins but only 67.4% for close margins using EGFR-targeted FI with cetuximab-800CW, with a combined specificity of 41.7%. Furthermore, van Keulen et al.^30^ evaluated fluorescently labeled panitumumab-IRDye800 in oral cancer, achieving 95% sensitivity and 89% specificity for detecting inadequate margins ( < 5 mm). Compared with these studies, our approach achieved a sensitivity of 100% and a specificity of 75%, reflecting robust detection of both positive and close margins while maintaining acceptable specificity.

The observed variability in specificity, both within the present study and relative to previously reported values, is partly attributable to the inherent challenges of defining and enumerating true negative findings in fluorescence-guided surgery. In this study, we defined true negatives as non-fluorescent surgical resection planes histopathologically confirmed as adequate, providing a practical yet approximate framework. Alternative specimen partitioning or negative area counting could alter these metrics, underscoring their limitations. Consistent with prior literature, this emphasizes interpreting FI by its clinical impact and intraoperative guidance value, rather than formal diagnostic accuracy reliant on an ill-defined denominator^10^. Still, our results show it reliably identified all clinically relevant inadequate margins, achieving 100% sensitivity and 75.0% specificity in the patient-level analysis, thereby accurately highlighting critical margins for surgical decision-making.

In this study, intraoperative FI was substantiated through objective in vivo assessment of tracer pharmacokinetics using MDSFR/SFF spectroscopy. By correcting for patient-specific optical tissue properties, known to be influenced by a wide range of often unquantifiable biological and anatomical factors^31^, this method enabled accurate assessment of tracer kinetics in vivo and guided optimization of dose and timing. This study uniquely combines qualitative FI with quantitative spectroscopy, offering a robust framework for future clinical translation of targeted fluorescent tracers.

Although the 0.01 mg/kg dose group showed relatively high TBRs, the absolute tumor fluorescence was too dim for reliable intraoperative visualization. This underscores a critical distinction: TBR is a post-processed metric that does not always correlate with the subjective visual contrast perceived during surgery. Both the 0.025 and 0.05 mg/kg groups demonstrated sufficient intensity and TBRs >2 starting 2 h post-injection, and remaining consistently above this threshold for at least 18 h in the 0.025 mg/kg cohort. Therefore, 0.025 mg/kg was identified as the “near-optimal” dose (per regulatory definitions) since it achieved equivalent imaging performance to the 0.05 mg/kg dose with half the amount of tracer. As a zwitterionic small molecule, cRGD-ZW800-1 demonstrated high TBRs and favorable pharmacokinetics, characterized by rapid tumor accumulation and efficient clearance from non-target tissues, advantages over conventional, antibody-based fluorescent tracers^22,32,33^.

Importantly, adequate TBRs were achieved as early as 2 h post-injection, highlighting a practical window of 2–18 h for imaging. This flexibility enables same-day administration, offering a logistical advantage over full-length antibody-based tracers such as panitumumab-IRDye800CW and cetuximab-IRDye800CW, which require administration several days prior to surgery, often necessitating additional hospital visits and pre-dosing with unlabeled antibody to improve contrast^15,16,19,29,34^. Furthermore, infusion reactions have been reported with cetuximab, even in the unlabeled form^16,35,36^. No adverse events were associated with cRGD-ZW800-1 in this study. In addition, cRGD-ZW800-1 targets integrin αvβ6, a biomarker overexpressed in OSCC and concentrated at invasive tumor fronts, with limited expression in normal epithelium^37,38^. While the FDA-approved fluorophore indocyanine green (ICG) has been widely studied for intraoperative detection of oral cancers. Its non-specific accumulation in surrounding tissues often leads to a decrease in tumor contrast, limiting tumor delineation. In contrast, cRGD-ZW800-1 provides receptor-mediated tumor targeting and lower background fluorescence, resulting in higher tumor-to-background ratios under intraoperative conditions^20,39^. This target specificity enables precise discrimination at the deep margin, resulting in a 100% detection rate of inadequate surgical margins (Table 1).

In all subjects and dose groups, fluorescence was observed in uninvolved gingiva, consistent with αvβ6 expression in healthy junctional epithelium^40,41^. As none of the cases presented with an inadequate mucosal margin, this fluorescent signal did not impose any clinically relevant difficulties. In general, the deep margin is rightly considered more important than the mucosal margin, as 87% of inadequate margins involve the deep margin, and no significant difference in recurrence rate was found between close and clear mucosal margins^42^. As mentioned before, salivary gland tissue also showed an αvβ6-specific fluorescence signal, which could be accounted for with relative ease, as salivary gland tissue was easily distinguishable by both surgeon and pathologist. Prior studies on oral cancer imaging using cetuximab-800CW observed similar fluorescent signals originating from salivary gland tissue (29-47% of false positives)^16,19^. Lastly, a false-positive fluorescence signal was observed in the mandibular bone marrow. Importantly, all of the aforementioned false-positive signals, including those detected in uninvolved gingiva and salivary gland tissue, were clinically not suspicious for malignancy. Each fluorescent area underwent careful histopathological evaluation, thereby confirming the absence of malignant tissue. As a result, these signals did not influence intraoperative decision-making, lead to unnecessary resections, or compromise patient safety. This observation underscores that, although such false-positive signals may occur, their presence can be effectively managed through standard pathological confirmation without impacting the clinical utility of fluorescence-guided surgery.

In one case, multiple micro-satellite lesions (diameters varying from 50 to 300 µm) did not exhibit fluorescence in vivo or ex vivo. While IHC demonstrated expression of the integrin (Supplementary Fig. 2, Image F), tumor fields were sparse and widely spaced, spanning a maximum diameter of <0.8 mm. The lack of fluorescence is likely due to the fact that, although integrin expression indicates early neovascularization processes, functional blood vessels had not yet formed or connected to these lesions. As a result, the fluorescent tracer was unable to reach the tumor areas, leading to the absence of fluorescence signal. In another case, a second primary tumor (maximum diameter of 1.4 mm) was not fluorescent during in vivo FI, but was detectable during ex vivo FI (Supplementary Fig. 2, top row). Although such small secondary lesions are uncommon, these findings highlight a potential limitation for identifying subclinical tumor deposits using the current intraoperative camera systems. In this case, the missed second primary tumor had no impact on margin status, as they were located within the original planned resection area.

Within head and neck pathology, the definition of an inadequate surgical margin remains a topic of debate. Literature suggests that thresholds for close or positive margins may vary depending on tumor subsite, biological characteristics, and factors such as the worst pattern of invasion (WPOI)^43,44^. In this study, we applied a 5 mm cutoff in line with our institute’s protocol and consistent with the Royal College of Pathologists and National Comprehensive Cancer Network (NCCN) guidelines^45,46^. Importantly, the exact cutoff is less critical for the purpose of evaluating FI, as the primary goal was to assess whether this technique could reliably identify margins that are narrower than the defined threshold. While this approach allows for standardized assessment within our cohort, it is important to acknowledge that other institutions or clinical settings may adopt different margin definitions based on local practice. Nevertheless, despite these variations, our results demonstrate that fluorescence imaging has the potential to enhance intraoperative margin detection, independent of the specific numerical cutoff applied.

In four cases, accurately targeting fluorescent signals for intraoperative frozen section sampling proved challenging, limiting immediate resection. Importantly, this did not reflect failure of FI itself, as these cases were carefully reviewed in collaboration with the head and neck pathologist, confirming that the fluorescent signal accurately corresponded to the inadequate margins in all cases. These observations underscore the potential of FI to reliably identify inadequate margins, while also illustrating the technical learning curve in integrating FI into clinical practice and highlight the need for standardized protocols and training to ensure accurate localization and sampling. Implementation of FI in standard surgical practice could improve intraoperative decision-making, provided that its use is optimized through appropriate training.

cRGD-ZW800-1 demonstrated favorable safety profile and strong tumor specificity; however, long-term oncologic outcomes remain to be established. To robustly determine the clinical utility of FI in OSCC surgery, prospective trials are needed to assess its effect on long-term outcomes, including recurrence rate, survival and quality of life. A multicenter randomized controlled trial will be critical to determine whether FI significantly reduces the incidence of positive margins compared to standard surgical care. Defining this effect will be essential to confirm FI’s potential to improve oncologic outcomes and to support its broader integration into standard surgical practice.

Building on the encouraging results from this Phase I/II study, the next steps involve the development of a multicenter randomized controlled trial to provide data that could inform potential changes in national clinical guidelines. By demonstrating the clinical benefits of FI in real-world settings, it is hypothesized that FI can be established as a standard tool in oral cancer surgery, ultimately improving surgical precision and patient outcomes. Preparations for this multicentre trial are currently ongoing.

In conclusion, this study establishes cRGD-ZW800-1 as a safe, effective, and tumor-specific fluorescent tracer for oral cancer surgery. Uniquely, it combines qualitative intraoperative FI with objective, quantitative in vivo spectroscopy to validate tracer performance and inform optimal dosing and timing strategies. The integration of temporal pharmacokinetic insights, enabled by MDSFR/SFF spectroscopy, allowed for data-driven optimization of the imaging protocol.

Importantly, FI guided the identification of all inadequate resection margins, including those missed during routine intraoperative assessment, directly influencing surgical decision-making in real time and preventing adjuvant radiotherapy in selected cases. These findings highlight the substantial oncological value of fluorescence-guided surgery in improving margin assessment and surgical precision. Together, the clinical feasibility, safety profile, and diagnostic performance of cRGD-ZW800-1 support its further development and integration into surgical workflows aimed at improving outcomes in patients with oral cavity cancer.

## Methods

### Clinical trial design

This prospective single-center, single arm Phase I/II study was performed in 31 patients diagnosed with OSCC at the Erasmus Medical Center (Rotterdam, The Netherlands) from July 2022 until April 2025. The study protocol (available in the Supplementary Information file) was approved by the local Ethics Review Committee (METC Erasmus MC) and conducted in full compliance with the principles of the Declaration of Helsinki of 1975, the ICH GCP guidelines, and the laws and regulations of the Netherlands. As this study involved the use of an Investigational Medicinal Product (IMP), authorization was granted by the Central Committee on Research Involving Human Subjects (CCMO, the Netherlands), which serves as the national competent authority of clinical trials with IMPs. The study is registered in the European Clinical Trials Database (EudraCT 2019-003416-30) in December 2019, and ClinicalTrials.gov (NCT04191460) in December 2020.

Eligible patients were 18 years or older, and able to provide written informed consent. Patients with biopsy-proven active malignancies other than OSCC were excluded, as were those with a history of surgery, chemo- or radiotherapy involving the oral cavity. Pregnant or breastfeeding patients were also excluded, as were patients with significant clinically abnormal hematological status, kidney and liver function. To minimize selection bias, all eligible patients were consecutively recruited after screening at a multidisciplinary meeting, ensuring all were considered for inclusion.

The study initially consisted of two work packages (Work Package I and Work Package II). Work Package 1 aimed to determine the optimal dose and injection-to-imaging interval of cRGD-ZW800-1 through TBR and MFI values. Based on encouraging results from a prior investigation of cRGD-ZW800-1 in colorectal cancer^14^, three dose levels (0.01, 0.025, and 0.05 mg/kg) were evaluated. Secondary outcomes of Work Package I included safety and tolerability. Work Package II primarily aimed to assess whether intraoperative FI with cRGD-ZW800-1 could reliably detect all inadequate resection margins during oral cancer surgery. Secondary outcomes included changes in surgical management, correlation to IHC, and the influence of FI on operative time. For the purpose of this manuscript, both work packages have been combined into a single study. The overarching goal was to assess whether intraoperative FI with cRGD-ZW800-1 can reliably detect all inadequate resection margins during oral cancer surgery and influence intraoperative decision-making.

Per the standard of care, patients are admitted to the hospital the day before surgery. The agent was administered via a venous access line via a slow infusion for 5–10 min, approximately 18 h before surgery. Safety measurements entailed close patient monitoring before and after administration, consisting of repeated physical examinations, vital signs measurements, standard 12-lead-electrocardiograms (ECGs), and laboratory tests. Adverse events were monitored continuously by three independent researchers. When a complication was identified, it was classified according to the Common Terminology Criteria for Adverse Events (CTCAE), the required treatment was documented, and its progression or resolution was monitored.

After administration, MDSFR/SFF spectroscopy was performed in triplicate in the center of the tumor, on the edge of the tumor, and on normal tissue on the contralateral side of the oral cavity. This step consisted of direct contact fiber probe measurements at three time points after administration (0.5, 2 and 4 h) and once more before the start of surgery (18 h) to quantify the concentration of cRGD-ZW800-1 in and around the tumor over time.

### Investigational agent

The fluorescent contrast agent used in this study is cRGD-ZW800-1, a cyclic 5-amino acid sequence (cRGDyK) conjugated to the 800 nm NIR fluorophore ZW800-1, which binds specifically to integrins (αvβ1, αVβ3, αvβ5, αvβ6, αvβ8, α5β1, α8β1 and αIIbβ3)^22^. The drug substance and drug product were manufactured at the Interdivisional GMP Facility LUMC (IGFL), part of the department of Clinical Pharmacy and Toxicology, Leiden University Medical Center under Good Manufacturing Practice (GMP) conditions, in accordance with Directive 2001/20/EC.

### Surgical procedure and intraoperative fluorescence imaging

**Fig. 4:**
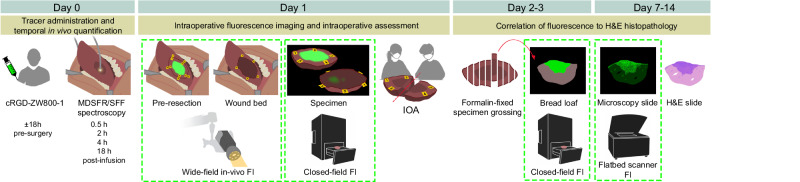
Clinical trial design. cRGD-ZW800-1 was administered intravenously approximately 18 h before surgery. This was followed by in vivo quantification of the tracer with MDSFR/SFF spectroscopy, consisting of probe point measurements on both tumor and healthy tissue (contralateral) at 4 different time points (including right before surgery). On the day of surgery, wide-field FI was performed in vivo before (i.e., after delineation and parallel tag placement) and after resection, followed by closed-field FI of the specimen from different angles. Routine IOA of the resection margins was performed by the surgeon and a pathologist. After grossing, closed-field FI of the formalin-fixed bread loaves was performed. Finally, deparaffinized 4µm-thick microscopy slides were imaged in a flatbed FI scanner. Fluorescence in the bread loaves and microscopy slides was correlated with H&E histopathology. *H&E: Hematoxylin and Eosin; MDSFR/SFF: multi-diameter single-fiber reflectance and single-fiber fluorescence; FI: fluorescence imaging; IOA: intraoperative assessment. Illustrations were created using Procreate, and the figure was assembled and annotated in Adobe Photoshop (version 26.8.1)*.

Surgeries were performed by six board-certified Head and Neck Surgeons, with surgical experience varying from 4 to 25 years. Open surgical-field intraoperative FI was performed using the CE-marked Quest Spectrum Platform (Quest Medical Imaging, Middenmeer, The Netherlands) by a team of experienced fluorescence imaging experts. This system utilizes excitation at 780 nm with a power density of 10 mW/cm², and emission is detected in the range of 800–900 nm. In our study, the gain was set to 22.5 (default), and we tested an exposure time of 38 ms (default). To standardize imaging conditions, we aimed to keep the camera distance between 20–30 cm and positioned it perpendicular to the sample, although slight variations occasionally occurred. During in vivo FI, the tumor was first delineated mucosally without the use of FI (Fig. 4), aiming for a 10 mm mucosal margin. FI was then performed, and any changes to the surgical plan based on fluorescence were documented. After resection, the wound bed was inspected for residual fluorescence, and the specimen was imaged ex vivo from multiple angles using the Pearl Trilogy Small Animal Imaging System (LI-COR Biosciences Inc., Nebraska, USA), a closed-field imaging system. This camera system uses excitation with 785 nm light at 3 mW/cm^2^ power density, and emission is captured in the range of 810-830 nm. The gain and exposure time (range of 500 ms to 60 s) are automatically set by the acquisition software (Image Studio^TM^) to produce a high dynamic range image. We adjusted the working distance to achieve the best focus, which varied depending on the specimen’s height. During ex vivo imaging, all surgical planes were systematically evaluated based on fluorescence images acquired with the closed-field imaging system and compared to definitive histopathological assessment.

After closed-field FI, specimen-driven intraoperative assessment (IOA) was performed. IOA is standard-of-care for intraoperative margin assessment in the Erasmus MC^47^: it is performed in close cooperation by the surgeon and pathologist, including gross and microscopic (fresh frozen sectioning) margin evaluation. If close or positive margins were identified, additional resections were guided by parallel tags placed during initial delineation^48^. To prevent bias, both surgeon and pathologist were blinded to ex vivo FI findings. Fluorescent signals at specimen margins were only disclosed after standard IOA was completed.

### Postoperative tissue analysis and immunohistochemistry

After IOA and any additional resections, all surgical specimens were mapped, formalin fixed and processed into bread loaves, which were then cut into blocks to be placed in cassettes. These sections were then analyzed separately using the Pearl imaging system to measure ex vivo fluorescence, before paraffin embedding.

Following ex vivo closed-field FI, definitive histopathological assessment was performed according to standard pathology protocols, evaluating all surgical resection planes, including both deep and mucosal margins. In line with these protocols, the resection specimen is oriented as a six-sided cuboid, where five surfaces represent true surgical resection margins (a standard approach also used in IOA), while the original mucosal surface (the exposed tumor-bearing side) does not constitute a tissue-tissue resection interface and is therefore excluded. Mucosal margins were assessed on the surgically created lateral planes, consistent with routine practice. We applied this same systematic evaluation to FI assessment, ensuring direct comparability with pathology. This aligns with a prior report on FI in oral cancer patients^19^. All tissue blocks were subsequently examined on hematoxylin and eosin (H&E)-stained sections by a dedicated head and neck pathologist, with definitive margin status determined independently of intraoperative findings.

After final histopathologic analysis, fluorescence imaging of the tissue slides was performed using a flatbed scanner (Odyssey M or Odyssey CLx, LI-COR Biosciences Inc., Nebraska, USA) to determine location of the fluorescent targeting agent, and to correlate fluorescent signal with H&E histopathology.

Finally, immunohistochemical (IHC) staining with the αvβ6 integrin was performed on representative 4 µm FFPE microsections containing fluorescent tissue, which included primary tumor, (occult) second primary tumor, healthy gingiva, healthy and inflamed salivary gland tissue, and atrophic bone (marrow) to verify αvβ6 expression. These microsections were also imaged using the Odyssey scanner.

### In vivo MSDFR/SFF spectroscopy

MDSFR spectroscopy determines the scattering and absorption coefficients (from the reflectance spectra), while SFF spectroscopy measures tissue fluorescence^16,49,50^. Quantitative intrinsic fluorescence (Q.μ^f^_a,x_) is defined as the product of the quantum efficiency across the emission spectrum Q[-], where Q is the fluorescence quantum yield of ZW800-1, and µ_a_^f^ [mm^-1^] is the tracer absorption coefficient at the excitation wavelength. The intrinsic fluorescence (Q.μ^f^_a,x_) of cRGD-ZW800-1 was calculated by correcting the raw fluorescence spectrum for the tissue optical properties. All measurements were repeated in triplicate and median values were calculated per measurement location. Details of the spectral correction, linear fitting procedure, and quantification limits are provided in Supplementary Fig. 6.

### Statistical analysis

For the MDSFR/SFF spectroscopy, 3 spectra were acquired consecutively at each measurement location. These were averaged (weighted by the confidence interval of the spectral fit to the data), to form a weighted average at each measurement location at each time point. The quotient of the median group means of the tumor and contralateral (healthy) tissue constituted the in vivo TBR.

Ex vivo TBR was calculated by drawing a region of interest (ROI) spanning the tumor, and another in adjacent (muscle) tissue using the Pearl’s integrated software (ImageStudio; LI-COR Biosciences Inc., Nebraska, USA). The MFIs were obtained per ROI. The (weighted) quotient of the tumor and muscle region MFI constituted the ex vivo TBR. Given that most inadequate margins occur in the deep margin rather than the mucosa^42^, ex vivo TBR was calculated on formalin-fixed bread loaves, as they contain the more relevant deep margin and adjacent muscle tissue. Similar to other studies, we observed that the fluorescence signal remained unaffected after formalin fixation^51^, ensuring the accuracy of our ex vivo TBR calculations.

In order to determine the sample size for dose-finding and feasibility, a lower threshold to differentiate signal from background noise is set at a mean TBR of μ0 = 2.0. Based on previous preclinical studies using cRGD-ZW800-1, and on experience from clinical trials using similar tumor-specific fluorescent targeting agents^52,53^, the μ1 is set to a mean of 4.0 with a standard deviation σ of 1.5. We have chosen a wider standard deviation than that observed in similar studies conducted on other cancer types, to account for the possibility of encountering a greater variation in integrin expression levels in oral cancers. Considering an α of 0.05 and a power of 80%, the sample size (n) is 7 per group, based on the One-Sample 2-sided t-test.

All patients with a detectable fluorescence signal were included in the surgical margin evaluation, which ultimately comprised the entire cohort (n = 31). Since signal detectability could not be guaranteed in advance, the required sample size was calculated solely for the optimal dose cohort. The primary endpoint is the rate of adequate surgical resection margins, based on the postoperative margin status. This analysis tests the null hypothesis that the adequate surgical margins rate is at most 15% versus the alternating hypothesis that the adequate surgical margins rate is at least 40%. The significance level (i.e., the probability of rejecting H0 when it is true) is α = 0.05 and the power (i.e., the probability of deciding the regimen is active) is 80%. Based on the A’Hern single stage Phase II design^54^, the expected sample size is 21. The minimal number of successes to indicate that the treatment is effective is 7.

The efficacy of FI was assessed by calculating standard diagnostic performance metrics, including sensitivity, specificity, negative predictive value (NPV), positive predictive value (PPV), and overall accuracy. Corresponding 95% confidence intervals (CI) were determined using an ANOVA-type Wilson score estimation that considers intra-patient clustering. To comprehensively evaluate both the intrinsic diagnostic performance and the clinical impact of FI, two complementary analyses were performed. First, a diagnostic accuracy analysis was conducted, in which all histopathological findings, both tumor-positive and tumor-negative, were evaluated in relation to fluorescence, irrespective of their impact on margin status. Second, a patient-level analysis was performed to evaluate findings relevant in the intraoperative surgical context. In this analysis, tissue clearly distant from the tumor and deemed non-suspicious by both surgeon and pathologist (e.g., en bloc resected salivary gland tissue) was excluded.

In the diagnostic accuracy analysis, all diagnostic performance metrics were derived by comparing both ex vivo intraoperative fluorescence findings and standard IOA with definitive (postoperative) histopathological assessment of the entire resection plane. Fluorescent areas corresponding to either tumor localization (including second primary lesions distant from the surgical cut) or a close margin were classified as true positives (TP), whereas non-fluorescent areas corresponding to healthy tissue (including clear margins) were classified as true negatives (TN). Furthermore, fluorescent areas corresponding to healthy tissue (including clear margins) were classified as false positives (FP), while non-fluorescent areas corresponding to either tumor localization (including second primary lesions distant from the surgical cut) or a close margin were classified as false negatives (FN). This analysis therefore included separate tumor-positive lesions within the specimen that were not located at the surgical margin and therefore would not have impacted surgical decision-making.

In the patient-level analysis, only margin-related findings that could potentially influence surgical management were included. This analysis focused exclusively on fluorescence findings at the surgically assessed resection planes and did not involve a combined evaluation with IOA. Fluorescent signals arising from areas clearly unrelated to the tumor margin, such as en bloc–resected salivary gland tissue outside the resection interface, were not considered relevant for intraoperative decision-making and were therefore excluded. Within this framework, fluorescent areas corresponding to inadequate (positive or close) margins were classified as TP, and a complete non-fluorescent margin surface corresponding to adequate margins classified as TN. Conversely, non-fluorescent areas corresponding to inadequate margins were considered FN, while fluorescent areas corresponding to clear margins were classified as FP. In addition, subgroup analyses were conducted, in which positive and close margins were evaluated separately. Specifically, one analysis compared positive margins with clear margins only, and one analysis compared close margins with clear margins only. In each case, fluorescence was regarded as a positive test result exclusively for the margin category under evaluation, and the same TP, TN, FP, and FN classification framework described above was applied.

Patient demographics, safety parameters, MFIs and TBRs were expressed in descriptive statistics (n, mean, SD). To assess differences in the inadequate margin detection between standard IOA and FI, the exact binomial McNemar’s test was used. Differences in MFI were analyzed using the Wilcoxon rank test, and differences in TBR were evaluated using the Mann-Whitney U test. A p-value of <0.05 was considered statistically significant. Adobe Photoshop (version 26.8.1) and R studio (version 4.3.2) were used for statistical analysis and graph design.

### Reporting summary

Further information on research design is available in the Nature Portfolio Reporting Summary linked to this article.

## Supplementary information

Supplementary Information

Description of Additional Supplementary Files

Supplementary Movie 1

Reporting Summary

Transparent Peer Review file

## Data Availability

All imaging, safety, clinical, and laboratory data (limited to non-identifiable information) can be obtained from the corresponding author upon request (S. Keereweer, s.keereweer@erasmusmc.nl). Data will be retained for at least 15 years in accordance with Dutch regulations. Additional data supporting the findings of this study are included in the Article, Supplementary Materials (including study protocol), or Source Data file. The Source Data File and the study protocol are publicly accessible via the following Figshare repository: 10.6084/m9.figshare.29763728. R code used for data analysis is publicly available via GitHub at: https://github.com/BoKosterZweedijk/R-code-Guided-by-Light-studie.git^55^.
