# Supplementary material for: Near-infrared fluorescence imaging-guided surgery using cRGD-ZW800 to improve surgical resection margins in oral cancer: a phase I/II feasibility trial
Source: Nat Commun. 2026 May 22;17:7341. doi: 10.1038/s41467-026-73554-7 (PMC13402695; doi:10.1038/s41467-026-73554-7)
Supplement: Supplementary file 1 — Supplementary Information [file 41467_2026_73554_MOESM1_ESM.pdf]

Improving surgical resection margins in oral cancer using real-time, integrin-specific near-infrared fluorescence imaging: a prospective, single-center study.

B.E. Zweedijk[1,2†] & L.J. Lauwerends[1†], H.A. Galema[1,2], D.J. Robinson[1], H.S. de Bruijn[1], H. Abbasi[1,3], T.L. March[4,5], A.R.P.M. Valentijn[5], M. Pool[5], H. Mast[6], B.P. Jonker[6], J.A.U. Hardillo[1], D. Monserez[1], A. Sewnaik[1], S. Koljenovic[7], C. Verhoef[2], R.J. Baatenburg de Jong[1], J.V. Frangioni[8], S.A. Koppes[9], D.E. Hilling[2,4], A.L. Vahrmeijer[4], S. Keereweer[1]*

[1] Department of Otorhinolaryngology, Head and Neck Surgery, Erasmus MC Cancer Institute, University Medical Center Rotterdam, The Netherlands
[2] Department of Surgical Oncology and Gastrointestinal Surgery, Erasmus MC Cancer Institute, University Medical Center Rotterdam, The Netherlands
[3] Department of Imaging Physics, Delft University of Technology, Delft, The Netherlands
[4] Department of Surgery, Leiden University Medical Center, Leiden, The Netherlands
[5] Department of Clinical Pharmacy and Toxicology, Leiden University Medical Center, Albinusdreef 2, 2333 ZA Leiden, The Netherlands
[6] Department of Oral and Maxillofacial Surgery, Erasmus MC Cancer Institute, University Medical Center Rotterdam, The Netherlands
[7] Department of Pathology, Antwerp University Hospital/ University of Antwerp, Antwerp, Belgium
[8] Curadel Surgical Innovations, 11 Erie Drive, Natick, MA 01760, United States
[9] Department of Pathology, Erasmus Medical Center, University Medical Center Rotterdam, The Netherlands

† Authors contributed equally to this manuscript

Stijn Keereweer MD, PhD
s.keereweer@erasmusmc.nl
Doctor Molewaterplein 40, 3015GD, Rotterdam, The Netherlands

Keywords

integrin; fluorescence imaging; fluorescence-guided surgery; image-guided surgery; molecular imaging; optical imaging; oral squamous cell carcinoma; oral cancer; head and neck cancer; tumor-specific; intraoperative imaging; targeted

Supplementary Table 1. Overview of Adverse Events

Dose cohort (mg/kg)	Patient ID	System organ class / preferred term	Symptom	Severity	Serious adverse event	Relationship to ZW800-1	Occurrence
0.05	1	Infections and infestations	Soft tissue infection: neck abscess	2	No	unrelated	Single occasion
0.05	3	Bleeding	Bleeding from free forearm flap at tongue base	3	Yes	unrelated	Single occasion
0.05	3	Respiratory, thoracic and mediastinal disorders	Pneumonia	2	No	unrelated	Single occasion
0.025	12	Infections and infestations	Wound infection	2	No	unrelated	Single occasion
0.025	13	Respiratory, thoracic and mediastinal disorders	Pneumonia	2	No	unrelated	Single occasion
0.025	13	Infections and infestations	Wound infection	3	No	unrelated	Single occasion
0.025	15	Infections and infestations	Infection of element 13- 21 (mouth)	3	No	unrelated	Single occasion
0.025	16	Infections and infestations	Wound infection	2	No	unrelated	Single occasion
0.025	19	Infection and infestations	Wound infection	2	No	unrelated	Single occasion
0.025	20	Other, specify	Trauma capitis	2	No	unrelated	Single occasion
0.025	23	Bleeding	Bleeding from free forearm flap	3	Yes	unrelated	Single occasion
0.025	23	Renal and urinary disorders	Prerenal kidney dysfunction*	2	Yes	unrelated	Single occasion
0.025	24	Bleeding	Bleeding from lateral tongue	1	Yes	unrelated	Single occasion
0.025	28	Bleeding	Bleeding from free forearm flap	2	No	unrelated	Single occasion
0.025	28	Renal and urinary disorders	Urinary tract infection	2	No	unrelated	Single occasion
0.025	28	Infections and infestations	Wound infection	2	No	unrelated	Single occasion
0.025	29	Bleeding	Bleeding from tongue	2	Yes	Unrelated	Single occasion

* The renal insufficiency was attributed to a prerenal cause due to fluid depletion and insufficient intake in this patient, with renal function recovering within 4 days following fluid resuscitation.

Supplementary Fig. 1. Fluorescence in the gingiva**.** The gingiva were consistently a source of *in vivo* fluorescence (A). Indeed, flatbed scanning fluorescence imaging confirmed localized fluorescent signal at the epithelial junction (D), which was confirmed with immunohistopathology to strongly express αvβ6 (E).

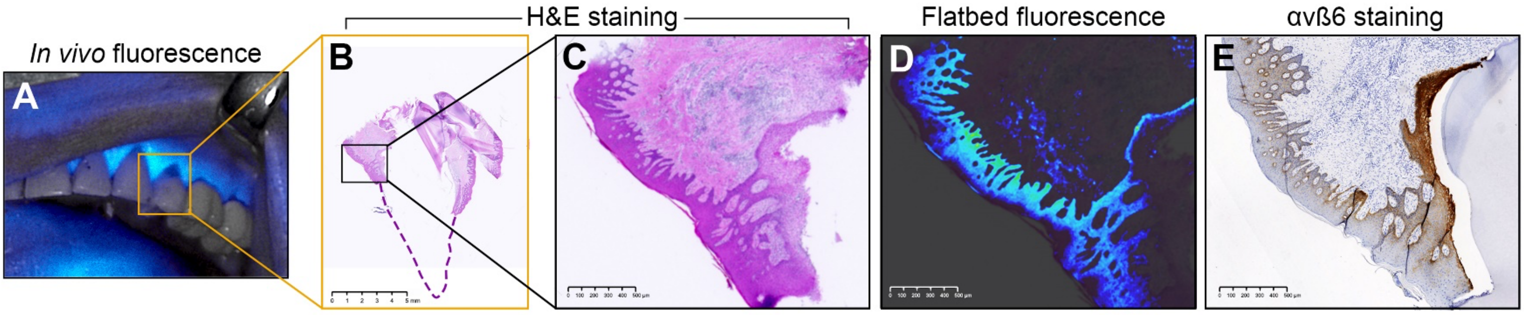

Supplementary Fig. 2. Additional lesions. Top row: H&E stained (A) second primary lesion which was not fluorescent *in vivo*, but was clearly fluorescent on flatbed scanning fluorescence images (B). Dense area of tumor fields exhibiting strong αvβ6 expression (C). Bottom row: H&E stained (D) multiple microscopic tumor fields (<0.4mm) which were not fluorescent *in* or *ex vivo* (E). Tumor fields exhibited strong αvβ6 expression (F), but due to their sparsity do not generate a detectable signal.

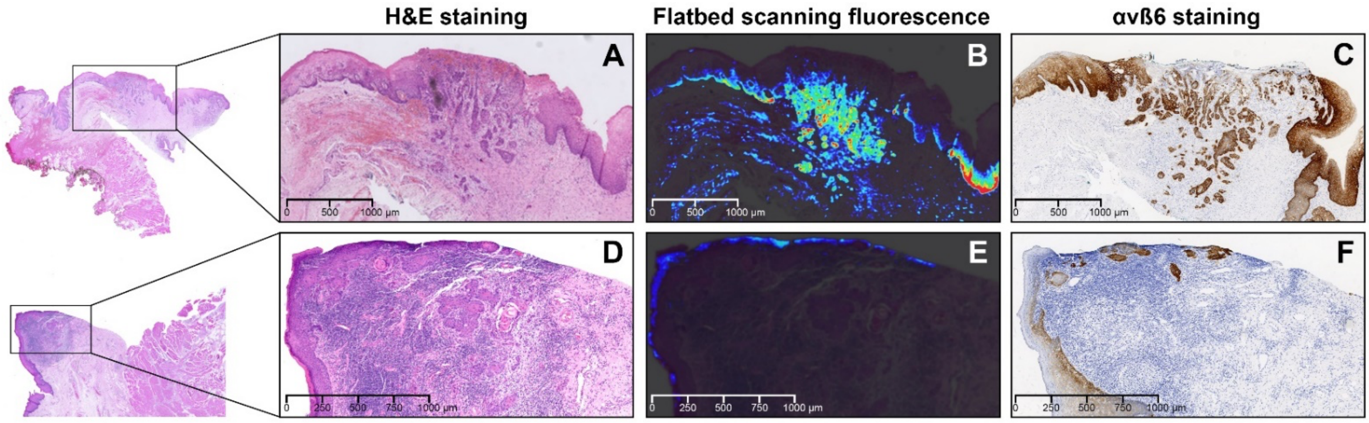

Supplementary Fig. 3. Mean fluorescence intensities (MFI) in bread loaves, for doses 0.01 mg/kg (n=3), 0.025 mg/kg (n=21) and 0.05 mg/kg (n=7), where "n" denotes the number of patients allocated to each dose group. For all dose groups, mean fluorescence intensities were significantly higher in tumor than in background tissue (p<0.05). Box plots show the median (centre line), the interquartile range (box bounds; 25th–75th percentiles), and whiskers extending to the most extreme data points within 1.5× the interquartile range from the quartiles, and dots indicate outliers. *The source data is available as a Source Data File. *: p < 0.005; **: two-sided p < 0.05 (Wilcoxon Rank-test).*

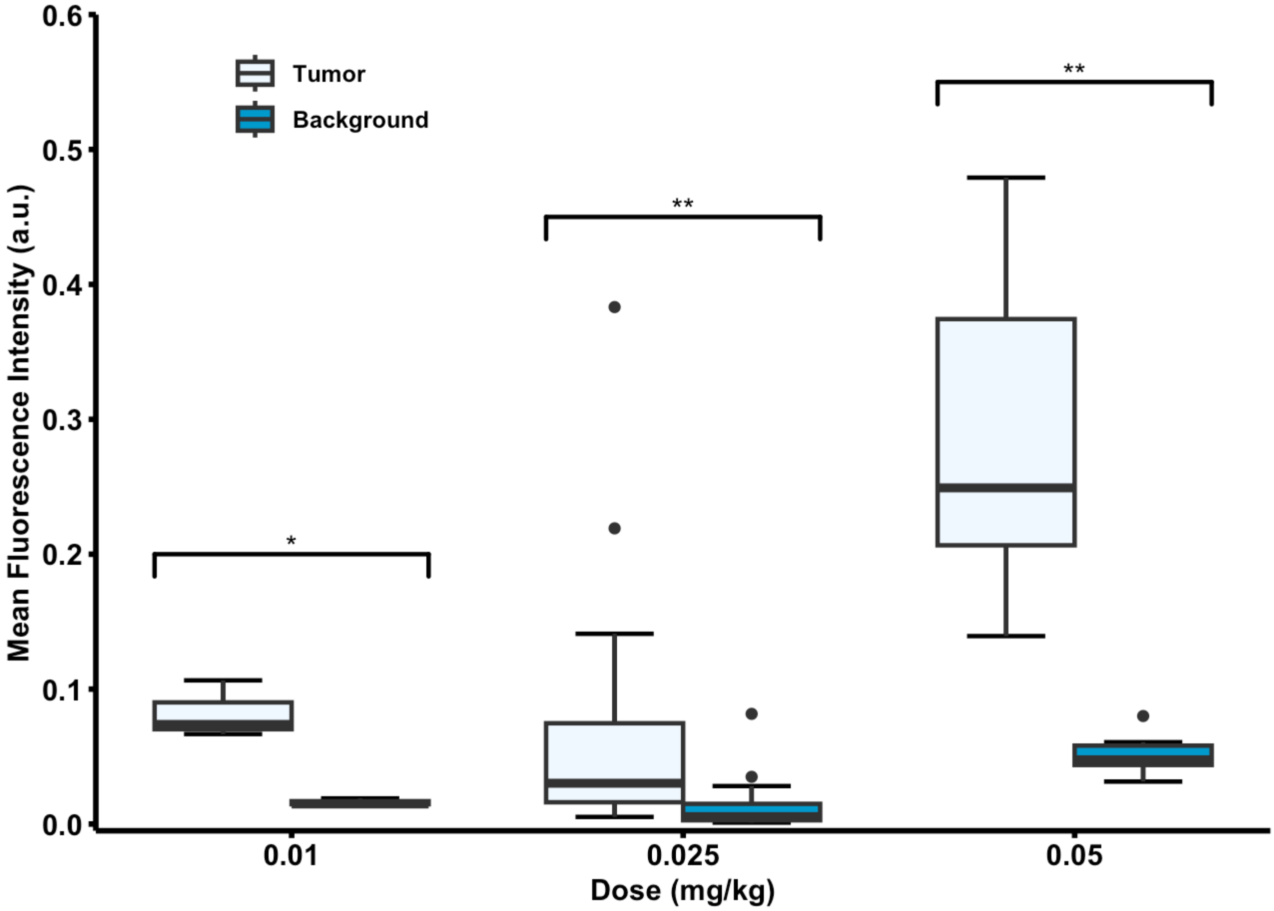

Supplementary Fig. 4. Fluorescence in salivary gland tissue. Circled fluorescent spots in deep margin of specimen indicate salivary gland tissue (A). H&E staining of salivary gland (B), strong signal in flatbed scanning fluorescence imaging of microscopy slide (C), immunohistochemistry indicates expression of integrin αvβ6 in the ductal cells of salivary gland tissue (D).

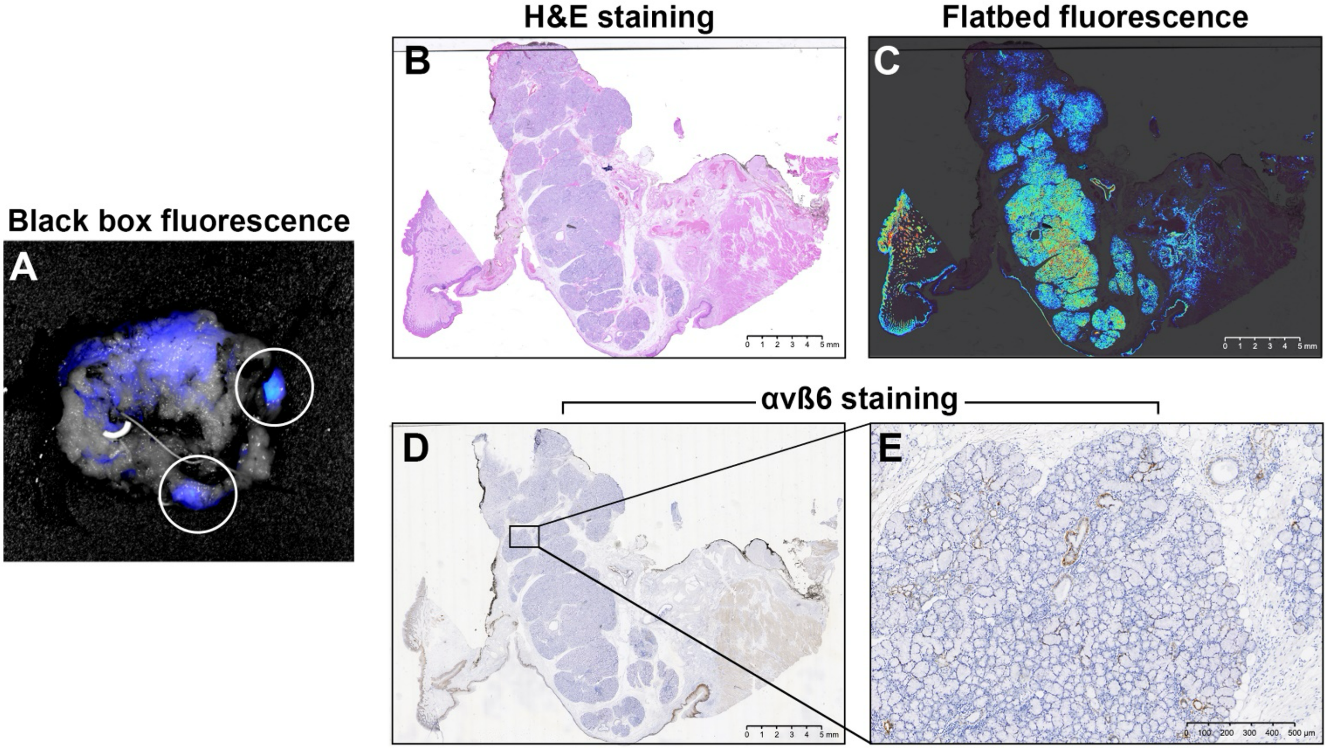

Supplementary Fig. 5. Fluorescence in bone. H&E staining of a slide containing tumor (black delineated), and bone (marrow) (green delineated). Moderate signal in bone marrow using closed-field fluorescence imaging of bread loaf (B). Strong expression of αvβ6 in tumor (D, E), whereas fluorescent bone (marrow) displayed none (C, D).

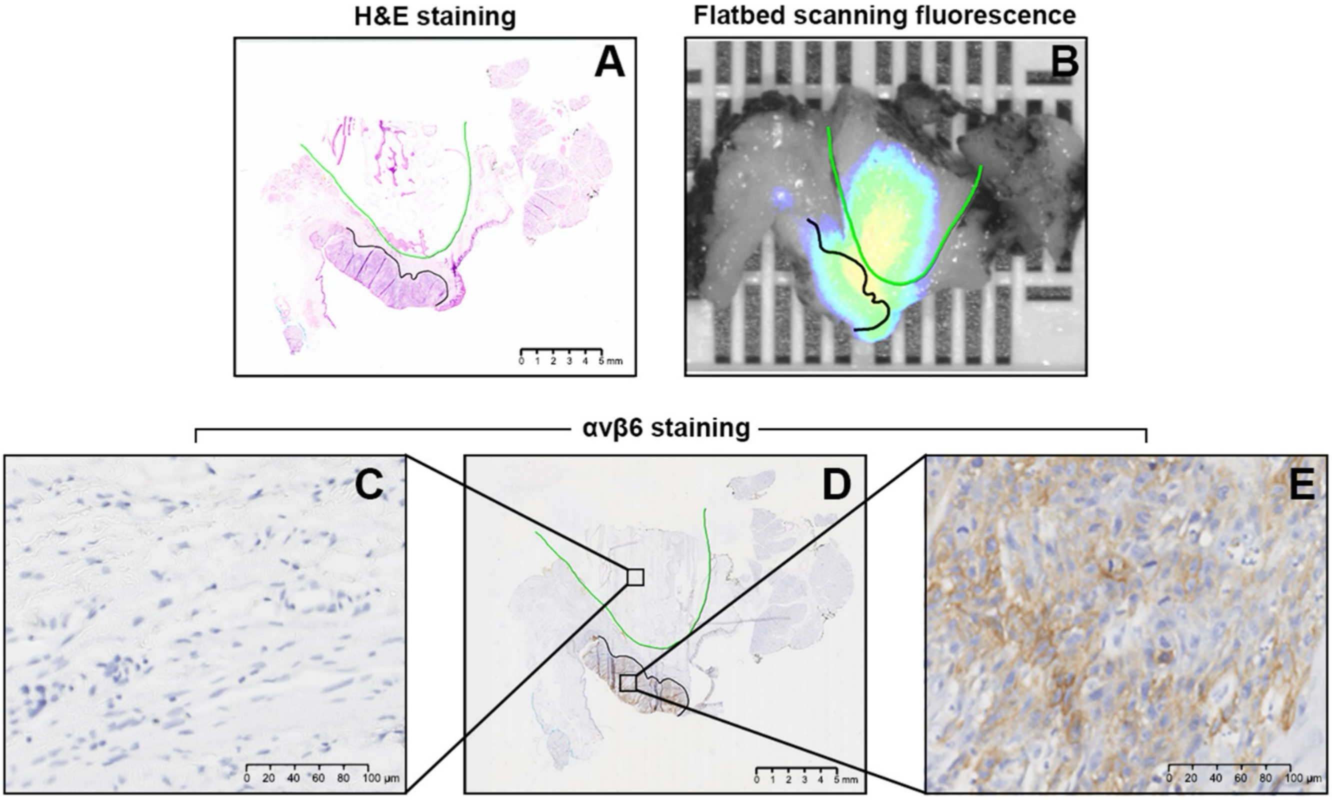

Supplementary Fig. 6. An example of a SFF emission spectrum collected from (a) the centre of tumor and (b) contralateral normal tissue 18h after tracer administration, that has been corrected for the tissue optical properties (ma and m´s) at the excitation wavelength and in the within the emission band of the fluorophore. Where the corrected data (red circles) is the fitted with a linear combination of in-vivo basis spectrum for c-RGD-800ZW-1 (orange dashed line, fitted as a skewed gaussian) determined from a large group of measurements with the tracer present, and the basis spectrum for background autofluorescence, (green line) which is a combination of tissue autofluorescence and background fluorescence in the spectroscopy system.

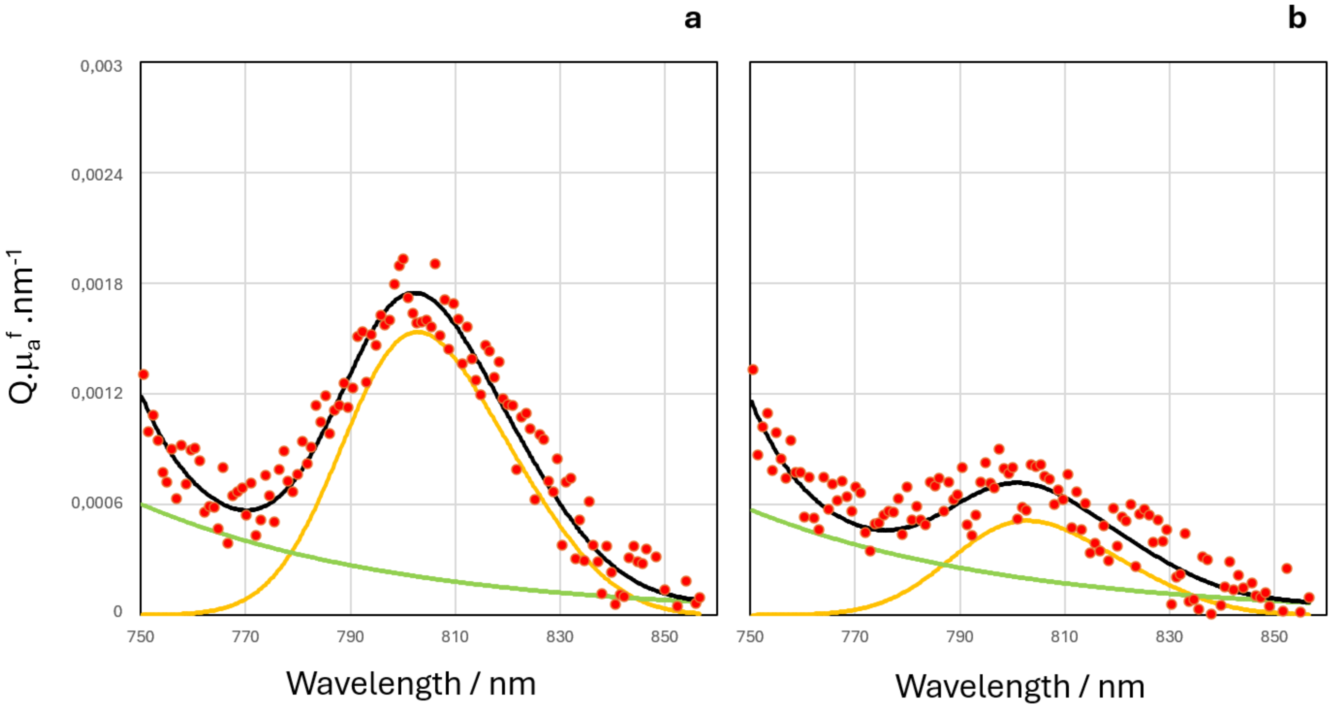

GUIDED BY LIGHT:

OPTIMIZING SURGICAL EXCISION OF ORAL CANCER USING REAL-TIME FLUORESCENCE IMAGING

**Protocol ID**	Guided by light: Optimizing surgical excision of oral cancer using real-time fluorescence imaging
**Short title**	Guided by light
**EudraCT number**	2019-003416-30

**Version**	3.0
**Date**	29-10-2020
**Project leader**	Dr. S. Keereweer, MD, PhD<br>Dept. of Otorhinolaryngology & Head and Neck Surgery, Erasmus MC<br>Dr. Molewaterplein 40, 3015 GD, Rotterdam
**Coordinating investigator**	ir. L.J. Lauwerends, PhD-candidate<br>Dept. of Otorhinolaryngology & Head and Neck Surgery, Erasmus MC
**Co-investigator(s)**	Dr. S. Koljenović , MD, PhD<br>Dept. of Pathology, Erasmus MC<br><br>Dr. A.L. Vahrmeijer, MD, PhD<br>Dept. of Surgical Oncology, Leiden University Medical Center<br><br>Dr. G. Puppels, PhD<br>Dept. of Dermatology, Erasmus MC
**Sponsor**	Erasmus MC<br>Dr. Molewaterplein 40<br>3015 GD Rotterdam
**Subsidising party**	KWF Kanker Bestrijding Delflandlaan<br>17<br>1062 EA Amsterdam
**Independent expert (s)**	Dr. M.P. van der Schroeff, MD, PhD Dept.<br>of Otorhinolaryngology, Erasmus MC
**Laboratory sites**	Dept. of Otorhinolaryngology & Head and Neck Surgery, Erasmus MC<br>Dept. of Pathology, Erasmus MC<br>AMIE and AMIEf Facility, Erasmus MC<br>Center for Optical Diagnostics and Therapy, Erasmus MC
**Pharmacy**	Erasmus Medical Center Pharmacy

TABLE OF CONTENTS

1. INTRODUCTION AND RATIONALE ... 9
2. OBJECTIVES ... 10
3. STUDY DESIGN ... 11
4. STUDY POPULATION ... 12
4.1 Population (base) ... 12
4.2 Inclusion criteria ... 12
4.3 Exclusion criteria ... 12
4.4 Sample size calculation ... 13
5. TREATMENT OF SUBJECTS ... 14
5.1 Investigational treatment ... 14
5.1.1 Preoperative procedure ... 14
5.1.2 Operational procedure ... 14
5.1.3 Parallel tagging procedure ... 15
5.1.4 Assessment of intraoperative fluorescence signals ... 17
5.1.5 Wound bed driven analysis ... 18
5.1.6 Specimen driven analysis ... 20
5.1.7 Postoperative tissue analysis ... 21
5.1.8 Postoperative follow-up ... 21
5.1.9 Fluorescence imaging ... 21
5.2 Use of co-intervention ... 22
5.3 Escape medication ... 22

6. INVESTIGATIONAL PRODUCT 23
6.1 Name and description of investigational product(s) 23
6.2 Summary of findings from non-clinical studies 23
6.3 Summary of findings from clinical studies 25
6.4 Summary of known and potential risks and benefits 27
6.5 Description and justification of route of administration and dosage 27
6.6 Dosages, dosage modifications and method of administration 28
6.6.1 Decision making to determine recommended dose 29
6.7 Preparation and labelling of Investigational Medicinal Product 29
6.8 Drug accountability 30
7. NON-INVESTIGATIONAL PRODUCT 31
8. METHODS 32
8.1 Study parameters/endpoints 32
8.1.1 Main study parameter/endpoint 32
8.1.2 Secondary study parameters/endpoints 32
8.1.3 Other study parameters 33
8.2 Randomisation, blinding and treatment allocation 33
8.3 Study procedures 33
8.4 Withdrawal of individual subjects 37
8.4.1 Specific criteria for withdrawal 37
8.5 Replacement of individual subjects after withdrawal 37
8.6 Follow-up of subjects withdrawn from treatment 37

8.7 Premature termination of the study 37
9. SAFETY REPORTING 38
9.1 Temporary halt for reasons of subject safety... 38
9.2 AEs, SAEs and SUSARs 38
9.2.1 Adverse events (AEs) 38
9.2.2 Serious adverse events (SAEs) 38
9.2.3 Suspected unexpected serious adverse reactions (SUSARs) 38
9.3 Annual safety report 39
9.4 Follow-up of adverse events 39
9.5 Safety Committee 40
10. STATISTICAL ANALYSIS 41
10.1 Primary study parameter(s) 41
10.2 Secondary study parameter(s) 42
10.3 Other study parameters 43
10.4 Interim analysis 43
11. ETHICAL CONSIDERATIONS 44
11.1 Regulation statement.. 44
11.2 Recruitment and consent 44
11.3 Objection by minors or incapacitated subjects 44
11.4 Benefits and risks assessment, group relatedness 45
11.5 Compensation for injury 45
11.6 Incentives 45
12. ADMINISTRATIVE ASPECTS, MONITORING AND PUBLICATION 46
12.1 Handling and storage of data and documents 46
12.2 Monitoring and Quality Assurance 46
12.3 Amendments 46
12.4 Annual progress report 47
12.5 Temporary halt and (prematurely) end of study report 47

12.6 Public disclosure and publication policy 47
13. STRUCTURED RISK ANALYSIS 48
13.1 Potential issues of concern 48
13.2 Synthesis 49
14. REFERENCES 50

LIST OF ABBREVIATIONS AND RELEVANT DEFINITIONS

**ABR**	General Assessment and Registration form (ABR form), the application form that is required for submission to the accredited Ethics Committee; in Dutch: Algemeen Beoordelings- en Registratieformulier (ABR-formulier)
**AE**	Adverse Event
**AR**	Adverse Reaction
**CA**	Competent Authority
**CCMO**	Central Committee on Research Involving Human Subjects; in Dutch: Centrale Commissie Mensgebonden Onderzoek
**CV**	Curriculum Vitae
**DSMB**	Data Safety Monitoring Board
**EU**	European Union
**EudraCT**	European drug regulatory affairs Clinical Trials
**GCP**	Good Clinical Practice
**GDPR**	General Data Protection Regulation; in Dutch: Algemene Verordening Gegevensbescherming (AVG)
**IB**	Investigator's Brochure
**IC**	Informed Consent
**IMP**	Investigational Medicinal Product
**IMPD**	Investigational Medicinal Product Dossier
**METC**	Medical research ethics committee (MREC); in Dutch: medisch-ethische toetsingscommissie (METC)
**(S)AE**	(Serious) Adverse Event
**SPC**	Summary of Product Characteristics; in Dutch: officiële productinformatie IB1-tekst
**Sponsor**	The sponsor is the party that commissions the organisation or performance of the research, for example a pharmaceutical company, academic hospital, scientific organisation or investigator. A party that provides funding for a study but does not commission it is not regarded as the sponsor, but referred to as a subsidising party.
**SUSAR**	Suspected Unexpected Serious Adverse Reaction

UAVG Dutch Act on Implementation of the General Data Protection Regulation; in Dutch: Uitvoeringswet AVG

WMO Medical Research Involving Human Subjects Act; in Dutch: Wet Medisch-wetenschappelijk Onderzoek met Mensen

SUMMARY

Rationale: Head and neck cancer (HNC) is the 9th most common tumor worldwide, a third of them arising in the oral cavity. Complete tumor resection of oral cancer is the most important surrogate marker for survival. Adequate tumor removal increases survival, lowers local tumor recurrence rates, and reduces the need for burdensome adjuvant therapies (chemotherapy and radiotherapy). Moreover, precise margin delineation is imperative in the delicate head and neck region where wider resections inevitably lead to increased morbidity and loss of functionality.
In current practice, intraoperative assessment of the tumor-free margin is dependent on visual appearance and palpation of the tumor. We have reported inadequate surgical margins in up to 85% of patients with oral cancer, which is unacceptable. Therefore, new intraoperative visualization techniques are required to assess tumor margins in real-time and to guide surgical removal of oral cancer with adequate tumor-free margins while retaining maximal functionality.

Objective: The overarching goal of this study is to improve adequate resection of oral cancer. This clinical trial will determine the optimal dose of cRGD-ZW800-1 and provide insight into the feasibility of intraoperative FLI to adequately assess tumor margins in patients with oral cancer.

Study design: This is a two-staged clinical trial to investigate the feasibility of intraoperative FLI to adequately assess tumor margins in patients with oral cancer using cRGD-ZW800-1. The first phase of the clinical trial centres on evaluating the TBR in patients with oral cancer using 2 dosages of cRGD-ZW800-1. The second phase evaluates if using cRGD-ZW800-1 to assess the margin status during surgery increases the percentage of operations yielding tumor-free margins in oral cancer.

Study population: Patients over 18 years of age, with biopsy-proven squamous cell carcinoma of the oral cavity, eligible for surgical resection of the primary tumor.

Intervention: In the first stage of the study, two groups of 7 patients each receive a different dosage of cRGD-ZW800-1 within 16-20 hours before surgery. In the second stage, after determining the ideal dose, 14 patients are added to the group that had received the ideal dose.

Main study parameters/endpoints:

1. To determine the mean intraoperative tumor-to-background ratio (TBR) of cRGD-ZW800-1 in patients with oral cancer;

2. To determine if using FLI can increase the rate of adequate (i.e. >5mm clear) tumor resection margins.

Nature and extent of the burden and risks associated with participation, benefit and group relatedness: The risks of participation for patients in the trial include adverse (hypersensitivity) reactions. These risks are deemed minimal. Nevertheless precautionary measures are in place, including supervised administration by qualified staff and availability of medical treatment to treat hypersensitivity reactions, and these effects are generally well manageable. Furthermore, patients are asked to consider joining scientific research at an emotionally heavy time of diagnosis and treatment of cancer, and will undergo additional measurements of vital functions, ECG and laboratory testing after administration. There is a small chance of higher surgical success rate.

1. INTRODUCTION AND RATIONALE

5-year survival in patients with oral cancer is less than 50%. With ~1000 new cases of oral cancer presenting each year in The Netherlands, this is a significant problem. The surgical margin status is the single most important surrogate marker for survival, and thus predictor for a good prognosis of patients with oral cancer. Yet, precise margin delineation is imperative in the delicate head and neck region because removing excessive healthy tissue is highly detrimental to quality of life. Surgery in this area can impair speech, swallowing, breathing, and/or appearance.

The surgeon determines the tumor-free margin based on visual appearance and palpation of the tumor. Although our oncological results are comparable to, or even better than, the international standards, we came to a shocking conclusion that 85% of our oral cancer patients had inadequate margins using standard surgical protocols [1]. Our findings have since been confirmed by other groups [2]. It is clear that a better method is needed to determine the tumor border.

Currently at the Erasmus MC, oral cancer resection specimens are extensively inspected intraoperatively by a dedicated pathologist and the surgeon. This led to a decrease of inadequate surgical margins to under 50% and clearly indicates the potential for improvement
[3]. In the current "gold standard", a pathologist generally analyzes less than 5% of the complete specimen surface. Wide adoption of our time-consuming laborious assessment by a team of specialists is therefore not realistic.

It is evident that new, objective, intraoperative visualization techniques are needed to guide surgical removal with adequate tumor-free margins. Fluorescence imaging (FLI) using near-infrared light has recently emerged as a revolutionary technique by providing real-time visualization of tumor tissue, enabling image-guided surgery [4, 5]. We systemically inject tumor-specific fluorescent targeting agents prior to surgery. During the operation, we perform real-time FLI using a dedicated camera system.

Since the first clinical pilot study, ground-breaking results have been reported in patients with other cancer types. In oral cancer (and HNC in general) >95% of the tumors arise from the mucous membranes, making these tumors ideal for surface imaging techniques. Extensive preclinical work was performed on FLI in HNC with very encouraging results [6-9]. This study translates preclinical work into clinical practice, in which the tumor-specific targeting of a new fluorescent agent will be tested for the first time in patients with HNC.

2. OBJECTIVES

The aim of this study is to investigate the feasibility of using intraoperative fluorescence imaging (FLI) to adequately assess the tumor margin in patients with oral cancer. This is a two-staged study (*Work Package I and II*), for which primary objectives are defined separately:

Primary Objectives:

To improve adequate resection of oral cancer using fluorescent imaging technology.

1. **WP I**: To determine the recommended dose for the highest tumor-to-background ratio (TBR) of at least >2.0 using cRGD-ZW800-1 in oral cancer;
2. **WP II**: To increase the rate of adequate (i.e. >5 mm clear) tumor resection margins

Secondary Objectives:

1. **WP I and II**: To assess safety and tolerability of single doses of cRGD-ZW800-1
2. **WP II**: To assess sensitivity, specificity, positive and negative predictive values of FLI;
3. **WP II**: To correlate integrin-specific targeting with immunohistochemistry;
4. **WP II**: To determine percentage of intraoperative change in surgical management based on imaging;
5. **WP II**: To determine the incremental operation time;
6. **WP II**: To assess FLI of excised cervical lymph nodes.

3. STUDY DESIGN

This is a two-staged clinical trial to investigate the feasibility of intraoperative FLI to adequately assess tumor margins in patients with oral cancer using cRGD-ZW800-1. The study design, sample size, dosing and time interval between injection and imaging are based on the preclinical and phase I results, as well as a trial that is currently still running using the same imaging agent. The plan of investigation is divided into two work packages (WP):

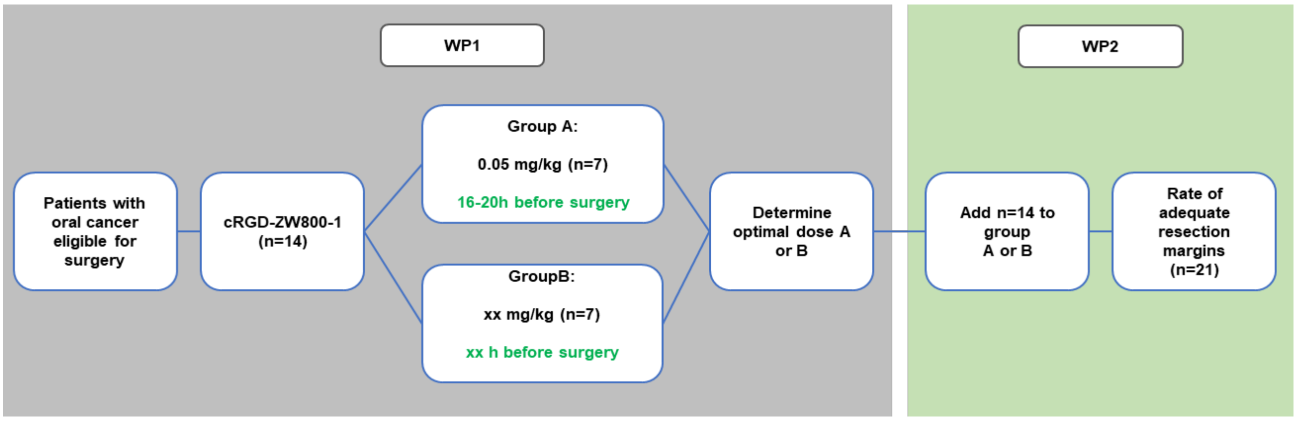

Figure 1 Study design: WP-I and WP-II

Work package I

In WP-I, the preferred dose of the agent for imaging of margins in oral cancer will be determined. The signal-to-noise ratio will be determined in dose group A (n=7), which will receive 0.05 mg/kg of the tracer, 16-20 hours before surgery. After an interim evaluation of this ratio, the second dose group B (n=7) will receive an adjusted dosage of the tracer. After inclusion of all patients (n=14), the dose with the highest intraoperative signal-to-noise ratio will be selected. Safety and tolerability of cRGD-ZW800-1 will be assessed continuously.

Work package II

In WP-II, an expansion cohort (n=14) will be added to the group of patients that had received the selected dose in WP-I. In this group of 21 patients, it will be determined if FLI can improve the rate of adequate surgical resection margins. As secondary research questions, the following aspects will be assessed:

- safety and tolerability of single doses of cRGD-ZW800-1
- sensitivity, specificity, positive and negative predictive values of FLI;
- colocalization with immunohistochemistry;
- change in surgical management; incremental operation time; □ FLI of excised cervical lymph nodes.

4. STUDY POPULATION

4.1 Population (base)

Patients diagnosed with squamous cell carcinoma in head and neck region, who will undergo surgical resection at the department of Otorhinolaryngology and Head and Neck Surgery of the Erasmus Medical Center Rotterdam will be invited to participate in this study. Potential patients will be selected during the weekly multidisciplinary meeting where all new head and neck cancer patients are discussed.

4.2 Inclusion criteria

In order to be eligible to participate in this study, a subject must meet all of the following criteria:

- Patients with biopsy-proven squamous cell carcinoma of the oral cavity, eligible for surgical resection of the primary tumor;
- ≥ 18 years of age;
- Before patient registration, written informed consent must be given according to ICH/GCP, and national/local regulations.

4.3 Exclusion criteria

A potential subject who meets any of the following criteria will be excluded from participation in this study:

- Previous surgery in the same area of the oral cavity, chemotherapy or radiotherapy to the oral cavity;
- Other synchronous biopsy proven malignancies currently active, except for adequately treated in situ carcinoma of cervix and basal or squamous cell skin carcinoma;
- History of a clinically significant allergy or anaphylactic reactions to any of the components of the agent.
- Patients pregnant or breastfeeding, lack of effective contraception in male or female patients with reproductive potential;
- Patients with renal insufficiency (eGFR<60);
- Patients with a previous kidney transplantation in the medical history;
- Patients using medications that may significantly impair renal function (i.e. NSAIDs, particularly COX-2 inhibitors);
- Patients with an ASA classification of 4 or higher;
- Patients with measured QTc of 500 ms or higher at screening; Patients with laboratory abnormalities defined as:
- Aspartate AminoTransferase, Alanine AminoTransferase, Gamma Glutamyl Transferase) or Alkaline Phosphatase levels above 5 times the ULN or;
- Total bilirubin above 3 times the ULN or;
- Platelet count below 100 x 109/L or;
- Hemoglobin below 4 mmol/L (females) or below 5 mmol/l (males).
- Immuno-compromised patients who do not have the ability to respond normally to an infection due to an impaired on weakened immune system, caused by either a pre-existing disease or concomitant medications.

4.4 Sample size calculation

<u>WP-I:</u>

A lower threshold to differentiate signal from background noise is set at a mean TBR of µ0=2.0. Based on previous preclinical studies using cRGD-ZW800-1 in vivo [5], and on

experience from clinical trials using similar tumor-specific fluorescent targeting agents [6, 7], the $\mu 1$ is set to a mean of 4.0 with a standard deviation σ of 1.5. This standard deviation is wider than what was found in similar studies in other tumors to correct for an anticipated larger variation in integrin expression levels in oral cancers compared to cancer types that were included in other trials. Considering an α of 0.05 and a power of 80%, the sample size (n) is 7 per group, based on the One-Sample 2-sided t-test.

WP-II:

One group of 7 patients that was analyzed in WP-I with the recommended dose will be included in the single-arm WP-II trial. The primary endpoint in WP-II is the rate of adequate surgical resection margins.

Significance level and power:

WP-II tests the null hypothesis that the adequate surgical margins rate is at most 15% versus the alternating hypothesis that the adequate surgical margins rate is at least 40%. An adequate surgical margin is based on the gold standard of histopathologic analysis and is defined as >5mm tumor-free margin from the tumor border, in accordance with the Royal College of Pathologists. The significance level (i.e., the probability of rejecting H0 when it is true) is $\alpha=0.05$ and the power (i.e., the probability of deciding the regimen is active) is 80%.

Based on the A'Hern single stage Phase II design, the expected sample size is 21. The minimal number of successes to indicate that the treatment is effective is 7.

5. TREATMENT OF SUBJECTS

See section ***8.3*** for an overview of all study procedures and the visit and assessment table.

5.1 Investigational treatment

5.1.1 Preoperative procedure

- Routine work-up for patients with oral cancer is performed according to the institutional protocol and (inter-)national guidelines.
- Eligible patients will provide written informed consent.
- Assessment of ASA classification by the anesthesiologist to make sure the patient is fit for surgery. If relevant, these data can be used to determine the Common Terminology Criteria for Adverse Events Version 5.0 (CTCAE) baseline characteristics.
- The patient will be admitted to the hospital 1 day prior to surgery.
- The study drug, cRGD-ZW800-1, will be administered in a bolus via the venous access line placed in preparation for surgery 16-20h prior to surgery via a slow infusion during approximately 5-10 minutes. After this, patients will be closely monitored using ECGs and repeated laboratory testing to monitor potential toxicity or allergic reactions.

- After drug administration, MDSFR/SFF (*Multi-diameter single-fiber reflectance/ single-fiber fluorescence*) spectroscopy measurements will be performed. This step method consists of probe point measurements (5 times 5 s each) at several time points to quantify the concentration of cRGD-ZW800-1 in/around the tumor over time. Measurements will be taken in the middle and on the edges of the tumor, and on the contralateral side (of the oral cavity).

5.1.2 Operational procedure

- MDSFR/SFF measurements will be performed before the operational procedure (*while still in the preoperative holding*).
- All operations are performed under general anesthesia.
- Total operation time will be determined to assess potential incremental increase in operation time compared to historical data.
- All procedural steps indicated in the flowcharts (Fig. 5 and 6) are explained in further detail below.

5.1.3 Parallel tagging procedure

In our institution, the standard-of-care procedure is to delineate the surgical cut based on visual aspect and palpation at the start of the operation. After the cut has been delineated, but before the start of the resection, numbered tags are applied in a pair-wise and consecutive manner on both the wound bed side and the specimen side of the surgical cut, as shown in Fig. 3. This procedure allows for relocation of potentially compromised areas to the surgical wound bed, which is an essential component of the current trial design.

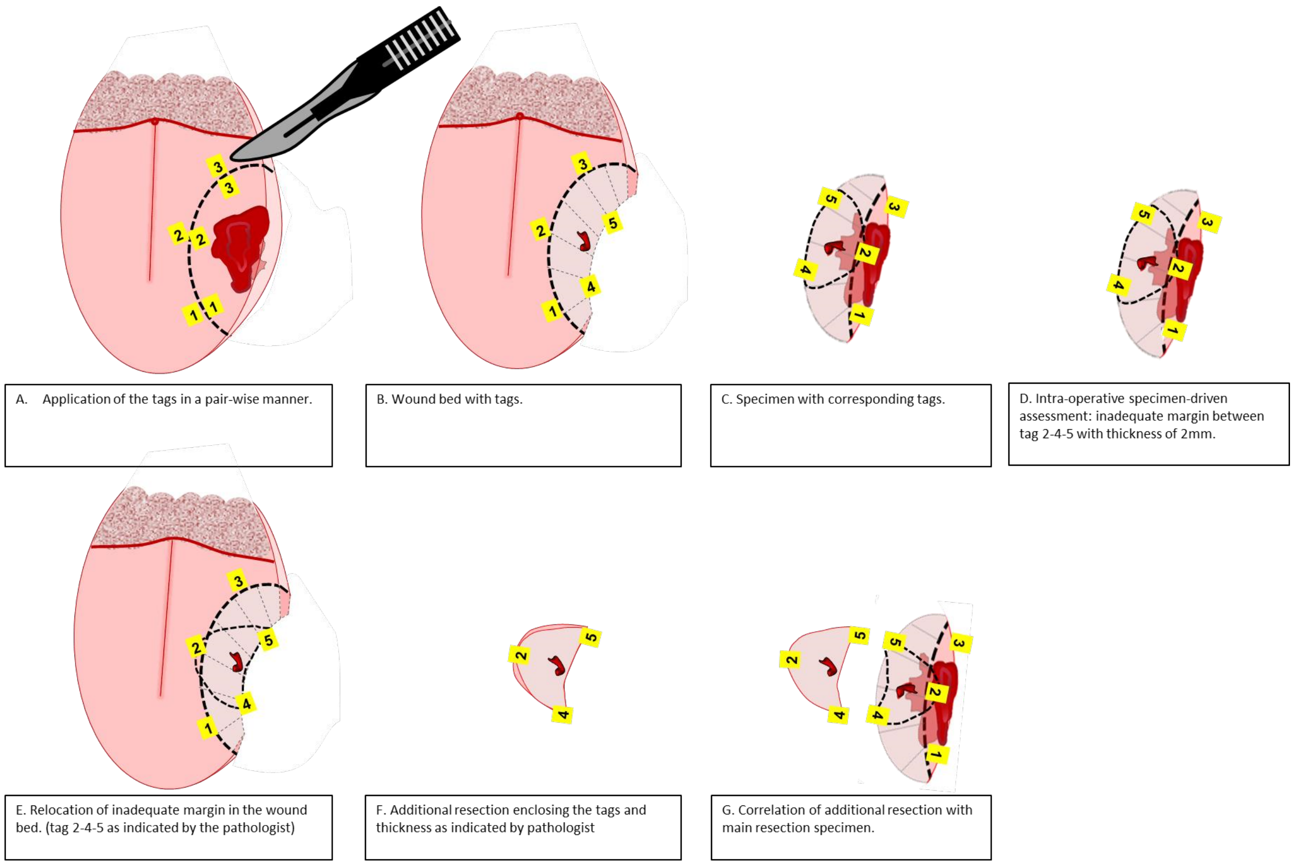

Figure 2: Schematic presentation of the parallel tagging procedure of the wound bed and specimen side of the surgical cut. These tags are used for relocation of the ROI after intraoperative specimen-driven assessment by the surgeon and pathologist together [3].

An example of the procedure in a patient with oral cancer is shown in Fig. 3.

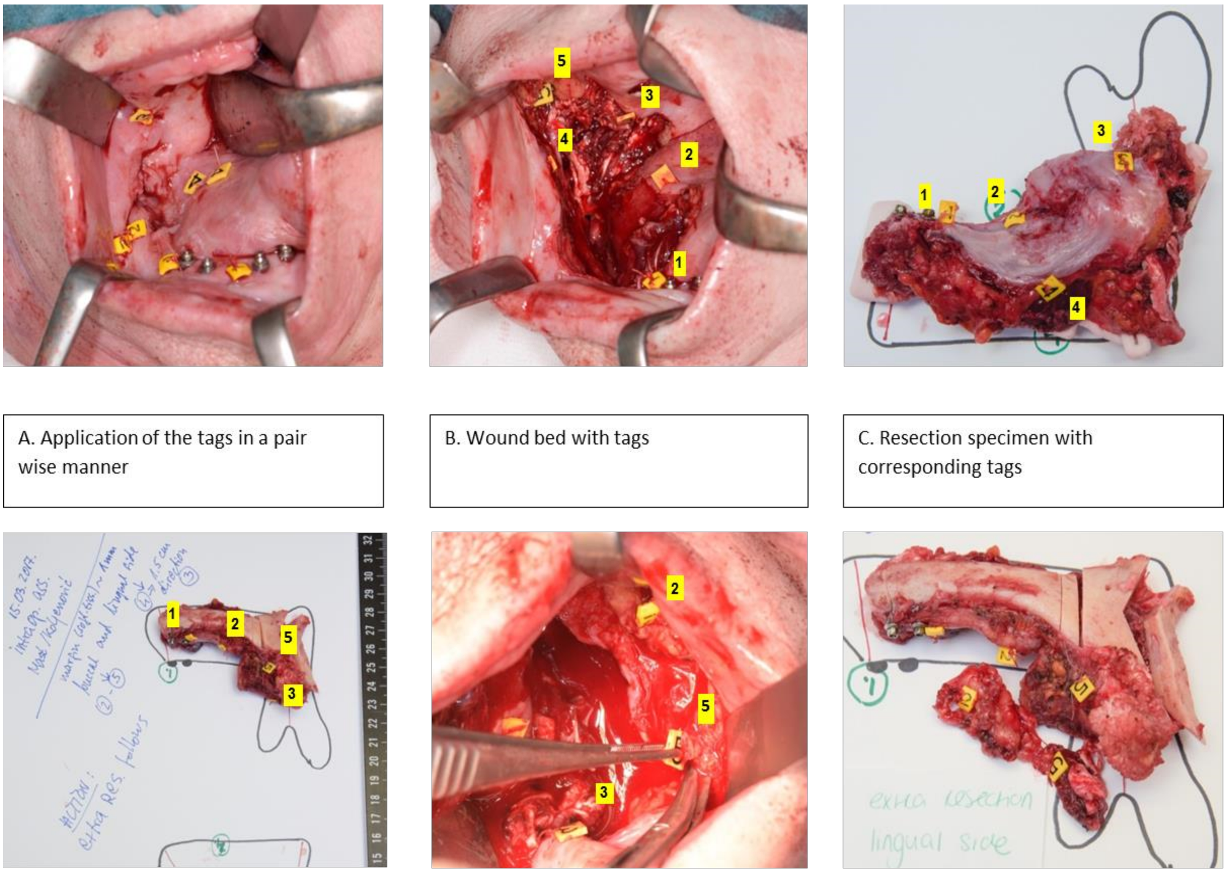

A. Application of the tags in a pair wise manner

B. Wound bed with tags

C. Resection specimen with corresponding tags

D. Specimen-driven intra-operative assessment: inadequate margin (1mm) between tags 2-5

E. Additional resection between tags 2 and 5, 5 mm in width and 8 mm in depth

F. Correlation of additional resection with main resection specimen

Figure 3. Example of the parallel tagging procedure in a patient with oral cancer. An additional resection was performed based on the intraoperative assessment and relocation of the ROI [3].

5.1.4 Assessment of intraoperative fluorescence signals

All operations are performed under general anesthesia. After the surgeon has indicated the surgical cut, the intraoperative tumor-to-background ratio (TBR) will be calculated. FLI will be performed of the tumor and surrounding healthy tissue using the dedicated intraoperative camera system. Snapshots and recordings will be made for TBR analysis using appropriate exposure times.

The TBR is defined as the largest difference in mean fluorescence intensity between tumor and surrounding healthy tissue. This will be assessed intraoperatively. The TBR will be calculated as follows:

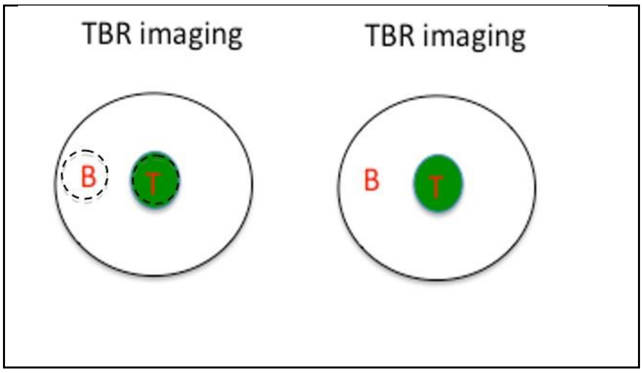

Figure 4. TBR calculation

To assess the TBR, fluorescence intensity will be calculated by drawing 5 circular regions of interest (ROI) (intermittent circle) in the tumor center and another 5 ROIs in the surrounding healthy tissue at a minimum of 1cm from the tumor front. Mean fluorescence quantified value of tumor and background region will be divided from each other to obtain TBR.

For every lesion, fluorescence status (yes/no) and tumor status (yes/no as determined by the golden standard pathology analysis) will be assessed.

Fluorescence intensity will be calculated during postoperative processing using an image-processing program, Image J. After resection, macro-segmentation of the fresh tumor specimen is performed and lamellated slices are imaged.

TBR is the only parameter that is used to evaluate WP-I. However, data from these patients will be used in WP-II and therefore the tumor bed and resection surface will be additionally assessed as indicated in WP-II.

5.1.5 Wound bed driven analysis

After conventional surgical resection, the wound bed and the specimen will be analyzed separately. In the wound bed driven analysis (Fig. 5), fluorescence imaging of the wound bed is performed. If no FLI is found in the wound bed, this suggests that no residual disease is present. In other study designs, this can only be confirmed after final pathology. In this study design however, we can safely say that no residual disease is present if adequate margins are found on IOA (what happens if these margins are inadequate, is further described in 5.1.6 Specimen driven analysis). Random biopsies of the FLI-negative wound bed are thus not needed, and no extra action is required.

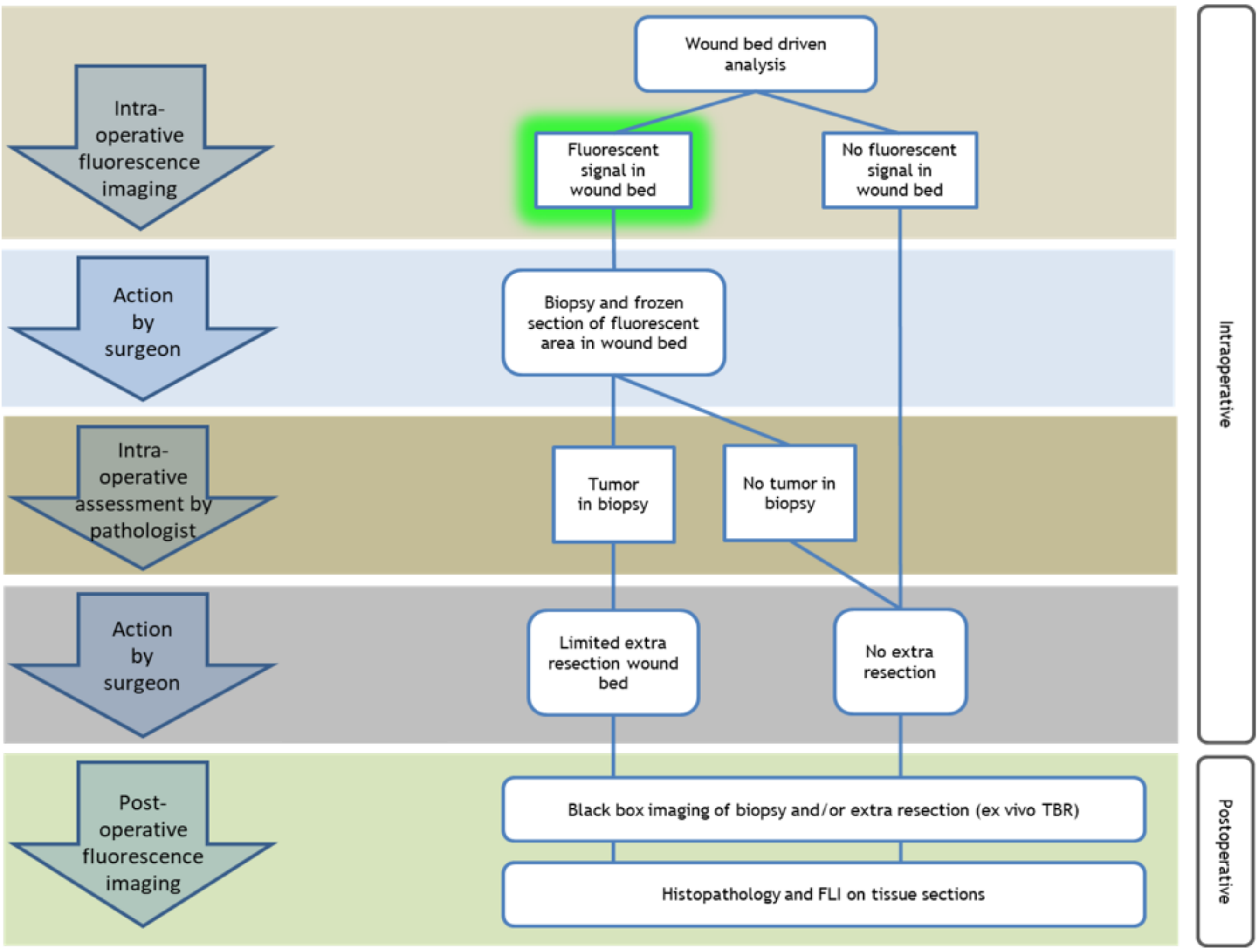

Figure 5. Flowchart wound bed driven analysis

If fluorescent signal is found in the wound bed, a biopsy of that area is taken by the surgeon for frozen section based on a high suspicion of residual tumor tissue. If no tumor is found in this biopsy, this tissue is further analyzed separately in a black box imaging system. However, if tumor tissue is found in this biopsy, a limited extra resection is performed by the surgeon and sent for separate histopathological analysis. This extra section is then relocated to the original specimen based on the parallel tagging. The resected tissue is also imaged using a black box imaging system and FLI on histopathology slides is performed in all cases.

<u>What if we never find fluorescent areas in the wound bed?</u>

Finish trial as planned. This is a possibility if adequate resection margins are consistently taken by the surgeon. The separate analysis of the specimen would still provide us with all information needed to successfully complete the primary endpoint. <u>What if we never find tumor in the fluorescent areas of the wound bed?</u>

Finish trial as planned. This is a possibility if the tracer is less specific than expected because it targets, for instance, inflammatory areas around the tumor. This would be

a very important conclusion for the future applicability of this tracer. However, the separate analysis of the specimen would still provide us with all information needed to successfully complete the primary endpoint.

What if we consistently find tumor in the fluorescent areas of the wound bed?

Finish trial as planned. This is a possibility if the tracer is highly specific and surgical resection is often incomplete. I believe we should still finish the trial first before we can conclude that instant fluorescence-guided resection does not lead to unnecessary resection of extra healthy tissue.

5.1.6 Specimen driven analysis

Meanwhile, the specimen driven analysis is performed in this patient (Fig. 6). First, I will perform FLI of the specimen surface. If no fluorescent signal is found on the specimen surface (Fig. 6 – right column), standard-of-care IOA of tissue margins together with the pathologist will be performed. Only if the resection is inadequate, the inadequate region is marked on the specimen surface for ex vivo correlation with FLI, and the surgeon performs a limited extra resection of the corresponding wound bed. Next, the specimen and resected tissue are imaged ex vivo and FLI is performed on histopathology slides.

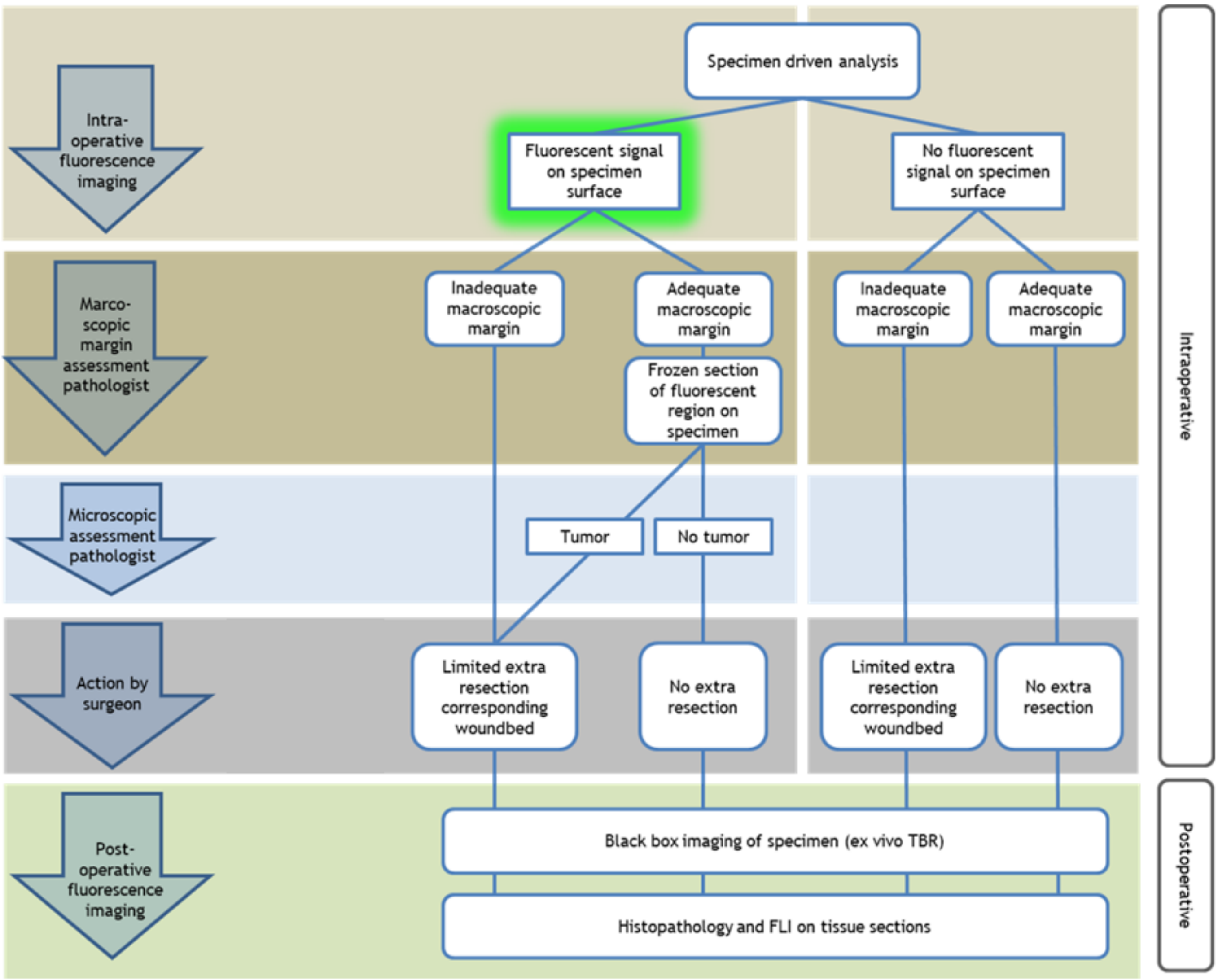

Figure 6. Flowchart specimen driven analysis

In case fluorescent signal is found at the specimen surface (Fig. 6 – left column), the(se) area(s) are marked with a suture. Next, we perform standard-of-care IOA together with the pathologist. If inadequate, the surgeon performs a limited extra resection of the corresponding wound bed.

However, if the specimen seems macroscopically adequate, we perform frozen section of the fluorescent area on the specimen. Only when this shows tumor, a limited extra resection is taken.
After this analysis, additional ex-vivo FLI of the specimen is performed postoperatively (Fig. 6 – bottom row), followed by macro-segmentation of the fresh tumor specimen and imaging of the lamellated slices without interference with standard histopathology. Finally, FLI on histopathology slides is performed in all cases.

In conclusion, this trial design allows to adequately assess if (residual) tumor is found in fluorescent areas in the wound bed or specimen. And the IOA protocol allows us to assess with much higher certainty that absence of fluorescence indeed corresponds with absence of tumor. These parameters are essential to reliably describe sensitivity, specificity, positive and negative predictive value of FLI (secondary research questions). But most importantly, this innovative method provides us with a direct tool to determine in how many cases FLI would change surgical management (i.e. dictate extra tissue resection) and thereby be of additional value for the surgeon (secondary research question).

5.1.7 Postoperative tissue analysis

This specimen as well as extra tissue sections that were taken from the wound bed (if any) are then analyzed separately using fluorescence (black box) imaging to determine ex vivo TBR. Next, histopathologic analysis is performed (gold standard) to determine surgical resection margins, growth pattern, and differentiation including immunohistochemistry for an integrin-specific marker. Finally, fluorescence imaging of the tissue slides is performed to determine location of the fluorescent targeting agent at a microscopic level, and to correlate fluorescent signal with immunohistochemistry.

5.1.8 Postoperative follow-up

- The end of study is defined as the date of 1st outpatient postoperative follow-up visit (approximately 2 weeks after discharge).
- Adverse events will be recorded throughout the study period.
- There are a total of 2 follow-up visits for recording of any adverse events and a medical history update:

1. on the day of discharge from the hospital;
2. on the day of the 1st out-patient post-operative follow-up visit.

5.1.9 Fluorescence imaging

The NIR camera system that will be used during the trial is the CE-marked “Spectrum Platform” system provided by the company Quest Medical Imaging (Netherlands). This platform provides:

- A handheld camera that is ready for open surgery when combined with the provided ring light and lens.
- A dedicated light engine that produces a highly optimized light bundle. The configuration of the light engine in combination with the camera will be optimized for the cRGD-ZW800-1 fluorophore.
- A media center that offers 2 Terabytes of raw data storage.
- A camera arm that allows stabilization of the camera.
- A trolley, adapted to operating room use.
- A surgical screen that displays fluorescence and allows easy control by the surgical team or the surgeon itself.

The Quest Spectrum platform is already used in current practice at the Erasmus Medical Center and is registered at the department of Medical Technology (Ultimo number: 144251, 144252, 144253, 144390).

6.2 Use of co-intervention

Not applicable

5.3 Escape medication

In the rare but possible case of allergic reaction/hypersensitivity to the injected agent, the following medication can be used (depending on severity of the allergic reaction):

- Anti-histamine (i.e. cetirizine 10 mg PO)
- Dexamethasone 4-8 mg PO/IV
- Tavegil 2 mg IV
- Adrenalin 0.5 mg IM (in case of deterioration: another dose of 0.5 mg IM)
- Salbutamol 5 mg nebulization (in case of bronchospasm)

6. INVESTIGATIONAL PRODUCT

6.1 Name and description of investigational product(s) cRGD-ZW800-1 is a fluorescent contrast agent that specifically binds to integrins associated with neoangiogenesis. It is a cyclic pentapeptide (cRGD) conjugated to the 800 nm NIR fluorophore ZW800-1 [10]. The cyclic 3-amino acid sequence (RGD) is clinically a well-known peptide that binds to various integrins (αvβ1, αVβ3, αvβ5, αvβ6, αvβ8, α5β1, α8β1 and αIIbβ3), mostly associated with neoangiogenesis. Tumors larger than 1-2 mm depend on the formation of new blood vessels to acquire sufficient amounts of oxygen and nutrients [11]. Some of these integrins are overexpressed on malignant cells and in tumor stroma, such as in breast, colorectal, pancreas and lung cancer [12]. RGD based molecules have already been investigated in various phase I and phase II imaging studies using PET and SPECT and in a phase III study as an anticancer therapy (cilengitide) [13-20].

6.2 Summary of findings from non-clinical studies

For detailed information reference is made to the Investigator's Brochure of cRGD-ZW800-1. Below is a summary with the most important information.

Non-clinical pharmacology

Binding affinity of cRGD-ZW800-1

The binding affinity and specificity of cRGD-ZW800-1 was validated in vitro and in vivo by competition experiments. All tumors express at least one of the RGD binding integrins. Therefore, a competition experiment was chosen to show binding affinity. Blocking the integrins with an excess of 200 times more unlabeled cRGD resulted in significantly lower tumor-to-background ratios at 4 and 24 hours (1.8 vs. 3.2, p=0,01 and 1.4 vs. 3.1, p=0.004, respectively). Thus, cRGD-ZW800-1 is specific for cRGD-binding integrins.

Biodistribution of cRGD-ZW800-1

Biodistribution was studied at 4 and 24 hours post injection in tumor bearing mice intravenously injected with doses ranging from 0 to 30 nmol cRGD-ZW800-1. Increasing doses resulted in significantly increasing fluorescence signals in the urine/bladder, liver, kidneys, small intestines, tumor and skin. Relatively more fluorescence is present in the liver and the kidneys at 24 hours compared to 4 hours due to their metabolizing functions. In addition, the skin showed also higher signals at 24 hours.

Non-clinical pharmacokinetics and metabolism

PK was measured in blood in mice using fluorescence imaging and a calibration curve. The mean maximal concentration measured after 1 minute was 1.17 µM, measured fluorescent values were validated by a predefined calibration curve (Figure S7). The area under the curve (AUC), calculated via trapezoid rule, was 38.9±8.0. Clearance was 0.25±0.05 mL/min. After an initial distribution phase (half-life 14.6±1.5 min), pharmacokinetics of cRGD-ZW800-1 was linear (half-life 25.3±5.7 min).

Hydrolysis of the ether linkage of ZW800-1 causes a shift of its absorbance peak (i.e. excitation). Absorbance at 770 nm in water at 37^0 C decreased 4% at 4h, while biological and physiological factors in human serum caused a decrease in fluorescence of 20%. Highest decrease was seen in rats: 44%.

Non-clinical toxicology and safety pharmacology

Toxicology of cRGD-ZW800-1

A generic extended single dose intravenous non-clinical toxicity study was performed in rats including control groups and two treatment groups with doses 15.0 mg/kg and 5.0 mg/kg cRGDZW800-1 according to ICH M3 (R2), FDA, EMEA and GLP regulations. Both doses groups consisted of 10 males and 10 females which were subjected to a full post mortem examination on day 2 post dosing. In addition, 5 recovery animals per sex were added in the control and mid dose group and were followed for 14 days post dosing.

No treatment-related mortality occurred in this study. Discolored (green) urine was observed in male and females on the day of treatment with 5.0 or 15.0 mg/kg. This finding was still present after 2-6 days (5.0 mg/kg). No other clinical signs, changes in clinical pathology or macroscopic and microscopic alterations were seen.

Based on these results, it is concluded that a single dose of 5.0 mg/kg and 15.0 mg/kg cRGDZW800-1 is well tolerated in rats. Because no recovery group existed in the 15.0 mg/kg group, the NOAEL will be set on 5.0 mg/kg. Scaling from rats to humans based on body surface area (BSA) results in a human equivalent (HED) dose of 0.81 mg/kg.

Toxicology of the ZW800-1 label

Preclinical toxicity studies were conducted to characterize the toxicity of ZW800-1. Specific studies to characterize the safety profile of ZW800-1 included single infusion range-finding toxicity studies in rats and dogs to support definitive single IV infusion toxicity study in rats (with a genotoxicity and functional observation assessment), and single dose toxicity study in dogs. A single IV infusion study to evaluate cardiovascular and respiratory function in conscious telemetered dogs and a bacterial reverse mutation assay were also performed. Studies were conducted using lyophilized powder as a trifluoroacetic acid salt. Detailed descriptions of these studies and their findings are provided in the addendum.

Based on the results of the single dose range-finding toxicity in rats, a single administration of ZW800-1 of 1, 3 or 10 mg/kg via IV infusion over 2 hours at 10 mL/kg/h to Sprague Dawley rats was well tolerated and did not result in any dose-limiting target organ toxicity. The mild increases in AST, ALT and bilirubin may indicate liver is a target organ for extremely high doses.
Based on the results of the single dose toxicity study of ZW800-1 in rats, the NOAEL in rats given as a single IV infusion is 24.5 mg/kg, and the MTD is 1000 mg/kg. ZW800-1 given as a single IV infusion at 1500 mg/kg to rats is above the MTD.

A single administration of 0.84, 6.25 or 12.5 mg/kg ZW800-1 to Beagle dogs resulted in no adverse effects as measured by clinical signs, body temperature, body weights and hematology, clinical chemistry, and coagulation parameters. Blue/dark blue urine attributed to ZW800-1 excretion was observed on the day of dosing in both males and females at 6.25 and 12.5 mg/kg. Therefore, it is concluded that a dose of 12.5 mg/kg in a definitive single-dose toxicity study of ZW800-1 in Beagle dogs should not result in any dose-limiting target organ toxicity.

Based on a single dose toxicity study in Beagle dogs including doses of 0, 7.0, 14.0 and 280.0 mg/kg, the 14.0 mg/kg dose was considered to be the NOAEL.

Administration of a single, IV, 30 min. infusion of ZW800-1 at doses of 0.7 and 7.0 mg/kg did not elicit any acute effects on the cardiovascular, pulmonary, or body temperature parameters monitored in this study. The observation of discolored urine was attributed to the elimination of the green test article and was not deemed adverse. The NOAEL was 7.0 mg/kg.

The mutagenic and clastogenic potential of ZW800-1 was assessed using 3 approaches: an in vitro test in bacterial mammalian cells and in vivo assays in rats for micronuclei using hematopoietic cells, and Comet assay for assessment of DNA strand breakage in liver cells were employed. There was no evidence of genotoxicity using any of the 3 assay systems.

6.3 Summary of findings from clinical studies

For detailed information reference is made to the Investigator's Brochure of cRGD-ZW800-1. Below is a summary with the most important information.

Hypothetically, all solid tumors express the previously mentioned integrins when they grow beyond 2 mm in diameter. Extensive preliminary work on cRGD-ZW800-1, a NIR fluorophore targeting integrins, has been performed and showed clear delineation of melanomas and colorectal, liver, pancreatic, lung, and head and neck tumors in xenograft mouse models [21-23].

A first-in-human phase 1, single ascending dose, randomized, placebo-controlled study has been performed in 11 healthy volunteers to determine the safety, tolerability and pharmacokinetics of cRGD-ZW800-1 intravenous injection. The following doses were investigated: 0.001 mg/kg and 0.005 mg/kg cRGD-ZW800-1. Safety was assessed by recording pre-administration events, adverse events (AEs), clinical laboratory parameters, vital signs, ECGs, physical examination and injection site monitoring. Pharmacokinetics was assessed by collecting blood and urine samples at defined time points. NIR fluorescence imaging of the hand was performed frequently to assess uptake and clearance patterns of cRGD-ZW800-1 in the skin.

None of the healthy volunteers showed signs of acute or chronic toxicity, and in particular no hypersensitivity reactions were encountered. During the study no significant clinical changes were seen in the volunteers after dosing. There were no evident clinical effects on the supine systolic and diastolic blood pressure or heart rate after dosing. The ECGs did not show clinically significant changes after dosing and no untoward effects on any analysis of blood chemistry or hematology were observed.

cRGD-ZW800-1 concentrations were measurable up to 6 hours post dose in the 0.005 mg/kg group. In the lower dose group (0.001 mg/kg) cRGD-ZW800-1 concentrations were measureable to up to 1 hour post dosing. Samples measured after these time points were below the detection limit. The cumulative excretion of cRGD-ZW800-1, expressed as the percentage of the injected dose, decreased with increasing dose. In the lowest dosing group (0.001 mg/kg) an average cumulative excretion of 83% cRGD-ZW800-1 was observed at 8 hours post dosing. In the higher dosing cohort, 0.005 mg/kg, the average cumulative excretion of cRGD-ZW800-1 8 hours post dosing was 68%. The decrease in excretion in higher doses can be explained by the breakdown of the NIR fluorophore ZW800-1 over time, which is known to occur in blood.

Table 1 PK parameters 0.001 mg/kg cRGD-ZW800-1

Parameter	*n*	*Mean*	*Median*	*SD*	*CV*	*Min*	*Max*
Cmax (ng/ml)	3	7.52	7.42	0.63	8.39	6.94	8.19
tmax (h)	3	0.06	0.05	0.03	56.77	0.03	0.10
CL_F (L/h)	3	15.18	15.19	3.85	25.38	11.32	19.03
V_F (L)	3	15.18	14.39	5.40	35.59	10.22	20.93
Term (h)	3	0.70	0.76	0.21	30.41	0.47	0.88
AUC_0_last (ng*h/ml)	3	3.15	3.49	0.74	23.48	2.31	3.67
AUC_0_inf (ng*h/ml)	3	5.04	4.84	0.81	16.13	4.35	5.94
PercAUCExtrap (%)	3	37.50	41.30	11.87	31.64	24.20	47.00

Table 2 PK parameters 0.005 mg/kg cRGD-ZW800-1

Parameter	*n*	*Mean*	*Median*	*SD*	*CV*	*Min*	*Max*
Cmax (ng/ml)	3	34.71	38.52	7.81	22.49	25.73	39.88
tmax (h)	3	0.04	0.05	0.01	21.65	0.03	0.05
CL_F (L/h)	3	10.14	10.78	2.75	27.09	7.12	12.50
V_F (L)	3	47.25	51.42	7.38	15.63	38.72	51.60
Term (h)	3	3.31	3.31	0.45	13.70	2.86	3.77
AUC_0_last (ng*h/ml)	3	28.36	28.38	6.20	21.87	22.15	34.55
AUC_0_inf (ng*h/ml)	3	37.50	36.82	7.66	20.44	30.19	45.48
PercAUCExtrap (%)	3	24.50	24.00	1.90	7.76	22.90	26.60

Interestingly, one human volunteer in the 0.005 mg/kg cohort had a recent cut with a scab on the hand present at the time of cRGD-ZW800-1 injection. This study subject had measurable fluorescence at the site of the lesion, which became visible within 15 minutes after dosing and remained above background up to 24 hours. Because integrins are known to be upregulated in neovasculature, and neovascularization occurs at the site of skin injury, the result from this study subject provides indirect evidence that cRGD-ZW800-1 is hitting its target.

A first-in-human phase II feasibility study was performed in 12 patients undergoing an elective colon resection (11 patients underwent minimally invasive surgery (MIS), 1 patient underwent open surgery) to determine the optimal dose and injection time window of cRGD-ZW800-1 for the intraoperative imaging of colon cancer. The following doses were investigated: 0.005, 0.015 and 0.05 mg/kg cRGD-ZW800-1.

The intraoperative TBR ranged from 1.1 in the patients who received 0.05 mg/kg 2 to 4 hours prior to surgery, to 1.6 in the patients who received 0.05 mg/kg 18 hours prior to surgery. No fluorescence through bowel wall was visible during the MIS procedures in the lower doses of 0.005 and 0.015 mg/kg. However, 1 patient in the lowest dose level (0.005 mg/kg) underwent an open colon resection where tumor fluorescence was visible through bowel wall with the open imaging system, with an intraoperative TBR of 1.5.

Longer intervals between injection and imaging improved the tumor-to-background ratio. The doses 0.005, 0.015, and 0.05 mg/kg 2 to 4 hours prior to surgery showed a mean ex

vivo TBR of 1.4, 4.0, and 4.1, respectively. The dose 0.05 mg/kg 18 hours prior to surgery displayed the highest ex vivo TBR of 6.2. The TBR increase was significant among doses 0.005 and 0.015 mg/kg (P = 0.031), and dose 0.005 and 0.05 mg/kg 18 hours (P =0.004). The ex vivo results coincide with the intraoperative TBR, which was also the highest in the patients with a longer injection time window of 18 hours (1.1 in 2–4 hours vs. 1.6 in 18 hours).

All patients had true-positive colon tumors (n=12). All the resected colon tumor specimens contained malignancy, showed a strongly positive αvβ6 expression (score 9–12) and displayed intense fluorescence on a microscopic level. Normal mucosa expressed milder αvβ6 expression (score 2–4) and accordingly exhibited less, to minimal, fluorescence when compared with the tumor (Fig. 7).

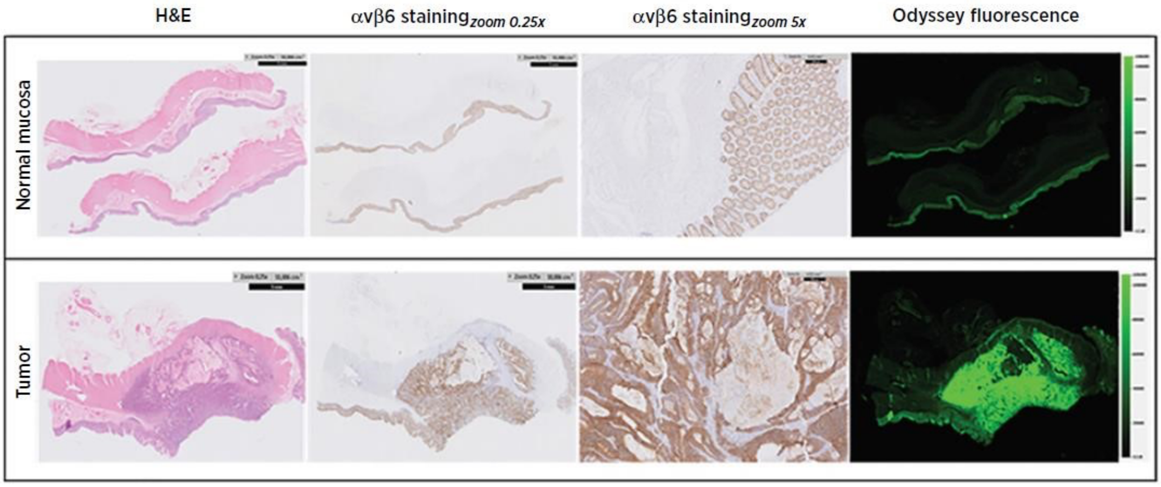

Figure 7. IHC staining of αvβ6 on formalin-fixed paraffin-embedded colon tumor tissue and normal mucosa. Images were obtained with the Odyssey CLx Imaging system. The colon tumor shows a positive strong αvβ6 expression and fluorescence, whereas normal mucosa shows a milder αvβ6 expression with intensely less fluorescence (same fluorescence settings).[24]

6.4 Summary of known and potential risks and benefits

For detailed information reference is made to the Investigator's Brochure of cRGD-ZW800-1. Also see chapter 13 for a structured risk analysis including all study details.

6.5 Description and justification of route of administration and dosage

A first-in-human phase 1, single ascending dose, randomized, placebo-controlled study was performed in 11 healthy volunteers to determine safety, tolerability and pharmacokinetics of cRGD-ZW800-1 intravenous injection.*[24]* The following doses were investigated: 0.001 mg/kg and 0.005 mg/kg. None of the healthy volunteers showed signs of acute or chronic toxicity, and in particular no hypersensitivity reactions were encountered. There were no evident clinical effects on the blood pressure or heart rate after dosing, no changes in ECG or changes of blood chemistry.

All these preliminary findings led to the initiation of a first-in-human clinical trial in patients with colorectal cancer at the LUMC (Fig 8).[24] No serious toxicity was reported

throughout the study. The doses included 0.005, 0.015, and 0.05 mg/kg. Regardless, a dose finding trial for oral cancer is necessary because colorectal cancers are different tumor types with different features and “behavior”. This influences integrin expression levels which may influence TBR.

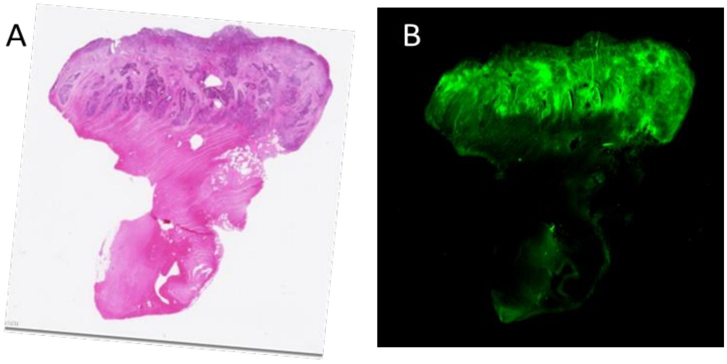

Figure 8. Preliminary data of first-in-human study of cRGD-ZW800-1 for intraoperative fluorescence imaging of colorectal cancer. A. HE staining of colorectal tumor. B. Colocalization of fluorescence signal within the tumor tissue, low signal is found in surrounding healthy tissue. (Unpublished data)

Although TBR may be different from colorectal cancer, the time interval between injection and imaging is mostly dependent on pharmacokinetics and pharmacodynamics and washout from healthy surrounding tissues. There is no reason to assume that this will be significantly different in oral cancer so the same time intervals from the phase I and colorectal cancer trials will be used in this study.

Based on the results of the phase II trial in patients with colorectal cancer, we will start with a dose of 0.05 mg/kg with injection time of 16-20h prior to surgery. In this study, the dose 0.05 mg/kg 18 hours prior to surgery displayed the highest ex vivo TBR of 6.2, which was significant among dose 0.005 and 0.05 mg/kg (P=0.031), and dose 0.005 and 0.05 mg/kg 18 hours (P=0.004). The intraoperative TBR was also highest in the patients with a longer injection time window of 18 hours (1.1 in 2-4 hours vs. 1.6 in 18 hours. Based on the results of this group, we will test the second group with either a higher or lower dose/time interval. Investigation of higher (than 0.05 mg/kg) dosages are planned at the LUMC. Increasing the dosage – in case the results of the first group indicate it necessary – will <u>only</u> take place after no toxicity is reported in these patients.

6.6 Dosages, dosage modifications and method of administration

The agent will be administered in a bolus via the venous access line placed in preparation for surgery 16-20h prior to surgery via a slow infusion during approximately 5-10 minutes. After this, patients will be closely monitored using ECGs and repeated laboratory testing to monitor potential toxicity or allergic reactions.

WP-I consists of 14 patients. The first group will receive 0.05 mg/kg cRGD-ZW800-1. (n=7, Fig. 1). After evaluation of the TBR results, we will test a second group with either a higher or lower dose/time interval. In WP-II, 14 patients will be added to the chosen dose/time interval group.

6.6.1 Decision making to determine recommended dose

After inclusion of all patients in dosing group A (n=7), the TBR results will be analyzed and discussed in the steering committee. A lower limit mean TBR of ≥2.0 is considered successful; a mean TBR of 4.0±1.5 is anticipated. If the mean TBR is ≥2.0 in this dosing group, de-escalation of the dosage and/or adjustment of the time interval is performed for group B (n=7). If the mean TBR is <2.0 in this dosing group, the dose for group B will be increased.

Information on TBR, concentration (through MDSFR/SFF) and safety of group A will be carefully analyzed to make an informed choice on the exact dose (de-)escalation and/or time interval adjustment for group B.

<u>What if the mean TBR is ≥ 2.0 or <2 in both groups?</u>

If the mean TBR is ≥ 2.0, the recommended dose for intraoperative imaging will be determined based on a combined appraisal of the highest mean TBR, safety and tolerability, potential interference with other experimental optical techniques, and costs. This will be a joint decision by the steering committee.

If the mean TBR was <2 for the first group, TBR results of the second group need to be closely monitored during the trial. After inclusion of the first 3 patients, an intermediate evaluation by the steering committee will assess if WP-I is on track of delivering its endpoint of a TBR of ≥ 2.0 in one of the cohorts. If the results seem to fall short – i.e., TBR remains <2 throughout dose group A and B - we will reconsider time interval and have the possibility to include a third cohort (n=7) with a higher dosage.

6.7 Preparation and labelling of Investigational Medicinal Product

The study drug cRGD-ZW800-1 will be supplied to the study site research pharmacist in vials of 0.725 mg each. The vials are packaged and labelled by the Peptide Laboratory of the Interdivisional GMP-Facility LUMC (IGFL), part of the department of Clinical Pharmacy and Toxicology, Leiden University Medical Center, Albinusdreef 2, Leiden, the Netherlands in accordance with local regulations. The dispensing of the study drug will be performed by the Erasmus Medical Centre Pharmacy, bearing a label (see below) with the identification required by local law, the protocol number, drug identification, and dosage.

**Voor intraveneuze injectie**
Onderzoeksmedicatie cRGD-ZW800-1 <u>0.050 mg/kg</u><br>**cRGD-ZW800-1 …… mg**<br>Toedienen als intraveneuze bolus injectie Bewaren tussen 2-8°C<br>Niet gebruiken na:…………….. ……..uur Proefpersoon: ………<br>Gewicht: ……….. kg<br>Onderzoeker: Dr. S. Keereweer<br>Apotheek Erasmus Medisch Centrum
**Voor intraveneuze injectie**

Onderzoeksmedicatie cRGD-ZW800-1 0.000* mg/kg
**cRGD-ZW800-1 …… mg**
Toedienen als intraveneuze bolus injectie Bewaren tussen 2-8°C
Niet gebruiken na:……………… ……..uur Proefpersoon: ………
Gewicht: ……….. kg
Onderzoeker: Dr. S. Keereweer
Apotheek Erasmus Medisch Centrum

* *The dosage for the second dose group is to be defined after an evaluation of the first dose group.*

6.8 Drug accountability

Drug accountability will be maintained by the Erasmus Medical Centre Pharmacy and assessed by maintaining adequate study drug dispensing records. The investigator is responsible for ensuring that dosing is administered in compliance with the protocol. Delegation of this task must be clearly documented and approved by the investigator. All study drug administration will occur under medical supervision.

7. NON-INVESTIGATIONAL PRODUCT

Not applicable

8. METHODS

8.1 Study parameters/endpoints

8.1.1 Main study parameter/endpoint

1. **WP-I**: To determine the mean intraoperative tumor-to-background ratio (TBR) of cRGD-ZW800-1 in patients with oral cancer;
2. **WP-II**: To determine if using FLI can increase the rate of adequate (i.e. >5mm clear) tumor resection margins.

8.1.2 Secondary study parameters/endpoints

1. To assess safety and tolerability of single doses of cRGD-ZW800-1
   Safety and tolerability will be monitored and recorded throughout the study. Safety will be assessed by recording pre-administration events, (S)AEs, MDSFR/SFF data, clinical laboratory parameters, vital signs, ECGs, physical examination and injection site monitoring. Safety data will be reviewed to assure that there is no safety concern that would suggest modification or discontinuation of the protocol. See **10.4**, **6.6.1** and **9.5.**

2. To determine the sensitivity, specificity, positive and negative predictive values of FLI Our unique IOA protocol allows us to determine these parameters much more reliably compared to the world-wide "gold standard". This approach has not been used before in the field of fluorescence-guided surgery.

3. To-determine colocalization of FLI with immunochemistry on pathology slides Standard histopathologic analysis is performed (gold standard) to determine surgical resection margins and growth pattern. Integrin-specific immunohistochemistry will be performed in 3 slides per patient. Next, FLI of paraffin tissue slides without HE-staining is performed using the Odyssey CLx (LICOR, Lincoln, NE) to determine the location of the fluorescent targeting agent at a microscopic level, and to correlate location of fluorescent signal with immunohistochemistry.

4. To determine the percentage of extra tissue resection based on FLI-driven frozen sections.
   For future cost-effectiveness studies, it is relevant to know in how many cases FLI has changed intraoperative management.

5. To determine if FLI significantly increases operation time.
   The total operation time will be determined to assess potential incremental increase in operation time compared to historical data. Although the study protocol may seem elaborate, this is mostly due to the IOA procedure that is standard-of-care at our institution and usually takes about 20-30 minutes.
   Therefore, the only extra intraoperative actions required for this trial are FLI of the wound bed and FLI of the specimen (Fig. 5 and 6 – top row).

In the wound bed analysis, FLI itself will not take much time. When a fluorescent area is found, frozen sectioning is performed. This generally takes about 20 minutes. Because this is performed at the same time as the IOA, it is unlikely to result in a significant increase in operating time.
For the specimen driven analysis, extra operating time of 10 minutes is expected, which is quite modest in a surgical procedure that often takes about 4 hours. If there is fluorescence on the surface but margins are clear on IOA, a frozen section is performed which would take about 20 minutes. Operation time will be recorded and compared to historical data.

6. <u>To determine if lymph node metastases can be identified using FLI</u>
   In many cases, a neck dissection will be performed in patients treated for oral cancer. In these cases, we will perform FLI of the neck dissection specimens ex vivo using the intraoperative camera system, followed by FLI of tissue sections using the Odyssey CLx after they have been processed for pathological analysis. This will allow us to assess if FLI signal is higher in lymph node metastases compared to uninvolved lymph nodes.

8.1.3 Other study parameters

Not applicable

8.2 Randomisation, blinding and treatment allocation

Inclusion of patients will take place in a chronological fashion: the first 7 eligible patients will be part of group A, the next 7 eligible patients will be part of group B.

8.3 Study procedures

<u>SCREENING VISIT (> D-14)</u>

During the screening visit, Investigators will:

1. Obtain signed informed consent from the patient before any study specific procedures are performed;
2. Assign the patient a unique Patient Study Identifier (ID);
3. Determine patient eligibility;
5. Record patient demographic data;
6. Record medical history;
7. Record all concomitant treatments;
8. Record adverse event/toxicity assessment related to the screening procedures;
9. Physical examination and vital signs;
10. ECG;
11. Collection of blood sample for hematology;
12. Collection of blood sample for biochemistry;

TREATMENT ADMINISTRATION DAY (D-1)

During the treatment administration day, the following assessments will be performed:

1. Record adverse event/toxicity assessment between visits and during IMP administration;
2. Record all concomitant treatments added and/or changed;
3. Physical examination (pre-dose);
4. Vital signs and temperature (pre-dose);
5. ECG (pre-dose);
6. Collection of blood sample for hematology (pre-dose); 7. Collection of blood sample for biochemistry (pre-dose);
8. Administration of IMP: cRGD-ZW800-1.
9. Vital signs and temperature at 0.5h, 2h, 4h;
10. ECG at 0.5h; (Peak concentration)
11. MDSFR/SFF measurements (on tumor and on contralateral side) at 0.5h, 2h, 4h;

DAY OF SURGERY (D0)

The surgery is to occur **16-20h** after IMP administration (overnight stay). The following assessments will be performed:

Before surgery:

1. Record adverse event/toxicity assessment;
2. Record all concomitant medications added and/or changed;
3. MDSFR/SFF measurements (on tumor and contralateral side, *while in preoperative holding*);
4. Collection of blood sample for hematology; 5. Collection of blood sample for biochemistry; ***Surgery:***
6. Physical examination and vital signs (monitored during surgery);
7. ECG (monitored during surgery);
8. In-vivo fluorescence imaging (also during neck dissection, if applicable);
9. Resection of tumor.

After surgery:

10. Post-resection fluorescence imaging: in woundbed, on specimen (and in case of neck dissection: also on excised lymph nodes);
11. Intraoperative margin assessment;
12. Histopathology analysis.

POST – SURGICAL HOSPITAL STAY (D0-(D7-D21))

The following assessments will be performed:

1. Record adverse event/toxicity assessment;
2. Record all concomitant medications added and/or changed;
3. Collection of blood sample for hematology (on clinical indication); 4. Collection of blood sample for biochemistry (on clinical indication);
5. Physical examination and vital signs.

DAY OF DISCHARGE (D7-D21)

The following assessments will be performed:

1. Record adverse event/toxicity assessment;
2. Record all concomitant treatments added and/or changed;
3. Collection of blood sample for hematology (on clinical indication); 4. Collection of blood sample for biochemistry (on clinical indication);
5. Physical examination and vital signs.

1ST OUTPATIENT CLINIC VISIT: END OF STUDY (EOS D28-D42)

During this visit, the following assessments will be performed:

1. Record adverse event/toxicity assessment.
2. Discuss histopathology results with the patient.

	Screening			Day of admission + administration cRGD-ZW800-1					Surgery		Postoperative follow-up		
	Intake[1]	Treatment plan[1] + study recruiting[2]	Pre-op intake anesthesiologist						Pre-resection in vivo FLI	Post-resection ex vivo FLI	Post-surgery hospital stay	Day of discharge	1st outpatient clinic visit: EOD
	-	At least -14 days		Pre-dose	0 min	25-35 min	110-130 min	220-260 min	16-20 h	during surgery	In general, 7-21 days		7-14 days after discharge
Informed consent		X[3]		(or) X[3]									
Inclusion and exclusion criteria		X											
Demography	X												
Medical history	X												
Physical examination	X		x	?									
Record concomitant medication	X			X[4]							X[4]		
Routine lab (hem/chem)	X			X					X[5]		X[6]	X[6]	
Vital signs (HR, BP, temp)			X	X		X	X	X	Monitored during surgery		X	X	
12-lead ECG			X	X		X			Monitored during surgery				
Administration cRGD-ZW800-1					X								
MDSFR/SFF measurements[7]						X	X	X	X				
Near-infrared imaging (Quest)									X				
Near-infrared imaging (black box)										X			
Frozen section analysis										X			
AE recording				X	X	X	X	X	X	X	X	X	X

[1] With head & neck surgeon

[2] Patients will be screened for trial eligibility during the weekly multidisciplinary meeting, where all new head and neck cancer patients are discussed.

[3] The patient will be asked if he/she is willing to participate in the study during the second visit to our outpatient clinic. If the patient indicates that he/she may be willing to participate in the trial, the researcher will contact the patient after 5-7 days to provide more detailed information. If the patient consents, they can mail the signed informed consent form, or alternatively sign the form on the day of admission.

[4] After first recording of concomitant medication/treatment, only record if there are changes.

[5] Performed at the start of the operation by anaesthesiologist

[6] Routine lab on clinical indication

[7] MDSFR/SFF (Multi-diameter single-fiber reflectance/ single-fiber fluorescence) spectroscopy measurements. Probe point measurements: 5 times 5 s each.

FU: Follow-up; hem/chem: hematology/chemistry; PK: pharmacokinetics; NIR: near-infrared; AE: adverse events.

8.4 Withdrawal of individual subjects

Subjects can leave the study at any time for any reason if they wish to do so without any consequences. The investigator can decide to withdraw a subject from the study for urgent medical reasons.

8.4.1 Specific criteria for withdrawal

Not applicable

8.5 Replacement of individual subjects after withdrawal

Subjects withdrawing for reasons other than adverse events or any other tolerability issues with the treatment will be replaced.

8.6 Follow-up of subjects withdrawn from treatment

Not applicable

8.7 Premature termination of the study

In case the results of the first group indicate the necessity of a higher dose (too low TBR), the safety data will determine if dose escalation can occur for the second dose group. This decision will be made jointly by the principal investigator, co-investigators and study coordinators (see **6.6.1** and **9.5)**.

Dose escalation can be discontinued in case of an unacceptable tolerability profile (based on the nature, frequency, and intensity of observed AEs). These criteria are guidelines only, and the SC can make an exception, if justified. However, if such an exception is made, the reasons for it should be clearly documented.

The SC will prepare a summary of recommendations derived from the interim analysis to be submitted to the Ethics Committee for approval.

9. SAFETY REPORTING

9.1 Temporary halt for reasons of subject safety

In accordance to section 10, subsection 4, of the WMO, the sponsor will suspend the study if there is sufficient ground that continuation of the study will jeopardise subject health or safety. The sponsor will notify the accredited METC without undue delay of a temporary halt including the reason for such an action. The study will be suspended pending a further positive decision by the accredited METC. The investigator will take care that all subjects are kept informed.

9.2 AEs, SAEs and SUSARs

9.2.1 Adverse events (AEs)

Adverse events are defined as any undesirable experience occurring to a subject during the study, whether or not considered related to [the investigational product / trial procedure/ the experimental intervention]. All adverse events reported

spontaneously by the subject or observed by the investigator or his staff will be recorded.

9.2.2 Serious adverse events (SAEs)

A serious adverse event is any untoward medical occurrence or effect that

- results in death;
- is life threatening (at the time of the event);
- requires hospitalisation or prolongation of existing inpatients' hospitalisation;
- results in persistent or significant disability or incapacity;
- is a congenital anomaly or birth defect; or
- any other important medical event that did not result in any of the outcomes listed above due to medical or surgical intervention but could have been based upon appropriate judgement by the investigator.

An elective hospital admission will not be considered as a serious adverse event.

The investigator will report the SAEs through the web portal *ToetsingOnline* to the accredited METC that approved the protocol, within 7 days of first knowledge for SAEs that result in death or are life threatening followed by a period of maximum of 8 days to complete the initial preliminary report. All other SAEs will be reported within a period of maximum 15 days after the sponsor has first knowledge of the serious adverse events.

9.2.3 Suspected unexpected serious adverse reactions (SUSARs)

Adverse reactions are all untoward and unintended responses to an investigational product related to any dose administered.

Unexpected adverse reactions are SUSARs if the following three conditions are met:

1. the event must be serious (see chapter 8.2.2);
2. there must be a certain degree of probability that the event is a harmful and an undesirable reaction to the medicinal product under investigation, regardless of the administered dose;
3. the adverse reaction must be unexpected, that is to say, the nature and severity of the adverse reaction are not in agreement with the product information as recorded in:

- Summary of Product Characteristics (SPC) for an authorised medicinal product;
- Investigator's Brochure for an unauthorised medicinal product.

The sponsor will report expedited the following SUSARs through the web portal *ToetsingOnline* to the METC:

- SUSARs that have arisen in the clinical trial that was assessed by the METC;

- SUSARs that have arisen in other clinical trials of the same sponsor and with the same medicinal product, and that could have consequences for the safety of the subjects involved in the clinical trial that was assessed by the METC.

The remaining SUSARs are recorded in an overview list (line-listing) that will be submitted once every half year to the METC. This line-listing provides an overview of all SUSARs from the study medicine, accompanied by a brief report highlighting the main points of concern.
The expedited reporting of SUSARs through the web portal Eudravigilance or ToetsingOnline is sufficient as notification to the competent authority.

The sponsor will report expedited all SUSARs to the competent authorities in other Member States, according to the requirements of the Member States.

The expedited reporting will occur not later than 15 days after the sponsor has first knowledge of the adverse reactions. For fatal or life threatening cases the term will be maximal 7 days for a preliminary report with another 8 days for completion of the report.

9.3 Annual safety report

In addition to the expedited reporting of SUSARs, the sponsor will submit, once a year throughout the clinical trial, a safety report to the accredited METC, competent authority, and competent authorities of the concerned Member States.
This safety report consists of:

- a list of all suspected (unexpected or expected) serious adverse reactions, along with an aggregated summary table of all reported serious adverse reactions, ordered by organ system, per study;
- a report concerning the safety of the subjects, consisting of a complete safety analysis and an evaluation of the balance between the efficacy and the harmfulness of the medicine under investigation.

9.4 Follow-up of adverse events

All AEs will be followed until they have abated, or until a stable situation has been reached. Depending on the event, follow up may require additional tests or medical procedures as indicated, and/or referral to the general physician or a medical specialist. SAEs need to be reported till end of study within the Netherlands, as defined in the protocol

9.5 Safety Committee

In order to assure the safe and ethical clinical conduct of the clinical trial all relevant safety data emerging from the study will be reviewed and assessed by the Steering Committee (SC), consisting of the principal investigator and co-investigators; experts in the field of pathology and surgery. The SC will provide oversight and monitoring of the conduct of the clinical trial to ensure the safety of patients. The primary purpose of the SC will be to review the safety data to assure that there is no safety concern that would suggest modification or discontinuation of the protocol. Review will occur prior to proceeding to the next dose cohort. Review will be performed on safety and tolerability

data (i.e. AEs, MDSFR/SFF data, vital signs and available laboratory parameters). This evaluation is especially important when the mean TBR is <2.0 in the first dose group, in which case the SC will determine if it is acceptable to increase the dose, and to what degree.

Dose escalation can always be stopped in case of an unacceptable tolerability profile (based on the nature, frequency, and intensity of observed AEs). These criteria are guidelines only, and the SC can make an exception, if justified. However, if such an exception is made, the reasons for it should be clearly documented.

The SC will prepare a summary of recommendations derived from the interim analysis to be submitted to the ethics committee for approval.

10. STATISTICAL ANALYSIS

Baseline parameters

Continuous demographic variables (age, height, weight, BMI) will be summarized by descriptive statistics (n, mean, SD, median, min, max). When relevant, two-sided 95% confidence intervals will also be computed.

Qualitative demographic characteristics will be summarized by counts and percentages.

Accrual and duration of study

The estimated accrual for this study is 2-4 patients a month. Patient accrual for phase I and phase II are each expected to be completed within 12 months. Because adequate surgical margin rate is the primary endpoint of the Stage II trial, no additional time is required for evaluation. All of the patients registered in the study will be accounted for. The number of patients who were not evaluable, who died or withdrew before treatment began will be specified. Each year, we perform surgery on ~50 patients with oral cancer. Even with an estimated drop-out rate of 15%, inclusion of 14 patients (see **4.4**) per 12 months is highly achievable.

10.1 Primary study parameter(s)

10.1.1 WP-I

<u>The primary endpoint of WP-I is to determine the mean intraoperative TBR</u>.

A lower threshold to differentiate signal from background noise is set at a mean TBR of $\mu 0=2.0$. Based on previous preclinical studies using cRGD-ZW800-1 in vivo[23], and on experience from clinical trials using similar tumor-specific fluorescent targeting agents [25,
26], the $\mu 1$ is set to a mean of 4.0 with a standard deviation σ of 1.5. This standard deviation is wider than what was found in similar studies in other tumors to correct for an anticipated larger variation in integrin expression levels in oral cancers compared to cancer types that were included in other trials. Considering an α of 0.05 and a power of 80%, the sample size (n) is 7 per group, based on the One-Sample 2-sided t-test.

TBR will be calculated by drawing regions of interest (ROI's) to quantify fluorescent signal in tumor and background area (See **5.1.4**). Fluorescence quantified value of tumor and background region will be divided from each other to obtain TBR. TBR will be expressed in descriptive statistics (Mean, SD).

10.1.2 <u>WP-II</u>

<u>The primary endpoint in WP-II is the rate of adequate surgical resection margins.</u>

One group of 7 patients that was analyzed in WP-I with the recommended dose will be included in the single-arm WP-II trial.

Significance level and power:

WP-II tests the null hypothesis that the adequate surgical margins rate is at most 15% versus the alternating hypothesis that the adequate surgical margins rate is at least 40%. An adequate surgical margin is based on the gold standard of histopathologic analysis and is defined as >5mm tumor-free margin from the tumor border, in accordance with the Royal College of Pathologists. The significance level (i.e., the probability of rejecting H0 when it is true) is α=0.05 and the power (i.e., the probability of deciding the regimen is active) is 80%. Based on the A'Hern single stage Phase II design, the expected sample size is 21. The minimal number of successes to indicate that the treatment is effective is 7.

Adequate surgical resection margins will be scored dichotomously (i.e. yes/no).

$$Adequate\ surgical\ resection\ margin\ rate = \frac{no.\ of\ negative\ resection\ margins}{21\ (all\ patients)} \times 100\%$$

10.2 Secondary study parameter(s)

1. <u>To assess safety and tolerability of single doses of cRGD-ZW800-1</u>
   Safety and tolerability will be monitored and recorded throughout the duration of the study. Safety will be assessed by recording pre-administration events, AEs, MDSFR/SFF data, clinical laboratory parameters, vital signs, ECGs, physical examination and injection site monitoring. Parameters will be expressed in descriptive statistics (mean, SD).

2. <u>To determine the sensitivity, specificity, positive and negative predictive values of fluorescence imaging at wound bed and specimen.</u>
   An **event** is defined as a region of interest (ROI) with detectable fluorescence signal. For evaluation of these endpoints, fluorescence signal will be scored in a dichotomous way (yes/no).
   - **Sensitivity** of fluorescence is defined as the events of fluorescence in the surgical wound bed and specimen that has pathological concordance with tumor tissue, divided by all tumor positive samples (including false negative). (TP / (TP+FN))
   - **Specificity** is defined as the events of negative fluorescence in the wound bed and specimen surface and macroscopic intraoperative and microscopic postoperative wide surgical resections, divided by all tumor negative samples (including false positive). TN / (FP+TN)

- **Positive predictive value** of a fluorescence signal is defined as the events of fluorescence signal in the wound bed and specimen surface that were positive for tumor, divided by all events of fluorescence signal (including false positive). TP/(TP+FP)
- **Negative predictive value** of absence of a fluorescence signal, defined as the events of negative fluorescence signal in the wound bed and specimen surface that were negative for tumor, divided by all events of absence of fluorescence signal (including false negative). TN/(TN+FN)

	**Tumor +**	**Tumor -**
**Fluorescence +**	True positive (TP)	False positive (FP)
**Fluorescence -**	False negative (FN)	True negative (TN)

3. To-determine colocalization of FLI with immunochemistry on pathology slides
   For correlation between integrin-specific targeting and immunohistochemistry staining on biopsies and resection specimen, fluorescence will be scored in a dichotomous way (yes/no). Immunohistochemistry will be scored on intensity using a 3-point score: 0=no expression; 1=minimal expression; 2=moderate expression; 3=strong expression.

4. To determine the percentage of extra tissue resection based on FLI-driven frozen sections The percentage of intraoperative change in surgical management based on imaging will be assessed. Intraoperative change in management is defined as the percentage of cases in which a limited extra tissue resection is performed.

$$Change\ in\ surgical\ management = \frac{no.\ of\ changed\ surgical\ plans}{21\ (all\ patients)} \times 100\%$$

5. To determine if FLI significantly increases operation time
   To assess potential incremental increase in operation time, the total operation time will be recorded and compared to historical data. Incremental operation time will be measured in minutes, and will be expressed in descriptive statistics (mean, SD).

6. To determine if lymph node metastases can be identified using FLI
   In cases where a neck dissection is performed, FLI will be performed of the neck dissection specimens in vivo, followed by ex vivo FLI of the tissue sections after pathological analysis. The assessment will consist of a comparison between lymph node metastases to uninvolved lymph nodes in terms of mean fluorescence intensity (MFI), and will be expressed in descriptive statistics (mean, SD).

10.3 Other study parameters

Not applicable

10.4 Interim analysis

The Steering Committee (SC) will evaluate the TBR of the first dose group. In case the results of the first group indicate it necessary (too low TBR), the SC will review the safety data to determine if dose escalation can occur for the second dose group. See **6.6.1** and **9.5**.

11. ETHICAL CONSIDERATIONS

11.1 Regulation statement

The investigator will ensure that this study is conducted in full compliance with the protocol, the principles of the Declaration of Helsinki (www.wma.net), ICH GCP guidelines
(http://www.ich.org/products/guidelines.html), and with the laws and regulations of the country in which the clinical research is conducted.

11.2 Recruitment and consent

Our head and neck surgeon discusses the treatment plan with the patient, usually on the second visit to our outpatient clinic. At this stage, the patient will be asked if he/she is willing to participate in scientific research. This visit with their treating surgeon is emotionally heavy for most patients. In some cases, they may not be open to extended information on research. Therefore, if the patient indicates that he/she may be willing to participate in the trial, the researcher will contact the patient at a later stage to provide more detailed information and gather informed consent.
The patient is always able to opt out of the study without having to give an explanation. This will not interfere with their treatment in any way.

It is the responsibility of the investigator to obtain written informed consent from each individual participating in this study after adequate explanation of the aims, methods, objectives and potential hazards of the study. The investigator must also explain to the subjects that they are completely free to refuse to enter the study or to withdraw from it at any time for any reason.
The Informed Consent and Subject Information will be provided in Dutch. All patients will be informed of the aims of the study, the possible adverse events, the procedures and possible hazards to which he/she will be exposed, and the mechanism of treatment allocation. They will be informed as to the strict confidentiality of their patient data, but that their medical records may be reviewed for trial purposes by authorized individuals other than their treating physician.
It will be emphasized that participation is voluntary and that the patient is allowed to refuse further participation in the protocol whenever he/she wants. This will not prejudice the patient’s subsequent care. Documented informed consent must be obtained for all patients included in the study before they are registered in the study. This must be done in accordance with the national and local regulatory requirements. For European Union member states, the informed consent procedure must conform to the ICH guidelines on Good Clinical Practice. This implies that “the written informed

consent form should be signed and personally dated by the patient or by the patient's legally acceptable representative".

11.3 Objection by minors or incapacitated subjects

Not applicable

11.4 Benefits and risks assessment, group relatedness

The risks of participation for patients in the trial include adverse (hypersensitivity) reactions. These risks are deemed minimal. Nevertheless precautionary measures are in place, including supervised administration by qualified staff and availability of medical treatment to treat hypersensitivity reactions, and these effects are generally well manageable.

Patient burden:

- Consideration to join scientific research at an emotionally heavy time of diagnosis and treatment of cancer;
- Administration of single-dose of fluorescent tracer;
- Additional measurements of vital functions, ECG and laboratory testing after administration;
- Minimal risk of allergic reaction to fluorescent tracer;
- Expected extension of operating time of 10-15 minutes;

Potential patient benefit:

Small chance of higher surgical success rate.

11.5 Compensation for injury

The investigator has a liability insurance which is in accordance with article 7, subsection 6 of the WMO. This insurance provides coverage for damage to research subjects through injury or death caused by the study.

- € 650,000.-- (i.e. six hundred and fifty thousand Euro) for death or injury for each subject who participates in the Research;
- € 5,000,000.-- (i.e. five million Euro) for death or injury for all subjects who participate in the Research;
- € 7,500,000.-- (i.e. seven million five hundred thousand Euro) for the total damage incurred by the organization for all damage disclosed by scientific research for the Sponsor as 'verrichter' in the meaning of said Act in each year of insurance coverage.

The insurance applies to the damage that becomes apparent during the study or within 4 years after the end of the study.

11.6 Incentives

Not applicable

12. ADMINISTRATIVE ASPECTS, MONITORING AND PUBLICATION

12.1 Handling and storage of data and documents

For the imaging outcome measurements, data will be collected from imaging software incorporated into the various fluorescent imaging systems. Information on histopathologic outcome and immunohistochemistry will be retrieved from the medical record files. If applicable to evaluate AEs, medical history and baseline CTCAE characteristics will also be retrieved from the medical record.
Data will be stored in a secured trial database and managed by the Clinical Trial Center of the Erasmus MC.

12.2 Monitoring and Quality Assurance

This study is determined to be of intermediate risk, refer to *K6. Monitoringplan* for more information.

- This study will be conducted according to applicable Standard Operating Procedures (SOPs).
- The parallel tagging procedure with subsequent intraoperative assessment of the specimen with the pathologist both have to be performed by all surgeons. This procedure is the current gold standard for assessment of tumor-free margins.

Data will be collected using an eCRF (electronic case report form) designed for this study. According to ICH guidelines for Good Clinical Practice, the monitoring team must check the specific CRF entries against the source documents, in a subset of patients, as specified in the study specific monitoring plan. The Informed Consent Form will include a statement by which the patient allows the Sponsor’s duly authorized personnel, the ethics review committee (IRB/ERC) or similar or expert committee, and the regulatory authorities to have direct access to original medical records which support the data on the CRFs (e.g. patient’s medical file). This personnel, bound by professional secrecy, must maintain the confidentiality of all personal identity or personal medical information, according to confidentiality and personal data protection rules and in compliance with all applicable privacy laws, rules, and regulations.

12.3 Amendments

Any change to a protocol has to be considered as an amendment.

A ‘substantial amendment’ is defined as an amendment to the terms of the METC application, or to the protocol or any other supporting documentation, that is likely to affect to a significant degree:

- the safety or physical or mental integrity of the subjects of the trial;
- the scientific value of the trial;
- the conduct or management of the trial; or
- the quality or safety of any intervention used in the trial.

All substantial amendments will be notified to the METC and to the competent authority. Non-substantial amendments will not be notified to the accredited METC and the competent authority, but will be recorded and filed by the investigator/sponsor.

12.4 Annual progress report

The sponsor/investigator will submit a summary of the progress of the trial to the accredited METC once a year. Information will be provided on the date of inclusion of the first subject, numbers of subjects included and numbers of subjects that have completed the trial, serious adverse events/ serious adverse reactions, other problems, and amendments.

12.5 Temporary halt and (prematurely) end of study report

The sponsor will notify the accredited METC and the competent authority of the end of the study within a period of 90 days. The end of the study is defined as the last patient's last visit.

The sponsor will notify the METC immediately of a temporary halt of the study, including the reason of such an action.
In case the study is ended prematurely, the sponsor will notify the accredited METC and the competent authority within 15 days, including the reasons for the premature termination.

Within one year after the end of the study, the investigator/sponsor will submit a final study report with the results of the study, including any publications/abstracts of the study, to the accredited METC and the Competent Authority.

12.6 Public disclosure and publication policy

In accordance with standard editorial and ethical practice, the results of the study will be published, if applicable.

13. STRUCTURED RISK ANALYSIS

13.1 Potential issues of concern

a. Level of knowledge about mechanism of action cRGD-ZW800-1 consists of a fluorophore conjugated to a small peptide targeting integrins associated with neoangiogenesis. Nonclinical pharmacology studies have demonstrated cRGDZW800-1's efficacy in binding to multiple tumor types.

b. Previous exposure of human beings with the test product(s) and/or products with a similar biological mechanism

Several clinical studies with imaging or anticancer agents have successfully utilized the overexpression of integrins on tumors, without relevant adverse events. Preclinical toxicity studies,a Phase 1 study in healthy volunteers and a Phase II study in patients with colorectal tumors did not raise any safety concerns. Toxicity associated with cRGD-

ZW800-1 use should therefore be minimal. In addition, the fluorophore ZW800-1 has been administered to healthy volunteers and doses up to 5.0 mg did not show signs of acute or chronic toxicity or any other clinically relevant event. However, in another study, the use of ZW800-1 was evaluated in patients with a kidney insufficiency who were scheduled for a kidney transplantation (immunocompromised patients). The aim of the study was to evaluate whether kidney perfusion could be assessed with fluorescence. For the study, both the kidney donor and kidney receiver received a bolus of 1.0 mg ZW800-1 during surgery. A total of 4 patients were included (2 kidney donors and 2 kidney receivers), where both kidney receivers had a rejection of the transplanted kidney. In one patient the rejection resolved with medication and in one patient the transplanted kidney was removed within 24 hours. Due to the fact that an association with ZW800-1 could not be excluded, the study was prematurely terminated. The kidney donors in the study did not experience any adverse events.

c. Can the primary or secondary mechanism be induced in animals and/or in *ex-vivo* human cell material?

Both in vitro and in vivo studies with human cells and animal models allowed to confirm that cRGD-ZW800-1 recognizes malignant cells that show overexpression of integrins. Moreover, the renal clearance pattern was shown in several animal models. Clearance patterns are important, as hepatic clearance may contaminate intestines and result in non-specific background fluorescence.

d. Selectivity of the mechanism to target tissue in animals and/or human beings

Expression of integrins can be found on tumor cells and tumor-associated vascular endothelium and correlates with neoangiogenesis. Overexpression is found on almost all solid tumors, including breast, colorectal, pancreas, brain, lung, and other cancers. cRGD-ZW800-1 may therefore act as a generic tracer for a broad variety of solid tumors. However, neoangiogenesis can also be found in non-malignant processes, including inflammation. It is expected that the sensitivity of cRGD-ZW800-1 is very high, with a moderate specificity.

e. Analysis of potential effect

Based on the results of preclinical pharmacology studies performed in tumor-bearing mice with fluorescence imaging, it is anticipated that a human dose of 5 to 50 µg/kg should be appropriate for clinical use. It is proposed to initiate the clinical trial at a dose of 5 µg/kg and not to exceed 50 µg/kg. Considering the no observable adverse event level in rats of at least 5.0 mg/kg, doses up to 80 µg/kg are considered safe starting doses for human studies.

f. Pharmacokinetic considerations

The starting dose of cRGD-ZW800-1, the escalation steps, and the time to perform surgery after dosing are based upon the assumption that sufficient amount of cRGD-ZW800-1 will have reached the site of action, while unbound tracers have cleared from the background. This assumption is supported by preclinical and clinical data currently available. The sampling schedule will allow population-based PK modeling

g. Study population

The study population will be patients with oral squamous cell carcinoma who are scheduled to undergo resection of the tumor. Females of childbearing potential may participate in the trial, but only when they agree to use highly an effective form of contraception in the cases where the child bearing potential is retained post-surgery. Patients who are immunocompromised are excluded, as well as patients with renal insufficiency or who have a kidney transplantation in the medical history.

h. Interaction with other products

Not applicable

i. Predictability of effect

Participation in this study could result in the intraoperative detection of more malignant lesions than would have been detected with standard practices. The surgeon will resect additional fluorescence-identified lesions, when the presence of tumor is confirmed through frozen section analysis and macroscopic intraoperative assessment.

j. Can effects be managed?

The risks of participation for patients in the trial include adverse (hypersensitivity) reactions. These risks are deemed minimal. Nevertheless, precautionary measures are in place, including supervised administration by qualified staff and availability of medical treatment to treat hypersensitivity reactions, and these effects are generally well manageable.

13.2 Synthesis cRGD-ZW800-1 has no known biological actions other than binding to integrins. This translated into an unremarkable toxicity profile in animals and an adverse clinically relevant adverse event free Phase 1 study. This warrants investigation of cRGD-ZW800-1 in patients employing a cautious dose-escalation approach in a dedicated hospital.

14. REFERENCES

1. Smits, R.W., et al., *Resection margins in oral cancer surgery: Room for improvement.* Head Neck, 2015.
2. Dillon, J.K., et al., *How does the close surgical margin impact recurrence and survival when treating oral squamous cell carcinoma?* J Oral Maxillofac Surg, 2015. **73**(6): p. 1182-8.
3. van Lanschot, C.G.F., et al., *Relocation of inadequate resection margins in the wound bed during oral cavity oncological surgery: A feasibility study.* Head Neck, 2019. **41**(7): p. 2159-2166.
4. Frangioni, J.V., *New technologies for human cancer imaging.* J Clin Oncol, 2008. **26**(24): p. 4012-21.
5. Keereweer, S., et al., *Optical image-guided surgery--where do we stand?* Mol Imaging Biol, 2011. **13**(2): p. 199-207.

6. Keereweer, S., et al., *Optical imaging of oral squamous cell carcinoma and cervical lymph node metastasis.* Head Neck, 2012. **34**(7): p. 1002-8.
7. Keereweer, S., et al., *Dual wavelength tumor targeting for detection of hypopharyngeal cancer using near-infrared optical imaging in an animal model.* Int J Cancer, 2012. **131**(7): p. 1633-40.
8. Keereweer, S., et al., *Targeting integrins and enhanced permeability and retention (EPR) effect for optical imaging of oral cancer.* J Surg Oncol, 2012. **105**(7): p. 714-8.
9. Keereweer, S., et al., *Detection of oral squamous cell carcinoma and cervical lymph node metastasis using activatable near-infrared fluorescence agents.* Archives of otolaryngology--head & neck surgery, 2011. **137**(6): p. 609-15.
10. Choi, H.S., et al., *Synthesis and in vivo fate of zwitterionic near-infrared fluorophores.* Angew Chem Int Ed Engl, 2011. **50**(28): p. 6258-63.
11. Naumov, G.N., L.A. Akslen, and J. Folkman, *Role of angiogenesis in human tumor dormancy: animal models of the angiogenic switch.* Cell Cycle, 2006. **5**(16): p. 1779-87.
12. Schittenhelm, J., et al., *Comparing the expression of integrins alphavbeta3, alphavbeta5, alphavbeta6, alphavbeta8, fibronectin and fibrinogen in human brain metastases and their corresponding primary tumors.* Int J Clin Exp Pathol, 2013. **6**(12): p. 2719-32.
13. Beer, A.J., et al., *Positron emission tomography using [18F]Galacto-RGD identifies the level of integrin alpha(v)beta3 expression in man.* Clin Cancer Res, 2006. **12**(13): p. 3942-9.
14. Beer, A.J., et al., *Comparison of integrin alphaVbeta3 expression and glucose metabolism in primary and metastatic lesions in cancer patients: a PET study using 18F-galacto-RGD and 18F-FDG.* J Nucl Med, 2008. **49**(1): p. 22-9.
15. Beer, A.J., et al., *Patterns of alphavbeta3 expression in primary and metastatic human breast cancer as shown by 18F-Galacto-RGD PET.* J Nucl Med, 2008. **49**(2): p. 255-9.
16. Kenny, L.M., et al., *Phase I trial of the positron-emitting Arg-Gly-Asp (RGD) peptide radioligand 18F-AH111585 in breast cancer patients.* J Nucl Med, 2008. **49**(6): p. 879-86.
17. Axelsson, R., et al., *An open-label, multicenter, phase 2a study to assess the feasibility of imaging metastases in late-stage cancer patients with the alpha v beta 3-selective angiogenesis imaging agent 99mTc-NC100692.* Acta Radiol, 2010. **51**(1): p. 40-6.
18. Bach-Gansmo, T., T.V. Bogsrud, and A. Skretting, *Integrin scintimammography using a dedicated breast imaging, solid-state gamma-camera and (99m)Tc-labelled NC100692.* Clin Physiol Funct Imaging, 2008. **28**(4): p. 235-9.
19. Doss, M., et al., *Biodistribution and radiation dosimetry of the integrin marker 18F-RGD-K5 determined from whole-body PET/CT in monkeys and humans.* J Nucl Med, 2012. **53**(5): p. 787-95.
20. Manegold, C., et al., *Randomized phase II study of three doses of the integrin inhibitor cilengitide versus docetaxel as second-line treatment for patients with advanced non-small-cell lung cancer.* Invest New Drugs, 2013. **31**(1): p. 175-82.

21. Verbeek, F.P., et al., *Near-infrared fluorescence imaging of both colorectal cancer and ureters using a low-dose integrin targeted probe.* Ann Surg Oncol, 2014. **21 Suppl 4**: p. S528-37.
22. Choi, H.S., et al., *Targeted zwitterionic near-infrared fluorophores for improved optical imaging.* Nat Biotechnol, 2013. **31**(2): p. 148-53.
23. Handgraaf, H.J.M., et al., *Real-time near-infrared fluorescence imaging using cRGD-ZW800-1 for intraoperative visualization of multiple cancer types.* Oncotarget, 2017. **8**(13): p. 21054-21066.
24. de Valk, K.S., et al., *First-in-Human Assessment of cRGD-ZW800-1, a Zwitterionic, Integrin-Targeted, Near-Infrared Fluorescent Peptide in Colon Carcinoma.* Clin Cancer Res, 2020.
25. Gutowski, M., et al., *SGM-101: An innovative near-infrared dye-antibody conjugate that targets CEA for fluorescence-guided surgery.* Surg Oncol, 2017. **26**(2): p. 153-162.
26. Boogerd, L.S.F., et al., *Safety and effectiveness of SGM-101, a fluorescent antibody targeting carcinoembryonic antigen, for intraoperative detection of colorectal cancer: a dose-escalation pilot study.* Lancet Gastroenterol Hepatol, 2018. **3**(3): p. 181-191.
