# Supplementary material for: Near-infrared fluorescence imaging-guided surgery using cRGD-ZW800 to improve surgical resection margins in oral cancer: a phase I/II feasibility trial
Source: Nat Commun. 2026 May 22;17:7341. doi: 10.1038/s41467-026-73554-7 (PMC13402695; doi:10.1038/s41467-026-73554-7)
Supplement: Supplementary file 2 — Description of Additional Supplementary Files [file 41467_2026_73554_MOESM2_ESM.pdf]

Description of Additional Supplementary Files:

Supplementary Movie 1: *In vivo* fluorescence imaging. Representative video (2x speed) of real-time *in vivo* near-infrared fluorescence imaging before and during resection in a patient with a tumor in the lateral tongue
