# Supplementary material for: Near-infrared fluorescence imaging-guided surgery using cRGD-ZW800 to improve surgical resection margins in oral cancer: a phase I/II feasibility trial
Source: Nat Commun. 2026 May 22;17:7341. doi: 10.1038/s41467-026-73554-7 (PMC13402695; doi:10.1038/s41467-026-73554-7)
Supplement: Supplementary file 4 — Reporting Summary [file 41467_2026_73554_MOESM4_ESM.pdf]

nature portfolio

Corresponding author(s): Dr. S. (Stijn) Keereweer
Drs. B.E. (Bo) Zweedijk

Last updated by author(s): Aug 22, 2025

Reporting Summary

Nature Portfolio wishes to improve the reproducibility of the work that we publish. This form provides structure for consistency and transparency in reporting. For further information on Nature Portfolio policies, see our Editorial Policies and the Editorial Policy Checklist.

Statistics

For all statistical analyses, confirm that the following items are present in the figure legend, table legend, main text, or Methods section.

n/a	Confirmed	
☐	☒	The exact sample size (*n*) for each experimental group/condition, given as a discrete number and unit of measurement
☐	☒	A statement on whether measurements were taken from distinct samples or whether the same sample was measured repeatedly
☐	☒	The statistical test(s) used AND whether they are one- or two-sided<br>*Only common tests should be described solely by name; describe more complex techniques in the Methods section.*
☐	☒	A description of all covariates tested
☐	☒	A description of any assumptions or corrections, such as tests of normality and adjustment for multiple comparisons
☐	☒	A full description of the statistical parameters including central tendency (e.g. means) or other basic estimates (e.g. regression coefficient) AND variation (e.g. standard deviation) or associated estimates of uncertainty (e.g. confidence intervals)
☐	☒	For null hypothesis testing, the test statistic (e.g. *F*, *t*, *r*) with confidence intervals, effect sizes, degrees of freedom and *P* value noted<br>*Give P values as exact values whenever suitable.*
☒	☐	For Bayesian analysis, information on the choice of priors and Markov chain Monte Carlo settings
☒	☐	For hierarchical and complex designs, identification of the appropriate level for tests and full reporting of outcomes
☒	☐	Estimates of effect sizes (e.g. Cohen's *d*, Pearson's *r*), indicating how they were calculated

Our web collection on statistics for biologists contains articles on many of the points above.

Software and code

Policy information about availability of computer code

Data collection	Data collection was performed using Alea Data Management, version 18.8.
Data analysis	Data analysis was conducted using RStudio version 4.3.2, employing custom R scripts. The following R packages were used: dplyr, tableone, ggplot2, tidyr, readr, tibble, stringr, lubridate, caret, tidyverse, shiny, rmarkdown, knitr, magrittr, data.table, Rcpp, devtools, R6, shinydashboard, leaflet, readxl, psych, ggpubr, grid, rstatix, and lattice. The complete R code used for all analyses is publicly available in the GitHub repository: https://github.com/BoKosterZweedijk/R-code-Guided-by-Light-studie.git.

For manuscripts utilizing custom algorithms or software that are central to the research but not yet described in published literature, software must be made available to editors and reviewers. We strongly encourage code deposition in a community repository (e.g. GitHub). See the Nature Portfolio guidelines for submitting code & software for further information.

Data

Policy information about availability of data

All manuscripts must include a data availability statement. This statement should provide the following information, where applicable:

- Accession codes, unique identifiers, or web links for publicly available datasets
- A description of any restrictions on data availability
- For clinical datasets or third party data, please ensure that the statement adheres to our policy

The fluorescence imaging data that support the findings of this study, including those presented in Figures 2, Figure 4, and Supplementary Figure 4, have been processed and compiled into an Excel file ("Data Source File"), which has been publicly deposited and is available in the Figshare repository at https://

doi.org/10.6084/m9.figshare.29763728.
Clinical data regarding histopathological status and occurrence of complications, as presented in Table 1 and Supplementary Table 1, were extracted from patient medical records. Due to privacy regulations and ethical restrictions, these sensitive patient-level data cannot be publicly shared.

All imaging, safety, clinical, and laboratory data (limited to non-identifiable information) can be obtained from the corresponding author upon request (S. Keereweer, s.keereweer@erasmusmc.nl). Data will be retained for at least 15 years in accordance with Dutch regulations. Additional data supporting the findings of this study are included in the Article, Supplementary Materials, or Source Data file. The Source Data File and the study protocol are publicly accessible via the following Figshare repository: https://doi.org/10.6084/m9.figshare.29763728

Research involving human participants, their data, or biological material

Policy information about studies with human participants or human data. See also policy information about sex, gender (identity/presentation), and sexual orientation and race, ethnicity and racism.

Reporting on sex and gender	In this study, no distinction was made between sex and gender. Only sex, as recorded in the patients' medical records, was used; gender identity was not assessed or discussed with the patients. The informed consent permitted the use of personal data extracted from medical records. No sex-stratified analyses (male vs. female) were performed, because this was not relevant to the aims of the study. The purpose of this research was to evaluate the use of fluorescence imaging in oral cancer patients, a technical, procedure-focused outcome, rather than to investigate patient-related biological or identity-related differences. Therefore, sex- or gender-based comparisons were not considered meaningful for the study objectives.
Reporting on race, ethnicity, or other socially relevant groupings	Race, ethnicity, or other socially relevant groupings were neither collected nor used in this study. The primary objective was to evaluate the effectiveness of fluorescence imaging at the individual patient level, specifically to determine whether it provided an adequate surgical margin and to assess tumor-to-background contrast. Since the analysis focused on these technical parameters without comparing patients based on demographic or social categories, race and ethnicity were not considered relevant variables. Moreover, given the nature of the study, inclusion of such variables was not deemed necessary, and their absence is unlikely to have impacted the outcomes.
Population characteristics	All included patients were diagnosed with the same cancer type: oral squamous cell carcinoma of the oral cavity. Relevant population characteristics collected for the study included sex (as recorded in the medical record), age, and exact tumor location. Patients who had received chemotherapy, radiotherapy, or surgery in the tumor region prior to participation were excluded, in order to maintain a homogeneous study population. These characteristics were collected to provide clinical context, but no additional covariates were analyzed, as the study focused on evaluating the performance of fluorescence imaging on an individual patient basis.
Recruitment	Eligible patients were identified during multidisciplinary team (MDT) meetings, based on predefined inclusion criteria (biopsy proven oral squamous cell carcinoma of the oral cavity, for which surgical intervention was needed). Once a suitable candidate was identified during the meeting, the patient was approached by the surgeon during a consultation at the Head and Neck Oncology outpatient clinic at the Erasmus MC. The surgeon briefly discussed potential participation, without providing study details, and, if the patient expressed interest, the study investigator was contacted to provide further information and obtain informed consent. Since all patients with this tumor type were systematically discussed in the MDT meeting, there is no apparent risk of selection bias.
Ethics oversight	The study was approved by the local Ethics Review Committee (METC Erasmus MC) and conducted in full compliance with the principles of the Declaration of Helsinki of 1975, the ICH GCP guidelines, and the laws and regulations of the Netherlands.

Note that full information on the approval of the study protocol must also be provided in the manuscript.

Field-specific reporting

Please select the one below that is the best fit for your research. If you are not sure, read the appropriate sections before making your selection.

[x] Life sciences [ ] Behavioural & social sciences [ ] Ecological, evolutionary & environmental sciences

For a reference copy of the document with all sections, see nature.com/documents/nr-reporting-summary-flat.pdf

Life sciences study design

All studies must disclose on these points even when the disclosure is negative.

Sample size	Sample size calculations were performed for two distinct endpoints.<br>I. Dose-finding and feasibility:<br>Three dose levels of the fluorescent tracer were tested. A one-sample, two-sided t-test (α = 0.05, power = 80%) was used to detect a difference in mean tumor-to-background ratio (μ_0 = 2.0 vs. μ_1 = 4.0, σ = 1.5), resulting in a required sample size of 7 patients per dose group. The optimal dose was selected based on feasibility and imaging performance.<br><br>II. Correlation between fluorescence imaging and surgical margins:<br>All patients with a detectable fluorescence signal (n=31) were included in the margin analysis. Since detectability of all doses could not be predicted beforehand, the sample size calculation was applied only to the optimal dose group, as identified in part I. Using the A'Hern single-stage Phase II design, a sample size of 21 patients was required to test whether the adequate margin rate was ≥40% (vs. ≤15%), with α = 0.05 and 80% power. A minimum of 7 patients with adequate margins was needed to consider the imaging technique effective.

Data exclusions	In our analyses, one patient (patient 11) was excluded from the final analysis due to a malfunction of the fluorescence imaging device during the procedure. As the study outcomes rely on data obtained from this imaging system, it was not possible to include this patient in the analysis.
Replication	For the spectroscopy data, measurements were performed in triplicate, and the average value was used for analysis. If any measurement visibly deviated substantially from the others or appeared incorrect during data collection, an additional measurement was taken, and the outlier was excluded. Regarding the ex vivo tumor-to-background ratio (TBR) measurements, regions of interest (ROIs) were initially delineated and values extracted by one researcher. Subsequently, two other researchers independently repeated the ROI delineation and value extraction to verify consistency. In cases of discrepancy, the researchers discussed the differences and reached a consensus on the most accurate delineation.
Randomization	This study focused on dose-finding and feasibility, where randomization is not applicable. Patients were sequentially assigned to dose cohorts based on predefined escalation schemes. Therefore, no randomization procedures were performed or required.
Blinding	The amount of adequate and inadequate surgical margins was initially determined solely through the standard intraoperative pathological assessment by the operating surgeon and pathologist. Our study introduced an additional technique, fluorescence imaging, to assist in identifying areas of interest. Importantly, the surgeon and the pathologist performed their routine frozen section analysis without knowledge of the fluorescence findings, ensuring they were blinded to the fluorescent signal during the initial margin evaluation. Only after this initial assessment, the fluorescent spots were revealed to the pathologist, allowing for additional tissue sampling if deemed necessary. This allowed us to determine the effect of fluorescence alone on the amount of adequate and inadequate resection margins.

Reporting for specific materials, systems and methods

We require information from authors about some types of materials, experimental systems and methods used in many studies. Here, indicate whether each material, system or method listed is relevant to your study. If you are not sure if a list item applies to your research, read the appropriate section before selecting a response.

Materials & experimental systems

n/a	Involved in the study
☒	☐ Antibodies
☒	☐ Eukaryotic cell lines
☒	☐ Palaeontology and archaeology
☒	☐ Animals and other organisms
☐	☒ Clinical data
☒	☐ Dual use research of concern
☒	☐ Plants

Methods

n/a	Involved in the study
☒	☐ ChIP-seq
☒	☐ Flow cytometry
☒	☐ MRI-based neuroimaging

Clinical data

Policy information about clinical studies

All manuscripts should comply with the ICMJE guidelines for publication of clinical research and a completed CONSORT checklist must be included with all submissions.

Clinical trial registration	The study is registered in the European Clinical Trials Database (EudraCT 2019-003416-30) and ClinicalTrials.gov (NCT04191460).
Study protocol	The full study protocol has been shared with the Senior Editor via email and will be made publicly available through the Figshare repository at https://doi.org/10.6084/m9.figshare.2976372.
Data collection	Data were collected at the Department of Otorhinolaryngology - Head and Neck Surgery at Erasmus Medical Center between July 2022 and April 2025. Following informed consent, patients underwent screening including blood sampling and electrocardiogram (ECG) to confirm eligibility. On the day of tracer administration and again on the day of surgery, repeat blood tests and ECGs were performed to monitor for any physiological changes. Fluorescence data were collected via spectroscopy at multiple time points post-injection (30 minutes, 2 hours, 4 hours, and 18 hours). Intraoperative fluorescence imaging was performed using a dedicated surgical camera system and these findings were documented.<br><br>After tumor resection, the specimen was imaged using a closed-field fluorescence system. Any fluorescent areas identified were noted for potential relevance. The specimen was then transferred to pathology for standard intraoperative evaluation. Standard frozen sections were taken as clinically indicated, and their location, number, and results (adequate vs. inadequate margins) were recorded. If fluorescence hotspots had been identified using fluorescence imaging, additional frozen sections could be taken at those locations, and this was similarly documented. Any additional resections performed intraoperatively were also recorded.<br><br>Postoperatively, patients were monitored weekly for complications, and a final complication check was performed 30 days after surgery. After 30 days, the follow-up was complete.<br><br>All data were collected and managed using ALEA Data Management, version 18.8.
Outcomes	Primary outcomes:<br>1. To determine the recommended dose of cRGD-ZW800-1 yielding the highest tumor-to-background ratio (TBR): TBR was measured

in vivo using Multi-diameter single-fiber reflectance and single-fiber fluorescence (MDSFR/SFF) spectroscopy at multiple standardized time points (30 minutes, 2 hours, 4 hours, and 18 hours) after tracer injection, and ex vivo using a closed-flied imaging system (before intraoperative pathological assessment). The mean TBR was calculated both in vivo and ex vivo, and a threshold of >2.0 was defined as indicative of adequate signal intensity.
2. To assess whether intraoperative fluorescence imaging with cRGD-ZW800-1 can reliably detect all inadequate resection margins during oral cancer surgery and influence the decision-making.

Secondary outcomes:
1. To assess safety and tolerability of single doses of cRGD-ZW800-1: Assessed through clinical monitoring, ECGs, and laboratory tests (including blood panels) at screening, preoperative, and intraoperative time points, as well as through documentation of any adverse events up to 30 days postoperatively.
2. To assess the efficacy of fluorescence imaging (FI): Sensitivity, specificity, positive predictive value (PPV), and negative predictive value (NPV) were calculated by comparing the presence of fluorescence signals with corresponding histopathological findings (adequate vs. inadequate margins). This was done on both lesion-level and patient-level.
3. To correlate fluorescence signal with integrin expression: Assessed through immunohistochemical (IHC) staining for αvβ6 integrin on formalin-fixed, paraffin-embedded tumor sections. After final histopathologic analysis, fluorescence imaging of the tissue slides was performed using a flatbed scanner to correlate fluorescent signal with IHC and H&E histopathology.
4. To determine the rate of intraoperative changes in surgical management based on fluorescence imaging: All additional frozen sections or resections performed based on fluorescence signal (i.e., after initial standard assessment) were recorded, including their anatomical location and histopathological outcome.
5. To determine the incremental operative time: Assessed by comparing time stamps of surgical start and end times, as well as the duration specifically related to fluorescence imaging procedures, as documented in the surgical log.

Plants

Seed stocks

No plant specimens were collected

Novel plant genotypes

No plant specimens were collected

Authentication

No plant specimens were collected
