# Supplementary material for: Near-infrared fluorescence imaging-guided surgery using cRGD-ZW800 to improve surgical resection margins in oral cancer: a phase I/II feasibility trial
Source: Nat Commun. 2026 May 22;17:7341. doi: 10.1038/s41467-026-73554-7 (PMC13402695; doi:10.1038/s41467-026-73554-7)
Supplement: Supplementary file 5 — Transparent Peer Review file [file 41467_2026_73554_MOESM5_ESM.pdf]

nature portfolio

Peer Review File

Near-infrared fluorescence imaging-guided surgery using cRGD-ZW800 to improve surgical resection margins in oral cancer: a phase I/II feasibility trial.

Corresponding Author: Dr Stijn Keereweer

This file contains all reviewer reports in order by version, followed by all author rebuttals in order by version.

Version 1:

Reviewer comments:

Reviewer #1

(Remarks to the Author)
This manuscript presented a fluorescence-guided navigation approach using a tumor-specific dye, cRGD-ZW800-1, and systematically evaluated its safety, feasibility, and performance in patients with oral squamous cell carcinoma. Interestingly, the authors found that near-infrared fluorescence imaging with cRGD-ZW800-1 successfully altered surgical plans, thereby avoiding the need of adjuvant radiotherapy. Although the performance of cRGD-ZW800-1 has already been evaluated in patients with multiple tumor types, this work provided important clinical results for facilitating the clinical translation of the probe and improving the prognosis of patients with oral cancers. However, the clinical data presented here are not adequately analyzed or clearly presented. I therefore have the following comments that should be well addressed before I can recommend this manuscript for publication：
1. Although the authors have explained the rationale for choosing the sample size, I am still concerned about the small sample size (n = 3) for the low dose group (0.01 mg/kg). Notably, the tumor sites in these three patients are distinct and the variation for the TBRs in these patients is very large (2.72 ± 2.27, Relative Standard Deviation = 83.5%) . I am wondering about whether the TBR values correlate with the tumor sites.
2. The authors claimed that they successfully quantified the in vivo pharmacokinetics of the tracer using MDSFR/SFF spectroscopy, for the first time. Please provide the spectra, linear relationship, and quantification limit for this methodology in details.
3. The authors claimed that fluorescence imaging exhibited a significantly greater tumor detection rate than standard IOA (p = 0.003), but the data supporting this statistical analysis is not presented in the main text or SI file.
4. The authors found salivary glands (n = 14) and other tissues (gingiva and bone) showed intense fluorescence, while multiple micro-satellite lesions (0.05 to 0.3 mm in diameter) and the second primary tumors (diameter of 1.4 mm) were not fluorescent. Undoubtedly, these off-target fluorescence would lower the specificity and sensitivity of the cRGD-ZW800-1 based fluorescence imaging for detecting oral cancers. Please check whether the sensitivity (95.8%) and specificity (80%) are correct. Although the salivary gland tissue was easily distinguishable by both surgeon and pathologist, this is not relevant to the performance of fluorescence imaging.
5. The FDA-approved ICG has been widely used in the intraoperative detection of oral cancers. I believe that it would be better to give a discussion on the advantages of cRGD-ZW800-1 over ICG.
6. The mean ex vivo TBRs (Fig. 4) are much higher than the in vivo TBRs (Fig. 2). Please give explanations. Besides, the mean ex vivo TBRs for dose cohorts 0.01, 0.025 and 0.05 mg/kg were 4.52 ± 1.60, 5.73 ± 2.75 and 5.76 ± 2.03, respectively, which seems to be not in line with the Fig. 4. Please check. Also, these values cannot support the claim “All tested doses were well-tolerated with no drug-related adverse events and achieved ex vivo tumor-to-background ratios exceeding 5.0”.
7. The authors believed that the consistent fluorescence in healthy gingiva did not impose an issue for gingival tumor delineation. Please provide strong evidence and calculate the TBR as well.
8. In Fig. 3J, the non-tumoral tissue also showed intense fluorescence. Please explain it.
9. In the Figs. 2&4 and Supplementary Fig. 3, the bars are not explained and statistical analysis is not presented. Besides, these are several data points in the Supplementary Fig. 3 without any description.
10. To ensure the reproducibility of this work, the imaging parameters for in vivo and ex vivo fluorescence imaging should be detailedly described, such as the excitation wavelengths, emission filters, exposure times, excitation power, and distance between the light source and tissues.
11. In the Methodology section, the description “Patients with synchronous biopsy-proven active malignancies or previous

surgery, chemo- or radiotherapy to the oral cavity were excluded” is not consistent with “Thirty-one patients with biopsy-proven OSCC scheduled for standard-of-care surgery were included in this study” (in the Result section).
12. The advantages of near-infrared fluorescence imaging over the label-free techniques (such as autofluorescence imaging) in detecting oral cancers are not introduced in the background. I believe some references can provide solid support regarding its capability to overcome the interference from blood and its high tissue-penetrating property, such as Nat. Photon., 2024, 18(12): 1254-1262; Nat. Mater. 2021, 20, 1571-1578; Nat. Nanotechnol. 2023, 18, 1195-1204; CCS Chemistry, 2022, 4(2): 476-486.

Reviewer #2

(Remarks to the Author)
This is a well written, interesting paper on the added value of fluorescence imaging (FI) on surgical resection margins in SCC of the oral cavity. This technique appears to be promising and I think there is a realistic chance that it will supplement surgical management in the future. I also note prior well cited publications on the theme in Nature Communications, and so this article aligns with the journal's existing topical areas of interest. Noteworthy results include a novel fluorescence imaging agent, showing good tolerability in this patient sample (n=31) and potentially good tumour specificity. Also, a proportion (5/31) of patients in whom surgical approach and outcome was altered (favourably) due to FI findings, obviating the need for adjuvant radiotherapy. There are similar studies in the literature, but not at a significantly more mature stage of investigation (tending to be similar in terms of single centre, double-digit patient numbers), and so this study would contribute to the existing body of evidence. Clarification on some items would be beneficial with regards consideration for publication (and for inclusion in any future systematic review or meta analysis).

Items for clarification:

Abstract:
1. Unless otherwise instructed by the journal, the abstract might be divided into background, method, results, conclusion.

Introduction:
2. Explicit definition of the study type is suggested (single centre, prospective, single arm... etc). In the introduction (line 89), the paper implies that it is a feasibility study. In the methods/ statistical analysis sub-section (line 230), it implies that it is a phase II study (elsewhere, the authors allude to their previous phase I/II study). Is it a feasibility study or other category of study/ trial? If a feasibility study, do the authors conclude that a larger multi-centre study/ RCT is feasible and desirable? If so, have the authors drawn inferences on the foreseeable resources, costs, regulatory, time-scale, organisational, and training implications, and/ or necessary patient numbers, risks and obstacles this might involve? Noting that 31 patients were recruited over an approximately 3 year interval.
3. Consider more explicit definition of primary and secondary objectives/ metrics. In the method section (line 134) and in the protocol (supplementary material), the authors suggest that an objective is to "determine whether FI could IMPROVE the rate of adequate tumour resections..." In the introduction section (line 107): "...we hypothesise that this approach ... reduce inadequate resection margins and ... enhance patient outcomes". Accepting that the study quantified a proportion of patients whose surgery was modified in order to avoid what would otherwise have been an inadequate resection, without a control arm, the objective might be reworded in terms of absolute efficacy metrics, rather than indicating comparative performance against standard of care (i.e. according to phase II level objectives). Consider stating quantifiable outcome measures.
4. Were potentially applicable study frameworks or reporting guidelines considered e.g. IDEAL in surgical innovation or STARD in diagnostic accuracy studies?
5. line 86: "... has shown tumour-specific imaging without toxicity in healthy volunteers and colon cancer patients." Consider alternative wording in relation to healthy volunteers.

Methods:
6. Were the patients recruited consecutively (to address selection bias)? Have the authors considered reporting the study against criteria by which it might be considered for inclusion in a systematic review/ meta-analysis (e.g. CONSORT extension for feasibility studies or STARD)?
7. How many operators/ years experience? Was any training needed for the technique?
8. Is the fluorescent contrast agent an investigational medicinal product? If so, would this be governed/ overseen by a particular domestic regulatory body?

Stats:
9. Did the authors consider including ROC/ AUC calculations?
10. Line 226. "... this analysis tests the null hypothesis that the adequate surgical margin rate is at most 15% versus the alternating hypothesis that the adequate margin rate is at least 40%..." How were these percentages decided? Was it based on the quoted range of inadequate margin rates in the literature of 67-85%? If so, did the authors select the highest quoted inadequate margin rate to test the null hypothesis? Do the authors have an estimate of their own local centre's margin adequacy rates, as this might better contextualise these particular results (if available).

Results:
11. With regards safety (Results, line 243). Were these classified according to Clavien Dindo or Common Terminology for AEs (CTCAE)? No AE were recorded in relation to the administered agent itself, which is encouraging. There were 21 AE/SAEs in a series of 31 patients. Is this higher than expected? Did FI impact the post-surgical complications? For example, did the AE/SAEs predominate in cases where further resection was indicated on the basis of FI analysis? Wider

resections might impact other outcome measures e.g. length of stay, swallow etc. Was there a perceived trade-off, or was this felt to be beyond the scope of the study?
12. Table 1. Study characteristics (p.20). In the column "inadequate margin found with FI" (Yes/No). In every case with a stated result in this column (24/32), the result is a 'YES'. In the other 8/32 cases, the result is recorded as a '-'. What does this mean? Is '-' equivalent to a 'NO', or does it mean that margin assessment with FI was not performed in these cases? If it is a 'NO', it may add clarity to state this (or otherwise explain), so as not to imply that FI was 'positive' in every case where it was employed. Presumably there were cases where FI was 'negative', as non-zero true and false negative rates are quoted in Table 2 (supplementary material). The authors state that all positive margins were detected with FI, but document 1 false negative in Table 2. Please clarify.

Discussion:
13. line 409: "...FI altered decision making in specific cases by identifying inadequate margins..." Consider stating/ repeating the number/ proportion of applicable cases.
14. Line 418: "... intraoperative FI was validated through objective in vivo quantification..." Perhaps this conclusion would be pending external centre reproducibility of findings/ multi-centre study?
15. Line 431: Typo "... for wat least..."
16. Line 466: Limitations statement is quite brief.

Thanks to the authors for this engaging read, and to the journal for the invitation to review.

Reviewer #3

(Remarks to the Author)
The goals of this study were to study the safety and feasibility as well as the performance of fluorescence imaging (FI) to improve surgical resection margins in oral cancer. The paper is hard to follow due to the presentation of the results.

One of the objectives of the study was to identify the optimal dose of the agent, however in the end the analysis included patients studied at all 3 doses (7 studied at 0.05, 3 studied at 0.01 and 21 studied at 0.025). Since the dose 0.025 was the dose deemed to have optimal performance and the power calculations and protocol defined the rate of adequate surgical resection margins based on the patients evaluated at the optimal dose, for consistency and generalizability to future studies, the primary endpoint should be assessed only among patients evaluated at the the optimal dose. Furthermore, the decision rule was based on 21 patients and this calculation should be highlighted in the paper. Table 1 is based on 32 patients further confusing the results.

It is not clear why IOA was considered the gold standard (pg. 13, 475), shouldn't the postoperative margin status be the gold standard for the calculations of sensitivity and specificity in Suppl Table 2? Are calculations in that table at the lesion level or patient level? Why can you have Intraoperative margin status Positive, but No inadequate margin found with IOA (pt ids 27 and 30)? Why patients that were Clear on Intraoperative margin status do not have a status for Inadequate margin found with FI? How do you establish specificity in that case? More details on how Suppl Table 2 was obtained should be provided.

The paper (pg. 10, 344-347) states that "Importantly, in four patients, the tissue samples for assessment were mistakenly taken from areas immediately adjacent to, rather than directly from, the fluorescent spots identified by FI. This sampling error led to false-negative IOA results and missed opportunities for additional resections." however this contradicts the statement (pg. 6, 169-170) that "To prevent bias, both surgeon and pathologist were blinded to ex vivo FI findings. Fluorescent signals at specimen margins were only disclosed after standard IOA was completed." Please clarify.

It is not clear how the 4 patients whose "inadequate margins could not be addressed intraoperatively due to difficulties in localizing the fluorescent signal for targeted frozen section sampling" were counted towards the primary endpoint and towards the accuracy metrics. Please clarify in Table 1 and Suppl Table 2.

The paper (pg. 10, 336-337) states that in 9 out of 31 patients, FI revealed inadequate margins that were not identified by standard IOA, however the paper should also state that only some of these patients had additional resection performed.

Given that the decision to perform additional resection was still the surgeon's decision, we can't entirely claim that the improvements in the rate of adequate resection margins are due to FI-guided.

Reviewer #4

(Remarks to the Author)
This is a very interesting trial addressing feasibility, safety, and utility of fluorescence optical imaging for surgery of oral cavity carcinoma. The authors report the results of a prospective study on 31 patients, with findings ranging from optimal dose identification to impact on intraoperative decision making and histopathological assessments. Overall, the study was well designed, conducted, and reported.
I list here some points to be discussed in the context of this peer review, and that could potentially elicit revision of the manuscript:

MAJOR POINTS:
- "ZW800-1, which binds specifically to integrins (αvβ1, αVβ3, αvβ5, αvβ6, αvβ8, α5β1, α8β1 and αIIbβ3)" -> does it mean that the the drug used in the study binds several other integrins apart from αvβ6 (which was studied through IHC)? How this fact impact on the biological and clinical value of this method? Is it a potential source of inaccuracy of the method used herein?
- "In the remaining three cases, fluorescence was detected in non-suspicious anatomical structures: the mandibular bone marrow at the resection plane (n=2) and lingual salivary gland tissue (n=1). None of these areas were deemed clinically suspicious, and the corresponding margins on the tumor specimen were histologically tumor-negative." -> the fact that minor salivary glands and bone marrow are fluorescent is a potential source of bias, particularly if one thinks to a broader application of this technique. To limit such limitations, authors allude to the fact that clinical appearance of these tissues would be different to connective tissues were tumor foci could be located. Still, I find it very arbitrary and subjective, and thus prone to bias. This aspect mandates more elaboration in the Discussion in my opinion.
- Definition of inadequate margin: the study is based on the assumption that margin below 5 mm is inadequate. Although authors often report separate data on positive vs close margins, they are taken altogether in terms of statistics. This assumption does not rely on sound evidence and should be critically discussed. There is evidence suggesting that the average threshold to define close margin is actually lower (10.1001/jamaoto.2017.0548, 10.1001/jamaoto.2016.4238). Also, studies suggest that such threshold could change according to the pattern of invasion of the tumor, thus taking into account biological heterogeneity in the context of squamous cell carcinoma (10.1002/hed.26956). Moreover, comparing fluorescence imaging with intraoperative frozen section-based assessment of margins is partially biased by the fact that frozen section is not capable of detecting close margins, but only positive margins. Clinical evidence of the comparison is evident and clear, but I would find it fairer if authors clearly state that the methods that was used for comparison can't identify most cases that they defined with inadequate margins.
- "high sensitivity (95.8%) and specificity (80%)" -> definition and number of false positive, false negative, true positive and true negative should be clearly reported.
- In the paragraph "Effect of FI on intraoperative decision making" it is difficult to get an overall measure of impact on decision making. The authors should report the number (and proportion) of cases with decision-making actually impacted by the use of fluorescence imaging, before deepening into details of specific cases.
- "Importantly, in four patients, the tissue samples for assessment were mistakenly taken from areas immediately adjacent to, rather than directly from, the fluorescent spots identified by FI. This sampling error led to false-negative IOA results and missed opportunities for additional resections. These cases highlight an important limitation of relying solely on frozen section analysis." -> these sentences appear to carry an important message, namely that fluorescence imaging can drive sampling for frozen section. I believe this message deserves more emphasis.
- "In general, the deep margin is rightly considered more important than the mucosal margin" -> this is untrue. The mucosal margin is as prognostically important as the deep margin. The latter might be more difficult to be adequately delineated, but this does not relate to importance.
- "As such, this study likely underestimates the true added value of FI" -> this sentence is at risk of being simplistic; whether the study under- or over-estimates the added value of fluorescence imaging very much depends on how outcomes are measured. As stated above, if one considers inadequate margin everything below 5 mm and compares fluorescence imaging with frozen section (that do not reliably detect close margins), the added value of fluorescence imaging could actually be over-estimated. On the other hand, I appreciate the reasoning of the authors and agree on arguments posed to conclude for a potential underestimation. That's why I believe the sentence risks being simplistic, there are several factors to be considered.

MINOR POINTS:
- "Referencing the initial mucosal outline defined by the surgeon prior to imaging, the fluorescent areas never suggested inadequate margins, supporting the surgeon’s accurate assessment–subsequently confirmed by histopathology" -> reading literally this sentence would imply that fluorescence imaging did not positively impact on mucosal margin delineation (i.e., only on the deep margin), is that the case?
- "(Serious) adverse events were monitored continuously" -> "(Serious)" makes it potentially confusing and misunderstanding. Consider rewording ("Adverse events were monitored continuously" probably expresses what was done).
- "Study proceedings did not meaningfully interfere with the standard of care, nor resulted in significantly prolonged surgery time" -> are there objective data to support this statement?
- Table 1 -> please, specify the Edition of TNM used.
- Figure 3 -> lateral border tumors seem the easiest to be processed; could authors show (maybe as supplement) the appearance of a specimen from more complex resections?

Version 2:

Reviewer comments:

Reviewer #1

(Remarks to the Author)
I have no comments

Reviewer #2

(Remarks to the Author)

Many thanks to the authors for their responses to my comments. I am satisfied that they are addressed, and that the article meets the standards for publication.

Reviewer #3

(Remarks to the Author)
Although the authors attempted to address the reviewers' comments, there remains some confusion regarding the definition of diagnostic metrics and the handling of certain patients in the computation of these metrics.

The Statistical Analysis section still does not include a proper definition of sensitivity, specificity, PPV, and NPV with respect to what was the gold standard used for these calculations. In the response letter, the authors state that "ultimate evaluation of the efficacy (including diagnostic performance) of FI was based on the definitive histopathological assessment after surgery" however this should be clearly spelled out in the statistical section. Some details were provided in the legend of the Suppl Table 2 but these details do not clarify what the nominators and denominators in the calculations are.

Furthermore, the authors state that the calculations are at lesion-level but this is not explained anywhere and confidence intervals should be included to account for within-patient correlations.

Regarding the 4 patients for whom tissue was mistakenly taken from areas adjacent to the fluorescent spots identified by FI (point 5, R3), the authors respond that these patients were not considered false positives, but do not explain how they were counted towards the diagnostics metrics.

Should the diagnostics metrics for standard IOA be included in Suppl. Table 2?

McNemar's test was added to the analysis but it is not listed in the Statistical Analysis section.

The statement on pg. 10, line 420 on power should be removed given that these is likely post-hoc power calculation that is not meaningful.

The references in the response letter pointing to the line numbers do not match the actual line numbers in the paper.

Reviewer #4

(Remarks to the Author)
I congratulate with authors on the very comprehensive and punctual way they responded to reviewers' comment. Points I've raised have been properly addressed. I've no further comments other than congratulating with authors for this valuable piece of clinical science.

Version 3:

Reviewer comments:

Reviewer #3

(Remarks to the Author)
The revised manuscript attempted to address previous concerns raised after their first resubmission but managed to introduce more confusion and in fact brought to surface some serious methodological flaws with their approach. Although histopathology is stated to be the gold standard, it is only performed on tissue deemed suspicious by IOA and/or FI, biasing the estimates of sensitivity, specificity, PPV, and NPV. Fluorescence-positive but clinically "non-suspicious" tissue is selectively excluded from false positives, while missed lesions are excluded from false negatives, further inflating performance metrics. The paper mixes clinical utility arguments with diagnostic accuracy claims which are not supported given the exclusions and the decisions made in the definitions of FN, FP, TN, TP. To properly assess diagnostic accuracy, all fluorescent and non-fluorescent findings must be counted consistently against gold standard (histopathology in this case), without exclusions based on clinical relevance and this can't be evaluated in this study the way it was conducted. The paper may have clinical utility, but it requires a major revision to frame it as such and to remove the conflation with diagnostic accuracy throughout the paper.

Major methodological flaws

Sensitivity requires knowledge of all true positives that were missed. Because only suspicious margins were sent to pathology (pg. 7, line 260), undetected tumor at non-sampled margins cannot be identified, making false negatives unobservable by design.

Missed second primary tumors and microsatellite lesions are explicitly excluded from diagnostic performance analysis (pg. 10 400-407), even though these represent false negatives of FI. Excluding them biases sensitivity. If histopathology shows tumor and FI did not detect it, by definition this represents a false negative; see pg. 7 271-272: "In addition, non-fluorescent areas that were subsequently found to have an inadequate margin were considered false negative".

The authors exclude fluorescence-positive tissue that was deemed clinically non-suspicious from false positives (pg. 9 351-354, pg. 10 384-387), despite histology confirming absence of tumor. This directly violates the definition of specificity and inflates NPV. Only two bone marrow cases are counted as false positives, while fluorescence in other non-suspicious structures (e.g., lingual salivary gland tissue) is explicitly not counted as false positive. There is no methodological justification for this selective exclusion.

There were 4 patients for whom tissue was sampled adjacent to the fluorescent signal rather than at the signal itself who had clear IOA but for whom histopathology showed inadequate margins. Classifying these cases as “true positives for FI” is a major flaw. Assuming that FI was correct just because the final margin was inadequate elsewhere assumes spatial concordance that was not histologically verified. The gold standard should be applied at the same site as the index test. If adjacency in itself is accepted as confirmation of tumor, then how are we to know that FI didn’t miss tumor elsewhere (because that adjacent tissue was by the fluorescent spots but was not fluorescent itself), and that these cases are not in fact false negatives rather than true positives.

The authors claim diagnostic accuracy was assessed at the patient level, but Supplementary Table 2 contains 33 TP/TN/FP/FN classifications for 31 patients, indicating that multiple observations per patient were included. This directly contradicts the authors’ response raising doubts on the quality of this revision.

The revision now includes sensitivity and specificity separately for “positive” and “close” margins, despite margin status being a three-category outcome (clear / close / positive). Sensitivity and specificity are defined for binary variables and the paper does not comment on collapsing categories which would result in combinations that are not meaningful, such as clear and positive tissues called as non-disease.

On one hand the authors claim that FI is “evaluated based on its added clinical value in supporting real-time surgical decision-making, rather than as a standalone diagnostic tool” yet in other places FI is presented as a standalone diagnostic tool (even the tables should refer to IOA+FI rather than FI alone): “Had FI alone guided resection decisions” (pg. 11 430).

Version 4:

Reviewer comments:

Reviewer #3

(Remarks to the Author)
The revision now is much improved and clearly separates the decision accuracy analysis vs patient-level clinical impact analysis making the approach methodologically sound.

Minor comments:

The presentation of the Results section could benefit of restructuring (possibly with separate subheadings) to clearly separate between the two analyses as now the structure is a bit dense.

There appears to be a typo: on pg. 10 line 415, 15 close margins and 8 positive margins (also listed in Table 3) are noted however pg. 13 line 547 refers to a single positive margin.

Include the specifics of the software that was used for the analyses.

Open Access This Peer Review File is licensed under a Creative Commons Attribution 4.0 International License, which permits use, sharing, adaptation, distribution and reproduction in any medium or format, as long as you give appropriate credit to the original author(s) and the source, provide a link to the Creative Commons license, and indicate if changes were made.
In cases where reviewers are anonymous, credit should be given to 'Anonymous Referee' and the source.
The images or other third party material in this Peer Review File are included in the article's Creative Commons license, unless indicated otherwise in a credit line to the material. If material is not included in the article's Creative Commons license and your intended use is not permitted by statutory regulation or exceeds the permitted use, you will need to obtain permission directly from the copyright holder.
To view a copy of this license, visit https://creativecommons.org/licenses/by/4.0/

Reviewer 1

This manuscript presented a fluorescence-guided navigation approach using a tumor-specific dye, cRGD-ZW800-1, and systematically evaluated its safety, feasibility, and performance in patients with oral squamous cell carcinoma. Interestingly, the authors found that near-infrared fluorescence imaging with cRGD-ZW800-1 successfully altered surgical plans, thereby avoiding the need of adjuvant radiotherapy. Although the performance of cRGD-ZW800-1 has already been evaluated in patients with multiple tumor types, this work provided important clinical results for facilitating the clinical translation of the probe and improving the prognosis of patients with oral cancers. However, the clinical data presented here are not adequately analyzed or clearly presented. I therefore have the following comments that should be well addressed before I can recommend this manuscript for publication:

1. Although the authors have explained the rationale for choosing the sample size, I am still concerned about the small sample size (n = 3) for the low dose group (0.01 mg/kg). Notably, the tumor sites in these three patients are distinct and the variation for the TBRs in these patients is very large (2.72 ± 2.27, Relative Standard Deviation = 83.5%) . I am wondering about whether the TBR values correlate with the tumor sites.

We thank the reviewer for this insightful comment. In response to the reviewer's inquiry regarding a potential correlation between TBR and tumor site, we note that within the low-dose cohort, the tumors are located in the lateral tongue (n=1) and the floor of the mouth (n=2). Upon examining the TBR values for these subsites across the entire cohort, the mean TBRs are comparable: 2.42 for the lateral tongue (n=15) and 2.69 for the floor of the mouth (n=6). This suggests that tumor site does not exert a systematic influence on TBR and thus will not have accounted for the observed variation at this dose level.

We believe that this high variation is attributable to other factors. In response to the reviewer's concern, we re-examined all raw and corrected data from this cohort. This analysis revealed that the observed variation is driven predominantly by a single measurement from one patient. Accordingly, we would like to clarify that the observed deviation arises from the intrinsic difficulty of this measurement. This datapoint involves the lowest administered dose in combination with the longest injection-to-interrogation interval, which together make it the most challenging condition in the entire dataset. Under these circumstances, both tumor and background fluorescence signals are close to the detection threshold, and the relative contribution of noise is at the highest. Because the TBR is calculated as a ratio of two low-intensity measurements, even small fluctuations in either measurement can lead to an amplified apparent variation.

As described previously (Hoy et al., J Biomed Opt., 2013), the correction for tissue optical properties involves a two-step sequence consisting of two reflectance acquisitions followed by one fluorescence acquisition. The underlying model assumes that the light-collection geometry remains constant across these acquisitions. In practice, the small fluctuations that were seen in these specific measurements are most likely caused by small movements of the probe or interrogated tissue. This effect becomes more pronounced at very low intrinsic fluorescence levels.

Importantly, during data collection we can usually recognize inadequate measurements caused by tissue or operator movement. In this particular case, no such irregularity was apparent at the time of acquisition, and therefore we had no clear methodological basis for excluding the datapoint. Nevertheless, given the combination of extremely low signal levels and the sensitivity of the optical-property correction to small geometric changes, this remains the most plausible explanation for the elevated deviation seen in this measurement.

2. The authors claimed that they successfully quantified the in vivo pharmacokinetics of the tracer using MDSFR/SFF spectroscopy, for the first time. Please provide the spectra, linear relationship, and quantification limit for this methodology in details.

To address this important point, we have added Supplementary Figure 1. This figure shows an example of a SFF emission spectrum collected from (a) the centre of tumor and (b) contralateral normal tissue 18h after tracer administration, that has been corrected for the tissue optical properties (m_a and m'_s) at the excitation wavelength and in the within the emission band of the fluorophore. Where the corrected data (red circles) is the fitted with a linear combination of in-vivo basis spectrum for c-RGD-800ZW-1 (orange dashed line, fitted as a skewed gaussian) determined from a large group of measurements with the tracer present, and the basis spectrum for background autofluorescence, (green line) which is a combination of tissue autofluorescence and background fluorescence in the spectroscopy system.

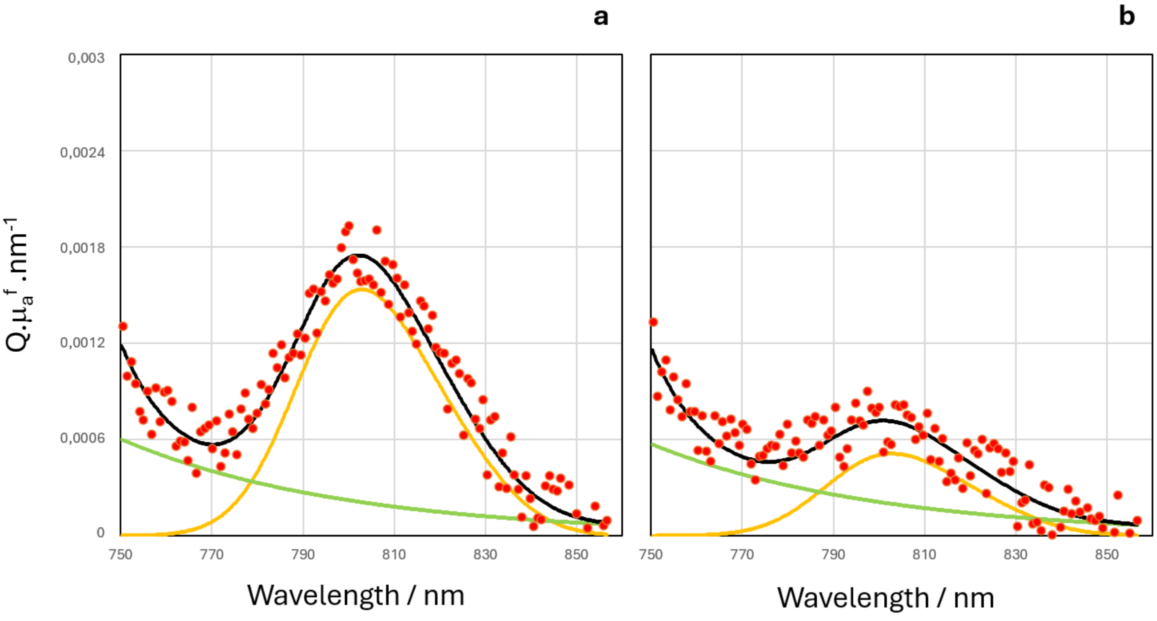

In the figure, the corrected data (red circles) are fitted with a linear combination of the in vivo basis spectrum for cRGD-800ZW-1 (orange dashed line), derived from a large dataset of tracer-containing measurements, and the basis spectrum for tissue autofluorescence. The green spectrum represents a fluorescence background that is the combined signal from tissue autofluorescence and the autofluorescence originating from the optical fibers and other components of the spectroscopy system. This approach enables quantitative recovery of fluorescence signals.

The correction for tissue optical properties and the quantitative recovery methodology are based on previously validated empirical models using in silico simulations and liquid phantoms (Kanick et al., Biomed Opt. Express, 2021; Gamm et al., Biomed Opt. Express, 2014). The linearity of the recovered quantitative fluorescence signal ($Q.\mu_a f$) has been demonstrated in phantom studies (Hoy et al., J Biomed Opt., 2013). At the detection limit of $Q.\mu_a f$, the estimated concentration of ZW800-1 (with $\varepsilon = 1.7 \times 10^5$ M^{-1} cm^{-1} and Q = 0.19) corresponds to approximately 1.2 nM.

To provide full methodological detail, we have now dedicated a separate paragraph in the Supplementary Materials describing the spectral correction, fitting procedures, and linearity assessment. This section is referenced in the main manuscript in the Methods section (page 6, line 223-224):

"Details of the spectral correction, linear fitting procedure, and quantification limits are provided in Supplementary Figure 1."

3. The authors claimed that fluorescence imaging exhibited a significantly greater tumor detection rate than standard IOA (p = 0.003), but the data supporting this statistical analysis is not presented in the main text or SI file.

We have now added the quantitative data and statistical test results underlying this statement. Specifically, standard intraoperative assessment identified 14 of 23 inadequate resection margins, corresponding to a detection rate of 61%, whereas FI detected all 23 inadequate margins (100%). This difference was statistically significant, as determined by the Exact binomial McNemar's test ($p = 0.004$). These data demonstrate that FI enables a more comprehensive identification of inadequate margins compared to IOA. Furthermore, on request of one of the other reviewers, we added the same data on the detection rate of the optimal dose group separately.

Initially, we applied the Mann-Whitney U test to compare the detection rate of FI compared to standard IOA, as it is commonly used for comparing non-normally distributed data between two independent groups. However, after consulting with a statistical expert, we recognized that our data were paired, as both tests were performed on the same patients. Therefore, we revised our analysis to use the Exact binomial McNemar's test, which is specifically designed for paired binary data, and more appropriately evaluates differences in diagnostic outcomes between the two methods. This adjustment resulted in a change of the initial p-value (p = 0.004 instead of p = 0.003), but the outcome remains statistically significant, and this method ensures a more accurate statistical approach for our paired data.

To incorporate this clarification, we have revised the Results section (page 9, lines 362-370) as follows (this is in combination with feedback from the other reviewer):

"All close (1-5 mm) and positive (<1 mm) resection margins exhibited fluorescence intensity-peaks (example shown in Figure 3H). In the full cohort (n=31), 15 close and 8 positive margins were all successfully detected using FI, whereas nine inadequate margins were missed by standard IOA by surgeon and pathologist (three positive and six close margins). Focusing on the optimal-dose cohort (n = 21), all 14 inadequate margins (6 positive and 8 close margins) were again detected by FI, while IOA missed seven cases (3 positive, 4 close). Consequently, FI demonstrated a significant higher inadequate margin detection rate compared to standard IOA for both the full cohort (100% vs. 61%, Exact binomial McNemar's test, p = 0.004) and the optimal-dose cohort (100% vs. 50%, Exact binomial McNemar's test, p = 0.008)."

This revision ensures that the supporting data and statistical rationale for the reported significance are now fully described within the main text, thereby improving the rigor and transparency of the manuscript.

4. The authors found salivary glands (n = 14) and other tissues (gingiva and bone) showed intense fluorescence, while multiple micro-satellite lesions (0.05 to 0.3 mm in diameter) and the second primary tumors (diameter of 1.4 mm) were not fluorescent. Undoubtedly, these off-target fluorescence would lower the specificity and sensitivity of the cRGD-ZW800-1 based fluorescence imaging for detecting oral cancers. Please check whether the sensitivity (95.8%) and specificity (80%) are correct. Although the salivary gland tissue was easily distinguishable by both surgeon and pathologist, this is not relevant to the performance of fluorescence imaging.

We carefully considered the underlying rationale for choosing a specificity of 80%, which we believe is clinically meaningful given the intended use of fluorescence imaging. As emphasized in our study, we do not view fluorescence imaging as a standalone diagnostic test but rather as an intraoperative tool to assist the surgeon in real-time decision-making. Therefore, we believe the inclusion of salivary glands in the specificity calculation should be guided by clinical relevance. In a typical surgical setting, salivary glands are not considered suspicious for tumor tissue and, regardless of their fluorescence intensity,

would not undergo further investigation (such as fresh frozen sectioning). Thus, the presence of fluorescence in these tissues does not alter the clinical management, as they would not be flagged as suspicious and would not affect surgical decisions. For this reason, these tissues were not included in the initial specificity calculation. Moreover, to assess the true clinical value of fluorescence imaging in the operating room, it is more appropriate to calculate specificity excluding the salivary glands, as their inclusion would not reflect the real-world clinical decision-making process.

We have revised the manuscript to further clarify the methodology concerning the specificity calculation and the intended role of fluorescence imaging in our study. Specifically, we have added the following text to the Methods section (page 7, line 261-267):

"Diagnostic performance was determined based on sensitivity, specificity, negative predictive value (NPV), positive-predictive value (PPV) and accuracy in detecting inadequate resection margins. As FI is not intended as a standalone diagnostic tool, but rather is an adjunct to guide real-time surgical decision making, only clinically relevant tissues were included in this analysis. This approach ensures that only these clinically relevant tissues, which could potentially influence surgical management, were included, providing a more accurate reflection of FI's performance in real-world clinical setting."

Regarding the reviewer's concern about the non-fluorescent second primary tumor (1.4 mm in diameter), we would like to clarify that this lesion did, in fact, exhibit a clear fluorescent signal ex vivo, as shown in Supplementary Figure 2 (top row). The absence of an in vivo fluorescence signal was due to the fact that the current state-of-the art intraoperative camera systems are not yet capable of detecting these very low signal intensities in the clinical setting. However, as this second primary tumor was fluorescent during intraoperative ex vivo imaging and was resected with an adequate surgical margin, this did not have any clinically relevant consequences.

We also respectfully maintain that the distinguishability of salivary gland fluorescence is clinically relevant for evaluating the utility of FI. Intraoperative imaging performance depends not only on probe specificity but also on the ability of the surgical team to correctly interpret fluorescence signals in real time. This integrated assessment aligns with the clinical feasibility objectives of the present study and is consistent with previously published intraoperative fluorescence imaging studies that likewise included salivary glands in the performance analysis.

5. The FDA-approved ICG has been widely used in the intraoperative detection of oral cancers. I believe that it would be better to give a discussion on the advantages of cRGD-ZW800-1 over ICG.

We agree that a comparison between cRGD-ZW800-1 and the clinically used fluorophore ICG provides important context for understanding the advantages of our imaging approach. Accordingly, we have expanded the Discussion section (page 13, lines 520-525) to include the following sentence:

"*While the FDA-approved fluorophore indocyanine green (ICG) has been widely studied for intraoperative detection of oral cancers, its non-specific accumulation in surrounding tissues often leads to a decrease in tumor contrast, limiting tumor delineation. In contrast, cRGD-ZW800-1 provides receptor-mediated tumor targeting and lower background fluorescence, resulting in higher tumor-to-background ratios under intraoperative conditions[20,47].*"

This addition highlights the key benefits of cRGD-ZW800-1 over ICG in the context of targeted intraoperative fluorescence imaging.

6. The mean ex vivo TBRs (Fig. 4) are much higher than the in vivo TBRs (Fig. 2). Please give explanations. Besides, the mean ex vivo TBRs for dose cohorts 0.01, 0.025 and 0.05 mg/kg were 4.52 ± 1.60, 5.73 ± 2.75 and 5.76 ± 2.03, respectively, which seems to be not in line with the Fig. 4.

Please check. Also, these values cannot support the claim "All tested doses were well-tolerated with no drug-related adverse events and achieved ex vivo tumor-to-background ratios exceeding 5.0".

First, as we noted earlier in the manuscript, lower in vivo TBRs compared to ex vivo measurements are expected and well-documented in the field. Intraoperative factors such as surrounding light and camera positioning typically reduce in vivo contrast. This phenomenon is not specific to our study or tracer and has been discussed in prior publications[1].

Second, regarding the discrepancy between the mean TBR values and Figure 4, the figure presents median TBRs. While mean values are commonly used in the main text to describe results, median values are conventionally shown in boxplots to represent the distribution of data. The horizontal line within each box represents the median, rather than the mean, because the median provides a more robust measure of central tendency that is less influenced by outliers and skewed distributions. This ensures a clearer representation of the typical value in the dataset. For this reason, and to maintain methodological accuracy, we have retained the median representation in Figure 4. We hope the reviewer agrees that this approach provides the most reliable and transparent visualization of the data.

Additionally, after re-examining the data and the R code, we discovered a small error in the initial analysis. Once we corrected this issue, we observed slight changes in the figure, particularly in the 0.05 mg/kg dose boxplot. These changes now align with the data we have, and we felt it was important to inform the reviewer of this correction. After making this adjustment, we thoroughly reviewed the entire code for any further issues and can confirm that no additional errors were found.

Finally, we agree that the original statement regarding ex vivo TBRs exceeding 5.0 is not accurate. Based on the measured data, all tested doses achieved a mean ex vivo TBR of at least 4.5. We have corrected the sentence in the Abstract section (page 2, lines 47-48) to reflect this:

"*All tested doses were well-tolerated with no drug-related adverse events and achieved mean ex vivo tumor-to-background ratios exceeding 4.5."*

These revisions clarify the TBR presentation and ensure accurate reporting of the dose-dependent imaging performance.

7. The authors believed that the consistent fluorescence in healthy gingiva did not impose an issue for gingival tumor delineation. Please provide strong evidence and calculate the TBR as well.

As noted earlier in one of the previous comments, reduced in vivo contrast can occur due to suboptimal camera angles and limited accessibility of the primary lesion. In the case of gingival tumors, these were located at the retromolar trigone or alveolar process of the maxilla and mandible. Due to the posterior positioning of these tumors within the oral cavity, it is challenging to position the camera perpendicularly to the lesion, which contributes to the reduced in vivo contrast observed. Therefore, the conclusion regarding reliable gingival tumor delineation will be based on ex vivo specimen images and spectroscopy measurements.

Ex vivo analysis and spectroscopy revealed a mean mucosal TBR of 2.04 (SD 0.79) in gingival tumor specimens. Spectroscopic measurements confirmed that all individual patients with gingival tumors achieved adequate mucosal TBRs, with the lowest measured TBR being 2.1. According to the literature, a contrast of 1.5 is generally accepted as the threshold above which tumor tissue can be reliably distinguished from surrounding mucosa using fluorescence imaging[2]. Importantly, we did not observe any case of an inadequate mucosal margin.

These findings provide strong evidence that, despite the presence of fluorescence in healthy gingiva, tumor delineation remains reliable and clinically feasible. We have added the following sentence to the

Results section (page 8, lines 323-332), this includes an adjustment on request of one of the other reviewers:

"Referencing the initial mucosal outline defined by the surgeon prior to imaging, the fluorescent areas never suggested inadequate margins, supporting the surgeon's accurate assessment–subsequently confirmed by histopathology. This indicates that mucosal delineation can be achieved with well-defined boundaries and high contrast using FI, resulting in adequate identification of all mucosal margins. Although a consistent fluorescence signal was observed in healthy gingiva (Supplementary Figure 2), ex vivo FI revealed a mean mucosal TBR of 2.04 (SD 0.79) in gingival tumor specimens. Furthermore, in vivo spectroscopy measurements confirmed that all individual patients with gingival tumors achieved adequate mucosal TBRs, with the lowest measured TBR being 2.1, indicating that physiological fluorescence in healthy gingival did not impose an issue for mucosal tumor delineation."

8. In Fig. 3J, the non-tumoral tissue also showed intense fluorescence. Please explain it.

The fluorescence observed in the non-tumoral tissue (Figure 3J) is localized at a region of desmoplasia (as confirmed by our pathologist), a fibrotic stromal reaction commonly seen in and around tumors (see the attached HE slide below; the desmoplastic region surrounding the tumor is indicated with a black line). Immunohistochemistry staining of this specific slide indeed revealed integrin expression in this desmoplastic region, although at substantially lower levels than in the tumor itself. Similar findings have been reported in other tumor types, such as colon carcinoma and colorectal liver metastases, where desmoplastic regions also show elevated integrin expression relative to normal tissue[3].

Importantly, this low-level signal was not detected during imaging of the fresh specimen or bread loaves. Therefore, this fluorescence did not interfere with margin assessment or affect clinical decision-making in our study.

To clarify this, we have added the following sentence to the Results section (page 11, line 458-462):

"Some mild fluorescence was also observed outside the tumor boundaries (Figure 3, image J), originating from a desmoplastic reaction at the tumor border. This region exhibited αvβ6 expression, although at substantially lower levels compared to tumor. Importantly, this low-level signal was not detectable during imaging of the fresh specimen or bread loaves and did not interfere with intraoperative margin assessment."

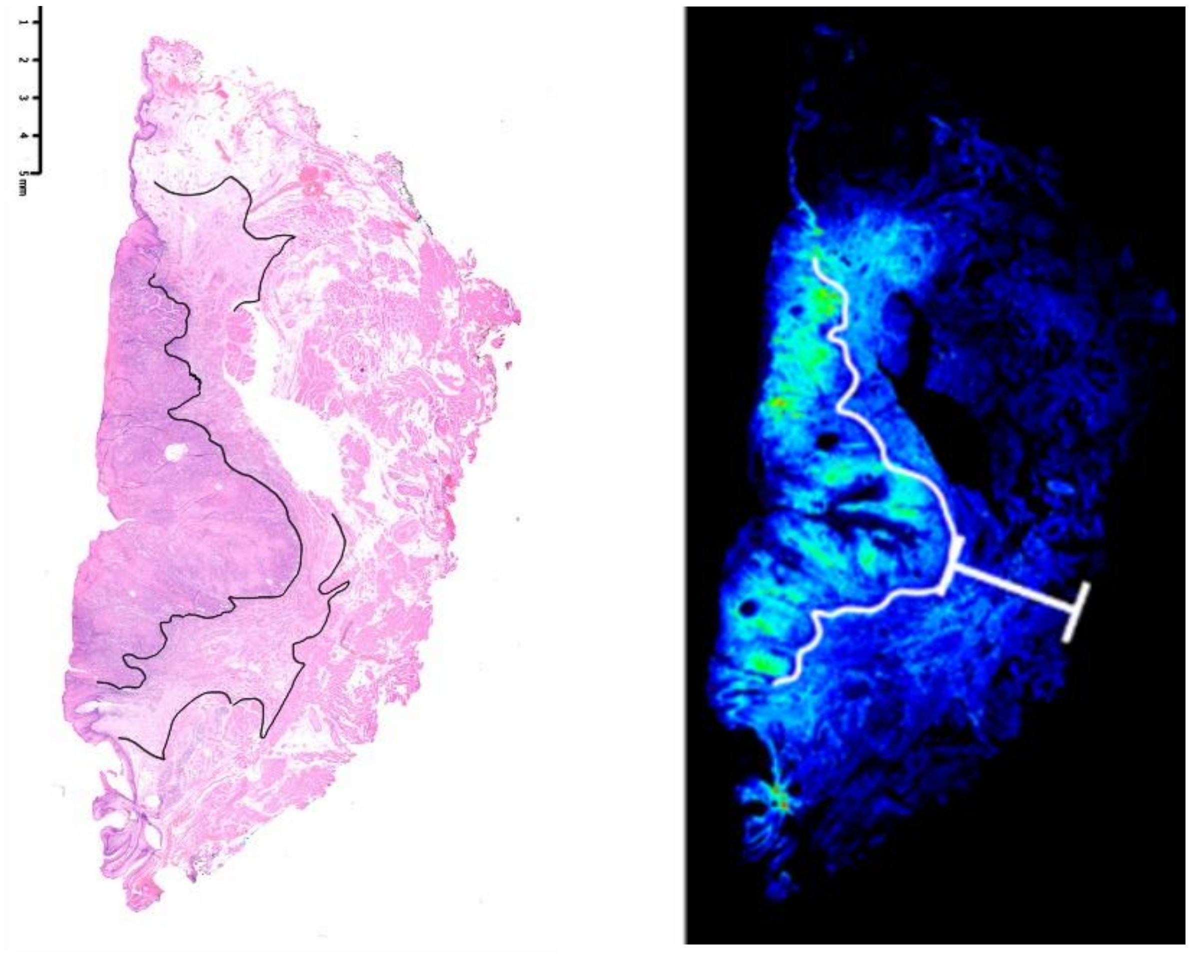

9. In the Figs. 2&4 and Supplementary Fig. 3, the bars are not explained and statistical analysis is not presented. Besides, these are several data points in the Supplementary Fig. 3 without any description.

We have revised the figures to clarify the presentation of the data and corresponding statistical analyses. Specifically, the statistical tests and significance indicators have now been added to Figure 4 and Supplementary Figure 4. In addition, all figure legends have been updated to provide complete descriptions of the displayed data and statistical methods, ensuring full clarity and transparency. The individual data points shown in Supplementary Figure 3 represent outliers, which are now explicitly described in the revised legend.

10. To ensure the reproducibility of this work, the imaging parameters for in vivo and ex vivo fluorescence imaging should be detailedly described, such as the excitation wavelengths, emission filters, exposure times, excitation power, and distance between the light source and tissues.

We thank the reviewer for emphasizing the importance of reproducibility and for suggesting the inclusion of more detailed imaging parameters.

For the in vivo imaging, we utilized the Quest® Spectrum 2.0 system, which uses excitation at 780 nm with a power density of 10 mW/cm^2, and emission is captured in the range of 800–900 nm. The camera offers gains of 9, 22.5 (default), or 36, and exposure times of up to 1000 ms (38 ms as the default). These settings can either be used in automated mode (set by the acquisition software, Capture Suite) or selected by the user based on the fluorescence signal of the sample. The working distance is between 20–30 cm.

For the ex vivo imaging, we used the Li-COR® PEARL Trilogy system, which employs excitation with 785 nm light at 3 mW/cm^2 power density, and emission is captured in the range of 810–830 nm. The gain and exposure time (ranging from 500 ms to 60 s) are automatically set by the acquisition software, Image Studio™, to produce a high dynamic range image. The user adjusts the working distance to achieve the best focus, depending on the specimen's height.

These details have been incorporated into the manuscript as follows:

Regarding the open camera system (Quest® Spectrum 2.0), we have added (page 5, line 175-179):

"This system utilizes excitation at 780 nm with a power density of 10 mW/cm^2*, and emission is detected in the range of 800–900 nm. In our study, the gain was set to 22.5 (default), and we tested an exposure time of 38 ms (default). To standardize imaging conditions, we aimed to keep the camera's distance between 20–30 cm and positioned it perpendicular to the sample, although slight variations occasionally occurred."*

Additionally, we have added the following for the closed-filed imaging system (page 5, line 185-189):

"This camera system uses excitation with 785 nm light at 3 mW/cm^2 *power density, and emission is captured in the range of 810–830 nm. The gain and exposure time (range of 500 ms to 60 s) are automatically set by the acquisition software (Image Studio™) to produce a high dynamic range image. We adjusted the working distance to achieve the best focus, which varied depending on the specimen's height."*

We hope these revisions provide the necessary details for reproducibility.

11. In the Methodology section, the description "Patients with synchronous biopsy-proven active malignancies or previous surgery, chemo- or radiotherapy to the oral cavity were excluded" is not consistent with "Thirty-one patients with biopsy-proven OSCC scheduled for standard-of-care surgery were included in this study" (in the Result section).

To clarify, when we stated, "Patients with synchronous biopsy-proven active malignances or previous surgery, chemo- or radiotherapy to the oral cavity were excluded", we used the word "synchronous" to refer to patients with second primary malignancies other than oral squamous cell carcinoma. However, to keep as clear as possible, we have adjusted the wording of this section (page 4, line 130-132):

"*Patients with biopsy-proven active malignancies other than OSCC were excluded, as were those with a history of surgery, chemo- or radiotherapy involving the oral cavity*".

12. The advantages of near-infrared fluorescence imaging over the label-free techniques (such as autofluorescence imaging) in detecting oral cancers are not introduced in the background. I believe some references can provide solid support regarding its capability to overcome the interference from blood and its high tissue-penetrating property, such as Nat. Photon., 2024, 18(12): 1254-1262; Nat. Mater. 2021, 20, 1571-1578; Nat. Nanotechnol. 2023, 18, 1195-1204; CCS Chemistry, 2022, 4(2): 476-486.

We have incorporated the advantages of NIR fluorescence imaging over label-free techniques into the manuscript as follows (page 3, line 79-83):

"Additionally, NIR FI offers significant advantages over label-free techniques (e.g., autofluorescence), including enhanced tissue penetration and minimized interference from blood, making it particularly effective for imaging deep tumor tissue. These advantages have been confirmed in recent studies, underscoring the potential of NIR FI to improve the accuracy of tumor detection during surgery[20, 21]."

This addition highlights the superior qualities of NIR FI, including better tissue penetration and reduced blood interference, as compared to label-free methods such as autofluorescence, which are critical for improving tumor detection during surgery. We believe this information strengthens the background on NIR FI and its relevance to the field of oral cancer surgery.

Reviewer 2

This is a well written, interesting paper on the added value of fluorescence imaging (FI) on surgical resection margins in SCC of the oral cavity. This technique appears to be promising and I think there is a realistic chance that it will supplement surgical management in the future. I also note prior well cited publications on the theme in Nature Communications, and so this article aligns with the journal's existing topical areas of interest. Noteworthy results include a novel fluorescence imaging agent, showing good tolerability in this patient sample (n=31) and potentially good tumour specificity. Also, a proportion (5/31) of patients in whom surgical approach and outcome was altered (favourably) due to FI findings, obviating the need for adjuvant radiotherapy. There are similar studies in the literature, but not at a significantly more mature stage of investigation (tending to be similar in terms of single centre, double-digit patient numbers), and so this study would contribute to the existing body of evidence. Clarification on some items would be beneficial with regards consideration for publication (and for inclusion in any future systematic review or meta analysis).

Items for clarification:

Abstract
1. Unless otherwise instructed by the journal, the abstract might be divided into background, method, results, conclusion.

We thank the reviewer for this suggestion, however, upon consultation with the senior editor, we were instructed not to change the format of the abstract. Therefore, the abstract remains as originally submitted.

Introduction:
2. Explicit definition of the study type is suggested (single centre, prospective, single arm... etc). In the introduction (line 89), the paper implies that it is a feasibility study. In the methods/ statistical analysis sub-section (line 230), it implies that it is a phase II study (elsewhere, the authors allude to their previous phase I/II study). Is it a feasibility study or other category of study/ trial? If a feasibility study, do the authors conclude that a larger multi-centre study/ RCT is feasible and desirable? If so, have the authors drawn inferences on the foreseeable resources, costs, regulatory, time-scale, organisational, and training implications, and/ or necessary patient numbers, risks and obstacles this might involve? Noting that 31 patients were recruited over an approximately 3 year interval.

To clarify, this study is a Phase I/II, prospective, single-center trial, and we have updated the main text to:

"This prospective single-center, single arm Phase I/II study was performed in 31 patients diagnosed with OSCC…." (page 4, line 119-121).

We agree on the importance of consistency in describing the study design and thank the reviewer for highlighting this point.

Looking ahead, we are in the process of setting up a multicenter randomized controlled trial (RCT) at the national level, supported by the Dutch Head and Neck Cancer Society and the Dutch Fluorescence-Guided Surgery Group.

Launching a multicenter RCT involves several key components: securing camera systems, ensuring tracer availability, and recruiting patients. Many of the intended participating centers already have the required camera systems, and for those that do not, we will provide the necessary equipment. As noted in our manuscript, tracer production is centralized at the LUMC, and they have expressed strong support for the development of this multi-center trial.

In terms of regulatory considerations, our primary goal is to generate data that will provide sufficient evidence to support a change in national clinical guidelines. Based on our calculations and the chosen endpoint (e.g., reduction in positive margins), we estimate that a patient cohort of 150-200 will be required, which is certainly feasible across multiple centers within 2-3 years. While the recruitment for our study took longer than expected due to logistical challenges, the experience gained has positioned us to address and overcome these challenges in the upcoming trial.

For training, we plan to adopt strategies similar to those used in sentinel lymph node (SLN) studies to mitigate any effects of the learning curve and ensure consistent and accurate application of the technique across centers.

To further clarify our next steps, we have added the following section to the Discussion (page 14-15, line 605-610):

"Building on the encouraging results from this Phase I/II study, the next steps involve the development of a multicenter randomized controlled trial to provide data that could inform potential changes in national clinical guidelines. By demonstrating the clinical benefits of FI in real-world settings, it is hypothesized that FI can be established as a standard tool in oral cancer surgery, ultimately improving surgical precision and patient outcomes. Preparations for this multicentre trial are currently ongoing."

3. Consider more explicit definition of primary and secondary objectives/ metrics. In the method section (line 134) and in the protocol (supplementary material), the authors suggest that an objective is to "determine whether FI could IMPROVE the rate of adequate tumour resections..." In the introduction section (line 107): "...we hypothesise that this approach ... reduce inadequate resection margins and ... enhance patient outcomes". Accepting that the study quantified a proportion of patients whose surgery was modified in order to avoid what would otherwise have been an inadequate resection, without a control arm, the objective might be reworded in terms of absolute efficacy metrics, rather than indicating comparative performance against standard of care (i.e. according to phase II level objectives). Consider stating quantifiable outcome measures.

Upon reflection, we agree that our study objectives and outcome measures should be more explicitly defined, and we have made the necessary revisions to clarify these points.

As the reviewer correctly points out, the primary endpoint of this Phase I/II study is safety, tolerability, optimal dose, and the optimal injection-to-imaging interval. The overarching goal of the study was to assess whether fluorescence imaging could reliably identify inadequate margins and influence intraoperative decision-making in oral cancer surgery. Importantly, we did not set out to directly assess the reduction in inadequate resections, but rather focused on evaluating the feasibility and effectiveness of fluorescence imaging in identifying tumor tissue and guiding surgical decisions to avoid inadequate margins.

We have revised the Introduction and Methods section to better reflect these objectives. Specifically, we have deleted the following sentences:

Page 4, line 112-114:

"By leveraging the insights gained from our Phase I/II study, we hypothesize that this innovative approach can lead to improved surgical precision, reducing inadequate resection margins and ultimately contributing to enhanced patient outcomes."

And,
Page 4, line 145-146:

"Ultimately, the study aimed to determine whether FI could improve the rate of adequate tumor resections, defined as margins ≥5 mm, in OSCC surgery."

Instead, we now focus on the core objectives of the study, which were to determine the safety, tolerability, optimal dose, and injection-to-imaging interval, as well as to evaluate how FI could assist in identifying inadequate margins and influencing intraoperative decisions.

4. Were potentially applicable study frameworks or reporting guidelines considered e.g. IDEAL in surgical innovation or STARD in diagnostic accuracy studies?

We did not initially consider the IDEAL or STARD frameworks, but after reviewing them, we can confirm that elements of both are relevant to our study. We would like to clarify that we have adhered to the CONSORT checklist for this study, as the journal specifically requested that we follow these guidelines. We believe that the CONSORT framework has provided a solid structure for reporting our trial, ensuring transparency and rigor in our methodology.

Regarding the IDEAL framework, we are indeed working in line with this model. Stage 1 has already been completed by colleagues at LUMC, who conducted studies on the tracer in healthy participants and colon cancer patients[4]. We are currently in Stage 2, which consists of 2a (safety) and 2b (efficacy) phases, as we are evaluating the safety and effectiveness of fluorescence imaging with cRGD-ZW800-1 in OSCC patients. The next steps, which will involve Stage 3 (assessment) and Stage 4 (long-term study), would include comparing the technique to standard of care (e.g., via randomized controlled trials) and assessing long-term outcomes. While we have not yet reached these stages, they represent important future directions for this research. As we discuss in the Discussion section (page 16, line 594-602), we aim to move towards these stages in the coming years.

In terms of the STARD guidelines, these are generally applicable to diagnostic accuracy studies. While we did not specifically follow the STARD checklist at the outset, we find there is substantial overlap with many of its recommendations. However, we acknowledge that our study does not fully meet all of the STARD criteria, particularly given the nature of our design and the absence of a formal control arm. We appreciate the reviewer for pointing this out, and we will certainly consider the STARD guidelines more explicitly in future studies of this nature.

5. line 86: "... has shown tumour-specific imaging without toxicity in healthy volunteers and colon cancer patients." Consider alternative wording in relation to healthy volunteers.

The term "healthy volunteers" was derived from the original publication where the study was first described[4]. However, to align with the reviewer's comment and enhance clarity, we have revised the text to use "healthy participants" instead. The updated sentence now reads (page 3, line 91-93):

"... has shown tumor-specific imaging without toxicity in healthy participants and colon cancer patients".

We appreciate the reviewer pointing this out, and we believe this wording is more appropriate for the context.

Methods:

6. Were the patients recruited consecutively (to address selection bias)? Have the authors considered reporting the study against criteria by which it might be considered for inclusion in a systematic review/ meta-analysis (e.g. CONSORT extension for feasibility studies or STARD)?

We thank the reviewer for this valuable comment. All eligible patients were recruited consecutively by screening cases discussed at a multidisciplinary meeting. This meeting includes all patients with oral squamous cell carcinoma (OSCC) treated by any of the head and neck surgeons at our center. This approach ensured that all eligible patients during the study period were considered for inclusion, thereby minimizing selection bias. We have revised a part of the Methods section to add this important note (page 4, lines 135-137):

"To minimize selection bias, all eligible patients were consecutively recruited after screening at a multidisciplinary meeting, ensuring all were considered for inclusion"

Regarding reporting standards, we confirm that the study was conducted and reported in accordance with the CONSORT 2025 guidelines. The manuscript has been reviewed against the corresponding checklist to ensure completeness and transparency of reporting. Furthermore, we have included the CONSORT checklist in our submission to support the study's adherence to these guidelines.

7. How many operators/ years experience? Was any training needed for the technique?

The surgeries in this study were performed by experienced head and neck surgeons, all of whom were highly skilled in standard surgical procedures (4-25 years of experience) but had no prior experience with fluorescence imaging. To ensure the proper application of the technique, each surgery was closely supervised by a team of medical fluorescence experts. These experts, with 2-3 years of direct experience in fluorescence imaging, have been involved in numerous studies, contributing to over 20 projects involving similar techniques. While the surgeons did not receive specific training in fluorescence imaging for this study, they were supported and guided by these experts throughout the procedures. The fluorescence experts had received formal training in fluorescence technologies, including specialized sessions on the camera systems used in this study. Moreover, the principal investigator, with more than 15 years of experience in fluorescence imaging, alongside technical staff who bring over 25 years of experience in spectroscopy, ensured that the technique was implemented accurately and effectively. The team's expertise was further strengthened by their active participation in conferences and professional networks, including the Dutch Fluorescence Guided Surgery Group (DFGS), which fosters collaboration and development in the field of fluorescence-guided surgery. These experiences provided a solid foundation for the successful integration of fluorescence imaging into this clinical study.

In response to this comment, we have clarified the methodology in the main text to reflect this additional detail. Specifically, we have added the following statement to the Methods section (page 5, lines 173–175):

"Open surgical-field intraoperative FI was performed using the CE-marked Quest Spectrum Platform (Quest Medical Imaging, Middenmeer, The Netherlands) by a team of experienced fluorescence imaging experts."

8. Is the fluorescent contrast agent an investigational medicinal product? If so, would this be governed/ overseen by a particular domestic regulatory body?

The fluorescent contrast agent used in this study is an investigational medicinal product (IMP). The study was conducted in accordance with the EU Clinical Trials Regulation (EU) and approved through the Clinical Trials Information System (CTIS). Authorization was granted by the Central Committee on Research Involving Human Subjects (CCMO, The Netherlands), which serves as the national competent

authority for clinical trials with IMPs. The study protocol and Investigational Medicinal Product Dossier (IMPD) were reviewed and approved by both the CCMO and the institutional ethics committee. All trial activities were conducted in compliance with Good Clinical Practice (GCP) and relevant national and European regulations.

In response to this comment, we have added the following sentence to the Methods section (page 4, line 123-126):

"*As this study involved the use of an Investigational Medicinal Product (IMP), authorization was granted by the Central Committee on Research Involving Human Subjects (CCMO, the Netherlands), which serves as the national competent authority of clinical trials with IMPs*."

Stats:
9. Did the authors consider including ROC/ AUC calculations?

We appreciate the reviewer's suggestion regarding the inclusion of ROC/AUC calculations. However, we did not incorporate this analysis into our study. Our primary objective was to assess whether fluorescence imaging could identify inadequate resection margins during surgery and influence intraoperative decision-making. As we also emphasized in our response to point 4 of Reviewer #1, we do not view fluorescence imaging as a stand-alone diagnostic test, but rather as an intraoperative tool to assist the surgeon in real-time decision making. While histopathology was used as the reference standard for margin assessment, our focus was on the clinical utility of fluorescence imaging in real-time decision-making rather than the quantitative assessment of fluorescence signal intensity.

ROC/AUC calculations are typically used when evaluating the diagnostic accuracy of a tool, especially when assessing the performance of a continuous variable (such as signal intensity) in distinguishing between two or more conditions. However, in our study, the decision-making process was not based on quantifying the fluorescence intensity signal but on whether fluorescence could detect inadequate resection margins, a decision-making step that directly influences the course of surgery. As such, ROC/AUC would not have provided additional insights into the effectiveness of fluorescence imaging in improving surgical outcomes in this specific context.

Given that fluorescence imaging was neither used nor intended as a diagnostic classifier, adding ROC/AUC analysis would not have advanced the study aims and would have introduced analytical complexity without offering clinically meaningful insight.

10. Line 226. "... this analysis tests the null hypothesis that the adequate surgical margin rate is at most 15% versus the alternating hypothesis that the adequate margin rate is at least 40%..." How were these percentages decided? Was it based on the quoted range of inadequate margin rates in the literature of 67-85%? If so, did the authors select the highest quoted inadequate margin rate to test the null hypothesis? Do the authors have an estimate of their own local centre's margin adequacy rates, as this might better contextualise these particular results (if available).

The percentage of 15% for the null hypothesis was based on historical data from our own center[5], reflecting the local margin adequacy rate. This percentage was not chosen arbitrarily, nor was it based on the highest quoted inadequate margin rates from the literature (67-85%). Instead, we used our center's own margin data to provide a more relevant and context-specific benchmark for comparison. This approach provided a meaningful baseline for assessing the impact of the fluorescence imaging technique. For the alternative hypothesis, we selected 40% as the target rate of adequate margins, which we considered to be a reasonable and achievable improvement based on an expert consensus within our

surgical team. We hypothesized that almost tripling the rate of adequate margins (from 15 to 40%) would be a significant clinical improvement.

When focusing on the positive margins, which have been shown in the literature to be the most prognostically relevant[6], we observed eight positive margins intraoperatively. Of these, two underwent an additional resection purely based on fluorescence imaging findings, resulting in only one positive margin at final histopathology. For this single case, the positive margin was detected using fluorescence imaging, but ultimately missed because the frozen section was taken just adjacent to the fluorescent spot. If the additional resection had been performed solely based on the fluorescence signal, the margin would have also been addressed. Thus, using fluorescence imaging, no positive margins would have remained postoperatively. In contrast, with standard intraoperative assessment alone, there would have been three positive margins at final histopathology. This illustrates the potential of fluorescence imaging to substantially reduce positive margins in our cohort.

To clarify this point, we have made the following addition in de Results section (page 11, line 450-454):

"This specific positive margin was detected using FI, but no additional resection could be performed as the frozen section was taken adjacent to the fluorescent area. Had we relied solely on FI, even this single positive margin could have been addressed, resulting in zero positive margins postoperatively. Without the use of FI, final histopathology would have revealed three inadequate margins, as two positive margins were missed during standard IOA."

12. With regards safety (Results, line 243). Were these classified according to Clavien Dindo or Common Terminology for AEs (CTCAE)?

Adverse events (AEs) and serious adverse events (SAEs) were classified according to the Common Terminology Criteria for Adverse Events (CTCAE). Importantly, no AEs were related to the administration of the fluorescent tracer itself, which is reassuring. During the study, three independent researchers systematically reviewed participants' medical records to identify complications. When a complication was identified, it was classified according to the CTCAE, the required treatment was documented, and its progression or resolution was monitored.

To clarify this, we added the following sentence to the Methods section (page 5, line 152-155):

"*Adverse events were monitored continuously by three independent researchers. When a complication was identified, it was classified according to the Common Terminology Criteria for Adverse Events (CTCAE), the required treatment was documented, and its progression or resolution was monitored*."

13. No AE were recorded in relation to the administered agent itself, which is encouraging. There were 21 AE/SAEs in a series of 31 patients. Is this higher than expected?

In total, 17 complications occurred in 12 of 31 patients (39%). This incidence is consistent with previous reports in the literature, where complication rates range between 9.3% and 64%, depending on the extent and complexity of the surgical procedure[7-9]. All complications were evaluated in collaboration with the treating physician and, when appropriate, other medical specialists involved in the patient care. The majority of complications in our cohort were typical of head and neck surgery, primarily bleeding and infection, and none were directly attributable to the fluorescence imaging procedure. Three complications were not typical of head and neck surgery, of which one head trauma, one urine tract infection, and one acute kidney injury. In the case of the renal insufficiency, medical specialists were consulted, and it was concluded that the renal insufficiency was due to dehydration and not related to administration of cRGD-ZW800-1. Furthermore, all adverse events were submitted to the local Medical Ethical Commission for review, and it was confirmed by that committee that these events were not related

to the administration of the fluorescent agent. A detailed summary of the complications and their management is provided in Supplementary Table 1. A detailed summary of the complications and their management is provided in Supplementary Table 1.

14. Did FI impact the post-surgical complications? For example, did the AE/SAEs predominate in cases where further resection was indicated on the basis of FI analysis?

Regarding the potential influence of a fluorescence imaging-based additional resection on postoperative complications, no evidence was found that FI contributed to additional morbidity. As described in our protocol, an additional resection was performed only in specific cases: (1) when a macroscopically inadequate margin was identified at a fluorescent spot, or (2) when frozen-section analysis of a fluorescent spot that appeared macroscopically adequate revealed an inadequate margin.
Thus, additional resections were performed only when histologically justified. These resections involved only a few millimeters of extra tissue and did not result in longer hospital stays or additional complications. Importantly, no additional resections necessitated an unplanned reconstruction.

15. Wider resections might impact other outcome measures e.g. length of stay, swallow etc. Was there a perceived trade-off, or was this felt to be beyond the scope of the study?

As the reviewer correctly noted, oral cancer surgery can affect functions such as speech and swallowing. Since the additional resections were minimal, no measurable impact on these oral-specific functions are expected. Moreover, achieving adequate resection margins remains the primary objective during oral cancer surgery, as margin status is directly linked to oncologic outcomes. Therefore, the decision to perform additional resections was guided solely by the goal of obtaining adequate margins, which is crucial for reducing recurrence risk and improving survival. While functional preservation and quality of life are important considerations, they are secondary to achieving oncologic safety.

Looking forward, we believe fluorescence-guided surgery has the potential to enhance both surgical precision and patient outcomes. Currently, surgeons typically resect an additional 10 mm of surrounding tissue to ensure oncologic safety. With fluorescence imaging, this extra margin may no longer be necessary, allowing for smaller and more targeted resections. As a result, this approach could reduce the impact on functional outcomes and improve quality of life, without compromising oncologic safety.

We have clarified these aspects in the Results section to improve transparency (page 7, lines 285-288):

"Furthermore, the use of FI did not contribute to further morbidity. Additional resections were performed only when histopathologically justified and involved only a few millimeters of extra tissue, which did not necessitate unplanned reconstructions and therefore did not prolong hospital stay."

This highlights that all extra resections were limited, necessary, and did not lead to increased complications or length of stay, ensuring the safety of the technique.

16. Table 1. Study characteristics (p.20). In the column "inadequate margin found with FI" (Yes/No). In every case with a stated result in this column (24/32), the result is a 'YES'. In the other 8/32 cases, the result is recorded as a '-'. What does this mean? Is '-' equivalent to a 'NO', or does it mean that margin assessment with FI was not performed in these cases? If it is a 'NO', it may add clarity to state this (or otherwise explain), so as not to imply that FI was 'positive' in every case where it was employed. Presumably there were cases where FI was 'negative', as non-zero true and

false negative rates are quoted in Table 2 (supplementary material). The authors state that all positive margins were detected with FI, but document 1 false negative in Table 2. Please clarify.

We have clarified the information in Table 1 to address the concerns raised. The columns labeled "Inadequate margin found with FI" and "Inadequate margins found with IOA" originally used a "-" to indicate cases where the intraoperative margin status was classified as "clear," meaning no inadequate margins could be detected using either FI or standard IOA. To avoid confusion, we have now revised the "-" to "n/a" (not applicable), as it better reflects that margin assessment with FI was performed, but no inadequate margin was identified (page 22, line 842).

Furthermore, we wish to emphasize that Table 1 exclusively focuses on resection margins and their adequacy; therefore, malignant tissue not affecting margin status was intentionally excluded. In contrast, Supplementary Table 2 reports data on the detection of all malignant lesions. Within this dataset, we identified one false-negative case involving microsatellite lesions that was missed by FI. However, because this finding did not result in an inadequate margin, it did not influence the results presented in Table 1.

To enhance clarity, we have also updated the table titles to better reflect their respective scopes. Table 1 is now titled "*Study characteristics and inadequate resection margin detection using FI with cRGD-ZW800-1*", emphasizing its focus on margin status. Supplementary Table 2 is now titled "*Diagnostic performance of FI with cRGD-ZW800-1 in detecting malignant lesions*", highlighting its focus on overall lesion detection. In addition, we have expanded the legend of Supplementary Figure 3 to include detailed explanations of the origin and number of true/false positive and negative cases.

We hope these clarifications resolve any ambiguity and facilitate a clearer interpretation of the data.

Discussion:

17. line 444: "...FI altered decision making in specific cases by identifying inadequate margins..." Consider stating/ repeating the number/ proportion of applicable cases.

We have revised the sentence to specify the number of applicable cases. It now reads (page 12, lines 482-484):

"Most importantly, FI altered surgical decision-making in five cases (16%, 5/31) by identifying inadequate margins that were overlooked during routine IOA, underscoring the clinical value of intraoperative FI".

18. Line 418: "... intraoperative FI was validated through objective in vivo quantification..." Perhaps this conclusion would be pending external centre reproducibility of findings/ multi-centre study?

We agree that the term "validated" may suggest external or multicenter confirmation, which was not the intention in this context. Our aim was to emphasize that, unlike previous studies relying primarily on qualitative assessment, our study employed objective in vivo quantification to substantiate the fluorescence signals measured during surgery. To better reflect this, we have replaced "validated" with "substantiated" and revised the sentence to read (page 12, line 491-492):

"For the first time, intraoperative FI was substantiated through objective in vivo assessment of tracer pharmacokinetic using MDSFR/SFF spectroscopy."

19. Line 431: Typo "... for wat least..."

The phrase has been corrected from "for wat least" to "*for at least*" (page 12, line 504).

20. Line 466: Limitations statement is quite brief.

In response, and in line with the comments of other reviewers, we have expanded the limitations section to provide a more comprehensive discussion. These revisions can be found on pages 12-15.

Thanks to the authors for this engaging read, and to the journal for the invitation to review.

Reviewer 3

The goals of this study were to study the safety and feasibility as well as the performance of fluorescence imaging (FI) to improve surgical resection margins in oral cancer. The paper is hard to follow due to the presentation of the results.

1. One of the objectives of the study was to identify the optimal dose of the agent, however in the end the analysis included patients studied at all 3 doses (7 studied at 0.05, 3 studied at 0.01 and 21 studied at 0.025). Since the dose 0.025 was the dose deemed to have optimal performance and the power calculations and protocol defined the rate of adequate surgical resection margins based on the patients evaluated at the optimal dose, for consistency and generalizability to future studies, the primary endpoint should be assessed only among patients evaluated at the the optimal dose. Furthermore, the decision rule was based on 21 patients and this calculation should be highlighted in the paper. Table 1 is based on 32 patients further confusing the results.

We thank the reviewer for this insightful comment and fully understand the concern regarding consistency and interpretability of the primary endpoint. Since a fluorescent signal was detected in all patients, we included all patients in the analysis. This approach was explicitly defined in our Methods section, as noted on page 7, lines 250-251: "*All patients with a detectable fluorescence signal were included in the surgical margin evaluation, which ultimately comprised the entire cohort (n=31). Since signal detectability could not be guaranteed in advance, the required sample size was calculated solely for the optimal dose cohort*". Had not all patients shown a detectable fluorescence signal, we would have limited the primary outcome calculation to the optimal dose cohort only, as this was the cohort for which the sample size calculation was originally performed.

However, after extensive internal discussion, we agree that presenting the primary endpoint based solely on the 21 patients evaluated at the optimal 0.025 mg/kg dose provides a clearer and more consistent reference for future studies.

For transparency, we have now included both the full cohort (31 patients) and the 21 patients at the optimal dose side by side in the text. In the optimal-dose group (21 patients), 14 patients had inadequate margins, comprising 6 positive and 8 close margins. All inadequate margins were identified using fluorescence imaging, while standard intraoperative assessment missed 7 cases, including 3 positive margins. In the full cohort of 31 patients, 23 patients had inadequate margins (8 positive, 15 close), of which 9 were missed using standard intraoperative assessment. Importantly, fluorescence imaging detected all inadequate margins in both analyses.

Regarding the previously confusing count of 32 patients, we have corrected Table 1. The original number was based on patient IDs rather than the actual number of patients. To avoid further confusion, we have updated Table 1 to reflect the true number of patients, which is 31.

We have clarified this distinction in the Results section of the manuscript (page 9, lines 363-370):

"In the full cohort (n=31), 15 close and 8 positive margins were all successfully detected using FI, whereas nine inadequate margins were missed by standard IOA by surgeon and pathologist (three positive and six close margins). Focusing on the optimal-dose cohort (n = 21), all 14 inadequate margins (6 positive and 8 close margins) were again detected by FI, while IOA missed seven cases (three positive, four close). Consequently, FI demonstrated a significant higher inadequate margin detection rate compared to standard IOA for both the full cohort (100% vs. 61%, Exact binomial McNemar's test, p = 0.004) and the optimal-dose cohort (100% vs. 50%, Exact binomial McNemar's test, p = 0.008)."

2. It is not clear why IOA was considered the gold standard (pg. 13, 475), shouldn't the postoperative margin status be the gold standard for the calculations of sensitivity and specificity in Suppl Table 2?

Within our study, the margin status could be assessed histopathologically during surgery (intraoperative) using standard intraoperative assessment. This method is currently the gold standard in the Erasmus MC for intraoperative margin assessment. This approach allowed us to directly correlate the findings from intraoperative fluorescence imaging with the intraoperative histopathological status, allowing fluorescence-guided extra resections. Importantly, the ultimate evaluation of the efficacy (including diagnostic performance) of fluorescence imaging was based on the definitive histopathological assessment after surgery (postoperative margin status). To clarify this, we have adjusted two sentences in the Methods section, including:

Page 5, line 190-191: *"IOA is standard-of-care for intraoperative margin assessment at Erasmus MC"*

Page 7, line 253-254: *"The primary endpoint is the rate of adequate surgical resection margins, based on the postoperative margin status"*

3. Are calculations in that table at the lesion level or patient level? Why can you have Intraoperative margin status Positive, but No inadequate margin found with IOA (pt ids 27 and 30)? Why patients that were Clear on Intraoperative margin status do not have a status for Inadequate margin found with FI? How do you establish specificity in that case? More details on how Suppl Table 2 was obtained should be provided.

Supplementary Table 2 presents lesion-level data, as some patients could have multiple malignant lesions and an inadequate margin. We have changed the name of Supplementary Table 2 to "Diagnostic performance of FI with cRGD-ZW800-1 in detecting malignant lesions" to clarify this. We have also expanded the legend to explain how true positives, false positives, true negatives, and false negatives were defined, hopefully clarifying the lesion-level analysis. This provides transparency on how sensitivity and specificity were calculated.

Regarding Table 1, cases in which the intraoperative margin status was positive, but no inadequate margin was found with IOA (patients 17, 26, and 29) reflect situations where a positive margin was missed during IOA, but subsequently identified on final histopathology. In contrast to IOA, FI was able to detect all these inadequate margins intraoperatively. Furthermore, the columns labeled "Inadequate margin found with FI" and "Inadequate margins found with IOA" originally used a "-" to indicate cases where the intraoperative margin status was classified as "clear," meaning no inadequate margins could be detected using either FI or standard IOA. To avoid confusion, we have now revised the "-" to "n/a" (not applicable), as it better reflects that margin assessment with FI was performed, but no inadequate margin was identified (page 22, line 842).

4. The paper (pg. 10, 344-347) states that "Importantly, in four patients, the tissue samples for assessment were mistakenly taken from areas immediately adjacent to, rather than directly from, the fluorescent spots identified by FI. This sampling error led to false-negative IOA results and missed opportunities for additional resections." however this contradicts the statement (pg. 6, 169-170) that "To prevent bias, both surgeon and pathologist were blinded to ex vivo FI findings. Fluorescent signals at specimen margins were only disclosed after standard IOA was completed." Please clarify.

The pathologist and surgeon were indeed blinded to the fluorescence signal before performing standard IOA. After finishing IOA, the fluorescent signals, if present, were disclosed to the pathologist and surgeon. We stated this on page 5, line 195-196: *"Fluorescent signals at specimen margins were only disclosed after standard IOA was completed"*).

As outlined in Figure 6 of our protocol (which is added to the submission), when a fluorescent spot was identified after FI, the pathologist first performed a macroscopic assessment of the specific spot. If the pathologist considered the spot macroscopically suspicious for tumor, an additional resection could be performed immediately. If the spot appeared macroscopically non-suspicious, fresh frozen sectioning was performed to confirm tumor histopathologically. In case tumor was confirmed, an additional resection could be performed. In the four patients mentioned on page 10 (line 405-406), the sampling error occurred during this step, as the fresh frozen section was taken adjacent to, rather than directly from, the fluorescent spot. This resulted in missing these inadequate margins during IOA. This highlights the practical challenge of accurately sampling the exact location of fluorescence for fresh frozen section analysis.

5. It is not clear how the 4 patients whose "inadequate margins could not be addressed intraoperatively due to difficulties in localizing the fluorescent signal for targeted frozen section sampling" were counted towards the primary endpoint and towards the accuracy metrics. Please clarify in Table 1 and Suppl Table 2.

These specific four patients were not considered false negatives. The issue was not a limitation of fluorescence imaging itself, but rather a practical challenge in accurately sampling the fluorescent spot for fresh frozen section. Definitive postoperative histopathology confirmed that the fluorescent signal corresponded to the inadequate margin in all cases. This actually underscores the added value of FI and suggests that greater reliance on the fluorescence signal could improve intraoperative margin management. We plan to incorporate this insight into the design of future studies.

In response to this, and in combination with the feedback provided by another reviewer (point 7, Reviewer #4), we have made the following revisions to the Discussion section (page 14, line 583-591):

"Nonetheless, in four cases, accurately targeting fluorescent signals for intraoperative frozen section sampling proved challenging, limiting immediate resection. Importantly, this did not reflect failure of FI itself, as postoperative histopathology confirmed that the fluorescent signal accurately corresponded to the inadequate margins in all cases. These observations underscore the potential of FI to reliably identify inadequate margins, while also illustrating the technical learning curve in integrating FI into clinical practice and highlight the need for standardized protocols and training to ensure accurate localization and sampling. Implementation of FI in standard surgical practice could improve intraoperative decision-making, provided that its use is optimized through appropriate training."

6. The paper (pg. 10, 336-337) states that in 9 out of 31 patients, FI revealed inadequate margins that were not identified by standard IOA, however the paper should also state that only some of these patients had additional resection performed.

We fully agree that it is crucial to clarify that not all inadequate margins detected by fluorescence imaging led to additional resections, as this distinction is highly relevant for interpreting the clinical impact of FI. In response, we have expanded the Results section (page 10, line 416-424) to clearly describe which margins prompted additional resections, and the reasons why others did not. This clarification ensures transparency regarding the efficacy of FI in guiding intraoperative decision-making and highlights both the potential of FI to identify missed inadequate margins and the practical limitations that can prevent further resection, such as tissue sampling errors or proximity to critical structures:

"Fluorescent signals detected on the deep margins of the specimens, areas not deemed clinically suspicious during IOA, prompted further examination with frozen sections. This identified nine inadequate margins that had been missed by IOA (three positive, six close). In two positive and three close margins, this resulted in additional resections. Final histopathological analysis confirmed that these extra resections were free of malignant tissue, thereby ensuring that all five patients ultimately achieved wider surgical margins. Notably, in three of these cases, the FI-guided additional resections allowed for the avoidance of adjuvant radiotherapy. In the remaining four cases, no additional resections were performed due to wrong tissue sampling (n=3) and tumor proximity to critical structures (n=1), as previously explained."

7. Given that the decision to perform additional resection was still the surgeon's decision, we can't entirely claim that the improvements in the rate of adequate resection margins are due to FI-guided.

We would like to emphasize that, according to our institutional surgical protocol, additional resections are not performed when standard intraoperative assessment does not indicate the presence of an inadequate margin. In five cases where additional resections were undertaken, the decision was prompted exclusively by fluorescence imaging findings. Specifically, frozen section analysis of tissue obtained from fluorescent regions, considered unremarkable during intraoperative assessment, revealed histopathological evidence of tumor involvement. Consequently, the subsequent resections were directly guided only by fluorescence imaging and would otherwise not have been performed after standard intraoperative assessment. Therefore, the improvement in the rate of adequate surgical resection margins observed in this study can be confidently attributed to the added diagnostic value of intraoperative fluorescence imaging.

Reviewer 4

This is a very interesting trial addressing feasibility, safety, and utility of fluorescence optical imaging for surgery of oral cavity carcinoma. The authors report the results of a prospective study on 31 patients, with findings ranging from optimal dose identification to impact on intraoperative decision making and histopathological assessments. Overall, the study was well designed, conducted, and reported.
I list here some points to be discussed in the context of this peer review, and that could potentially elicit revision of the manuscript:

MAJOR POINTS:
1. "ZW800-1, which binds specifically to integrins (αvβ1, αVβ3, αvβ5, αvβ6, αvβ8, α5β1, α8β1 and αIIbβ3)" -> does it mean that the the drug used in the study binds several other integrins apart from αvβ6 (which was studied through IHC)? How this fact impact on the biological and clinical value of this method? Is it a potential source of inaccuracy of the method used herein?

We thank the reviewer for this insightful comment. Indeed, the RGD sequence binds with high affinity to several integrins, including all five αv integrins (αvβ1, αvβ3, αvβ5, αvβ6, and αvβ8), two β1 integrins

(α5β1 and α8β1), and αIIbβ3[10, 11]. These integrins mediate cell adhesion and signaling processes, and their expression can vary between normal and malignant tissues. In this study, αvβ6 was selected for immunohistochemical validation because it is one of the RGD-binding integrins most frequently reported to be upregulated in epithelial cancers, while the expression level is low in most healthy adult epithelia[12]. Moreover, a well-validated and specific antibody for αvβ6 was available in our laboratory, whereas reliable immunohistochemical assays for other RGD-binding integrins are not routinely established. Therefore, αvβ6 served as a representative marker for assessing tracer specificity. Importantly, previous preclinical and first-in-human studies using the same tracer demonstrated that, despite its affinity for multiple integrins, tracer accumulation predominantly occurs in tumor tissue where these integrins are overexpressed, with minimal nonspecific uptake in normal tissues[4, 13].

While cRGD-ZW800-1 may theoretically bind to multiple integrins, we did not observe any clinically relevant fluorescence in tumor-negative tissue. Physiological signal was occasionally noted in salivary glands, consistent with known integrin expression in ductal cells, but this was easily distinguishable from tumor tissue by both surgeon and pathologist. Importantly, the intraoperative fluorescence signal showed excellent spatial correlation with the tumor location and with αvβ6 expression on IHC, enabling specific tumor delineation and identification of all inadequate surgical margins, including those that were not detected by the surgeon or pathologist during intraoperative assessment. CRGD-ZW800-1 is a well-established targeting motif for cancer detection, also applied in cRGD-based PET studies[14], and its ability to bind multiple integrin subtypes did not affect the biological or clinical accuracy of the method. Potential off-target binding would have manifested as nonspecific fluorescence, which was not observed, further supporting the robustness of the tracer for intraoperative margin assessment.

2. "In the remaining three cases, fluorescence was detected in non-suspicious anatomical structures: the mandibular bone marrow at the resection plane (n=2) and lingual salivary gland tissue (n=1). None of these areas were deemed clinically suspicious, and the corresponding margins on the tumor specimen were histologically tumor-negative." -> the fact that minor salivary glands and bone marrow are fluorescent is a potential source of bias, particularly if one thinks to a broader application of this technique. To limit such limitations, authors allude to the fact that clinical appearance of these tissues would be different to connective tissues were tumor foci could be located. Still, I find it very arbitrary and subjective, and thus prone to bias. This aspect mandates more elaboration in the Discussion in my opinion.

In our study, a small number of false-positive fluorescence signals were observed in non-suspicious anatomical structures, specifically the mandibular bone marrow and salivary gland tissue. These structures were not considered clinically suspicious during surgery, and subsequent histopathological examination confirmed that the corresponding resection margins were tumor-negative.

While such false-positive signals could theoretically necessitate additional fresh frozen sections, in practice this imposes only a limited increase in intraoperative workload. Fluorescence imaging facilitates more selective and targeted tissue sampling relative to standard intraoperative assessment, thereby mitigating unnecessary interventions. Importantly, all areas flagged as potentially suspicious undergo systematic histopathological evaluation prior to any additional surgical action. Consequently, although occasional supplementary sampling may modestly prolong intraoperative assessment, this does not adversely affect clinical outcomes nor compromise the overall validity of the technique.

We have added the following clarification to the Discussion section (page 13, line 538-546) to further elaborate on the clinical relevance of the observed false-positive fluorescence signals:

"Lastly, a false-positive fluorescence signal was observed in the mandibular bone marrow. Importantly, all of the aforementioned false-positive signals, including those detected in uninvolved gingiva and salivary gland tissue, were clinically not suspicious for malignancy. Each fluorescent area underwent careful histopathological evaluation, thereby confirming the absence of malignant tissue. As a result, these signals did not influence intraoperative decision-making, lead to unnecessary resections, or compromise patient safety. This observation underscores that, although such false-positive signals may occur, their presence can be effectively managed through standard pathological confirmation without impacting the clinical utility of fluorescence-guided surgery."

3. Definition of inadequate margin: the study is based on the assumption that margin below 5 mm is inadequate. Although authors often report separate data on positive vs close margins, they are taken altogether in terms of statistics. This assumption does not rely on sound evidence and should be critically discussed. There is evidence suggesting that the average threshold to define close margin is actually lower (10.1001/jamaoto.2017.0548, 10.1001/jamaoto.2016.4238). Also, studies suggest that such threshold could change according to the pattern of invasion of the tumor, thus taking into account biological heterogeneity in the context of squamous cell carcinoma (10.1002/hed.26956).

The reviewer highlights an important consideration regarding the definition of an inadequate surgical margin in head and neck pathology. Indeed, there is ongoing discussion in the literature, and evidence suggests that the threshold for a "close" margin may vary depending on tumor subsite, pattern of invasion, and other biological factors[15, 16]. In our study, we adopted the definition recommended by the Royal College of Pathologists[17] and National Comprehensive Cancer Network (NCCN) guidelines[18], which defines margins below 5 mm as inadequate. This threshold is also in accordance with our current clinical treatment protocol.

Furthermore, for the purpose of evaluating the efficacy of fluorescence imaging in identifying close or positive margins, the precise cutoff is less critical. We defined a clear threshold of 5 mm and assessed whether fluorescence imaging could reliably detect margins below this threshold. While it is recognized that this threshold may vary for certain subsites or specific clinical circumstances, this does not affect the validity of our study's findings regarding the performance of the imaging technique.

For the purpose of evaluating the efficacy of fluorescence imaging in identifying close or positive margins, the precise cutoff is not the focus of this study. A predefined threshold of 5 mm was applied to assess whether fluorescence imaging could reliably detect margins below this limit. Considering the optical properties of tissue, deeper signals are inherently more challenging to detect; therefore, margins smaller than 5 mm would be anticipated to be detected with greater ease using this technique. The reviewer's observation that the threshold defining inadequate margins may be lower than 5 mm further underscores that fluorescence imaging would be expected to detect such margins even more readily, given that smaller margins lie closer to the tumor surface. Moreover, fluorescence imaging provides a controlled and reproducible approach for intraoperative margin assessment, making it particularly well-suited for future investigations aimed at refining the definition of an adequate surgical margin. Given the relevance of this topic, we have added a dedicated paragraph in the Discussion section to provide further elaboration (pages 13-14, line 562-573):

"Within head and neck pathology, the definition of an inadequate surgical margin remains a topic of debate. Literature suggests that thresholds for close or positive margins may vary depending on tumor subsite, biological characteristics, and factors such as the worst pattern of invasion (WPOI)[50,51]. In this study, we applied a 5 mm cutoff in line with our institute's protocol and consistent with the Royal College of Pathologists and National Comprehensive Cancer Network (NCCN) guidelines[52,53]. Importantly, the exact cutoff is less critical for the purpose of evaluating FI, as the primary goal was to assess whether this

technique could reliably identify margins that are narrower than the defined threshold. While this approach allows for standardized assessment within our cohort, it is important to acknowledge that other institutions or clinical settings may adopt different margin definitions based on local practice. Nevertheless, because of *these variations, our results demonstrate that fluorescence imaging has the potential to enhance intraoperative margin detection, independent of the specific numerical cutoff applied."*

4. Moreover, comparing fluorescence imaging with intraoperative frozen section-based assessment of margins is partially biased by the fact that frozen section is not capable of detecting close margins, but only positive margins. Clinical evidence of the comparison is evident and clear, but I would find it fairer if authors clearly state that the methods that was used for comparison can't identify most cases that they defined with inadequate margins.

We appreciate that the reviewer found our clinical comparison clear and well-presented.

However, we respectfully disagree with the reviewer's statement that the method used for comparison cannot identify most cases of inadequate margins. The intraoperative frozen section-based assessment used in our study is capable of detecting both close and positive margins. To clarify this, within oral cancer surgery, two distinct approaches to intraoperative margin assessment exist.

The first is the wound bed-driven (defect-driven) assessment, where frozen sections are taken from the wound bed. Previous studies have shown that this approach does not improve oncological outcomes, mainly because additional resections are often not feasible due to the difficulty of accurately identifying the location of the positive frozen section[19-23]. Moreover, this technique can detect only positive margins and no close margins. In contrast, the specimen-driven assessment allows the detection of both positive and close margins and has been demonstrated to be superior to the wound bed–driven approach[22, 24-27]. For this reason, the AJCC Cancer Staging Manual (8th edition) recommends specimen-driven analysis. In our institution, this method is standard practice. We also employ a "parallel tagging" technique, in which matching numbered tags are placed both on the specimen and in the wound bed to ensure accurate relocalization in case an additional resection is needed[28, 29].

Within the Methods section, we described that our intraoperative assessment was specimen-based on page 5, lines 189-190:

"*After closed-field FI, specimen-driven intraoperative assessment (IOA) was performed*."

5. "high sensitivity (95.8%) and specificity (80%)" -> definition and number of false positive, false negative, true positive and true negative should be clearly reported.

This issue has also been highlighted by another reviewer, and we appreciate the opportunity to clarify our approach.

As requested, we have now clearly reported the diagnostic performance of our method, including the number of false positive, false negative, true positive, and true negative results, in Supplementary Table 2. In addition, we have provided detailed definitions of these terms within the table to ensure transparency and clarity in the interpretation of these metrics.

A critical aspect that we wish to highlight is the physiological fluorescence that was observed in salivary glands, which is consistent with known integrin expression in ductal cells. While this results in what can technically be considered a false positive signal, it is important to emphasize that the salivary glands were easily distinguishable from tumor tissue by both the surgeon and the pathologist during surgery. Furthermore, we would like to emphasize that we do not view fluorescence imaging as a standalone diagnostic test but rather as an intraoperative tool to assist the surgeon in real-time decision-

making. Therefore, we believe the inclusion of salivary glands in the diagnostic performance calculations should be guided by clinical relevance. In a typical surgical setting, salivary glands are not considered suspicious for tumor tissue and, regardless of their fluorescence intensity, would not undergo further investigation (such as fresh frozen sectioning). Thus, the presence of fluorescence in these tissues does not alter clinical management, as they would not be flagged as suspicious and would not affect surgical decisions. For these reasons, it is more appropriate to calculate specificity excluding the salivary glands to assess the true clinical value of fluorescence imaging in the operating room.

We have revised the manuscript to further clarify the methodology concerning the specificity calculation and the intended role of fluorescence imaging in our study. Specifically, we have added the following text to the Methods section (page 7, line 261-267):

"Diagnostic performance was determined based on sensitivity, specificity, negative predictive value (NPV), positive-predictive value (PPV) and accuracy in detecting inadequate resection margins. As FI is not intended as a standalone diagnostic tool, but rather is an adjunct to guide real-time surgical decision making, only clinically relevant tissues were included in this analysis. This approach ensures that only these clinically relevant tissues, which could potentially influence surgical management, were included, providing a more accurate reflection of FI's performance in real-world clinical setting."

We trust that these revisions address the reviewer's concern and provide additional clarity regarding the role of salivary glands in the diagnostic performance of our method. We appreciate the reviewer's thorough consideration and believe that the changes enhance the overall quality and transparency of the manuscript.

6. In the paragraph "Effect of FI on intraoperative decision making" it is difficult to get an overall measure of impact on decision making. The authors should report the number (and proportion) of cases with decision-making actually impacted by the use of fluorescence imaging, before deepening into details of specific cases.

We thank the reviewer for raising this important point, which has also been mentioned by another reviewer. We agree that it would be beneficial to provide a clearer overview of the overall impact of fluorescence imaging on intraoperative decision-making before delving into the details of specific cases.

In response to this, we have revised the Results section to include a more explicit summary of the impact of fluorescence imaging on decision-making. Specifically, we now report the number and proportion of cases where the use of FI impacted the decision-making process. These changes can be found on page 10, lines 389-414, and the updated text reads as follows:

"As all dose cohorts showed a detectable fluorescence signal using both in vivo and ex vivo FI, all patients (n=31) were included in margin assessment. As previously reported, FI combined with standard IOA revealed inadequate margins in 23 patients (74%, 23/31), including eight patients with positive margins and 15 with close margins (Table 1). Additional resections were performed in 70% of the patients with inadequate margins (16/23), comprising seven positive margins and nine close margins. Notably, 31% (5/16) of these resections were based exclusively on FI findings, which had been overlooked during the initial IOA. In contrast, no further resections were undertaken in 30% (7/23) of patients with inadequate margins, which included one with a positive margin and six with close margins. In one patient with a close margin, an additional resection was not feasible due to tumor proximity to critical anatomical structures. Furthermore, in one patient no IOA was performed; in this case, a pre-emptive additional resection had already been carried out during surgery and was deemed sufficient at the time. However, definitive pathology later revealed a close margin elsewhere, which, in retrospect, was apparent on FI. Importantly, in four patients, the tissue samples for assessment were mistakenly taken from areas immediately adjacent to, rather than directly from, the fluorescent spots identified by FI. This sampling error led to false-negative

IOA results (one positive and three close margins) and missed opportunities for additional resections. These cases highlight an important limitation of relying solely on frozen section analysis. Had FI alone guided resection decisions, these inadequate margins would have been identified and addressed intraoperatively. Lastly, in one patient, additional resection was not performed because achieving clear margins would have required a marginal mandibular resection, and the periosteum was clinically intact. In accordance with our standard practice, the surgeon deliberately opted against further resection."

We hope that this revision provides a clearer and more concise overview of the overall impact of FI on intraoperative decision-making. By explicitly reporting the number and proportion of cases in which FI influenced decision-making, we believe that the revised text better addresses the reviewer's concern while maintaining the detailed description of individual cases where applicable.

We trust that these revisions improve the clarity of our manuscript and adequately address the reviewer's request.

7. "Importantly, in four patients, the tissue samples for assessment were mistakenly taken from areas immediately adjacent to, rather than directly from, the fluorescent spots identified by FI. This sampling error led to false-negative IOA results and missed opportunities for additional resections. These cases highlight an important limitation of relying solely on frozen section analysis." -> these sentences appear to carry an important message, namely that fluorescence imaging can drive sampling for frozen section. I believe this message deserves more emphasis.

We agree that the potential of fluorescence imaging to guide frozen section sampling should be emphasized more. In response, in combination with the feedback provided by another reviewer (point 5 from Reviewer #3), we have expanded the Discussion to highlight this important point (page 14, line 583-591):

"Nonetheless, in four cases, accurately targeting fluorescent signals for intraoperative frozen section sampling proved challenging, limiting immediate resection. Importantly, this did not reflect failure of FI itself, as postoperative histopathology confirmed that the fluorescent signal accurately corresponded to the inadequate margins in all cases. These observations underscore the potential of FI to reliably identify inadequate margins, while also illustrating the technical learning curve in integrating FI into clinical practice and highlight the need for standardized protocols and training to ensure accurate localization and sampling. Implementation of FI in standard surgical practice could improve intraoperative decision-making, provided that its use is optimized through appropriate training."

8. "In general, the deep margin is rightly considered more important than the mucosal margin" -> this is untrue. The mucosal margin is as prognostically important as the deep margin. The latter might be more difficult to be adequately delineated, but this does not relate to importance.

The mucosal margin is indeed as prognostically important as the deep margin, and we apologize for the misstatement in the manuscript. What we intended to convey is that in our study, we observed no inadequate mucosal margins, and that clinically, the difficulty for the surgeon primarily lies in correct assessment of the deep margin.

We want to emphasize that our method provides excellent delineation of mucosal (Figure 3C and 3G) and deep tumor margins (Figure 3H), and both were carefully evaluated in this study. While the clear visualization of mucosal margins is highly reliable, we deliberately focus on the deep margin, as this represents the primary clinical challenge. Unlike some previous studies that focused solely on mucosal margins[30], our approach ensures detection of the more technically demanding deep margin,

reflecting our commitment not to be satisfied with mucosal delineation alone. We have revised the manuscript by deleting the following sentence (page 13, line 532-533):

"..., and no significant difference in recurrence rate was found between close and clear mucosal margins."

And adding the following sentence (page 8, line 325-327):

"This indicates that mucosal delineation can be achieved with well-defined boundaries and high contrast using FI, resulting in adequate identification of all mucosal margins."

9. "As such, this study likely underestimates the true added value of FI" -> this sentence is at risk of being simplistic; whether the study under- or over-estimates the added value of fluorescence imaging very much depends on how outcomes are measured. As stated above, if one considers inadequate margin everything below 5 mm and compares fluorescence imaging with frozen section (that do not reliably detect close margins), the added value of fluorescence imaging could actually be over-estimated. On the other hand, I appreciate the reasoning of the authors and agree on arguments posed to conclude for a potential underestimation. That's why I believe the sentence risks being simplistic, there are several factors to be considered.

We appreciate the reviewer's thoughtful comments and agree that the added value of fluorescence imaging is indeed a nuanced issue that depends on several factors, including how outcomes are measured.

We respectfully disagree with the suggestion that our method of comparison cannot reliably identify cases of inadequate margins. As discussed earlier, we use specimen-driven intraoperative frozen section assessment, enabling the detection of both positive and close margins[29]. This allows surgeons to identify inadequate margins during initial surgery and perform additional resection within the same procedure if needed, improving the adequate resection rate[31]. As recommended by the AJCC Cancer Staging Manual (8th edition), this method is standard practice at our institution. Specimen-driven assessment is critical for accurately assessing both positive and close margins during surgery, and thus for evaluating the performance of fluorescence imaging.

As mentioned in earlier responses (Reviewer #4, point 4), we also recognize that the definition of an inadequate margin is a topic of ongoing debate in the literature. In our study, we adopted a 5 mm threshold for defining an inadequate margin, in line with the Royal College of Pathologists and National Comprehensive Cancer Network (NCCN) guidelines[17, 18], as well as our institutional protocol. While we appreciate that this threshold may vary based on tumor subsite, pattern of invasion, and other clinical factors, we believe that this standardized cutoff is appropriate for assessing the performance of fluorescence imaging. Furthermore, the exact cutoff is less critical for the primary aim of our study, which was to determine whether fluorescence imaging could reliably detect margins narrower than this threshold.

We agree with the reviewer that the added value of FI could be influenced by different definitions of "close" margins. However, as stated earlier, we believe that our study is valuable in demonstrating that FI can reliably detect margins narrower than the defined threshold of 5 mm. As also mentioned in our Discussion (page 13-14, lines 562–573), although margin thresholds may differ across clinical settings, our results suggest that FI holds promise for enhancing intraoperative margin detection independent of the specific cutoff applied.

Given that we have already addressed the issue in our earlier responses, we believe that the current explanation is sufficient. The aim of our study was to assess whether FI could reliably identify margins narrower than the 5 mm threshold, and our results demonstrate that it does so effectively.

MINOR POINTS:

10. "Referencing the initial mucosal outline defined by the surgeon prior to imaging, the fluorescent areas never suggested inadequate margins, supporting the surgeon's accurate assessment–subsequently confirmed by histopathology" -> reading literally this sentence would imply that fluorescence imaging did not positively impact on mucosal margin delineation (i.e., only on the deep margin), is that the case?

Inadequate mucosal margins were indeed not detected because none were present. Fluorescence imaging therefore did not change the surgeon's initial assessment, but it consistently delineated the tumour in all cases, confirming the adequacy of the margins and demonstrating the technique's effectiveness.

To clarify this, we have revised the manuscript by deleting the following sentence (page 13, line 532-533):

"..., and no significant difference in recurrence rate was found between close and clear mucosal margins."

And adding the following sentence (page 8, line 325-327):

"This indicates that mucosal delineation can be achieved with well-defined boundaries and high contrast using FI, resulting in adequate identification of all mucosal margins."

11. "(Serious) adverse events were monitored continuously" -> "(Serious)" makes it potentially confusing and misunderstanding. Consider rewording ("Adverse events were monitored continuously" probably expresses what was done).

We have removed the term "(Serious)" from both the main text (line 152 and line 645) and the supplementary materials to avoid any potential confusion.

12. "Study proceedings did not meaningfully interfere with the standard of care, nor resulted in significantly prolonged surgery time" -> are there objective data to support this statement?

During the study, we evaluated the duration of in vivo fluorescence imaging and found that it did not take longer than 10 minutes (mean 5.52 minutes, range 4-10). Additionally, when the specimen was taken for intraoperative assessment, the surgeons continued with the neck dissection, ensuring that there were no delays due to ex vivo FI or fresh frozen sectioning.

To clarify this, we have revised a sentence in the Results section (page 8, line 320-322):

"Intraoperative FI did not interfere with the standard procedure, and the total incremental operation time related to in vivo FI never exceeded 10 minutes (mean 5.52 minutes, range: 4-10 min)"

13. Table 1 -> please, specify the Edition of TNM used.

We have specified the TNM edition used in the manuscript in the legend of Table 1 (page 22, line 842). The staging was based on the AJCC 8th edition (American Joint Committee on Cancer, 2017). We have added the specification of the TNM edition to Table 1.

14. Figure 3 -> lateral border tumors seem the easiest to be processed; could authors show (maybe as supplement) the appearance of a specimen from more complex resections?

We agree that it raises a valid point regarding the complexity of tumor resections. Considering the scope and focus of the current manuscript, and that adding such an image would not substantially change the interpretation of the results, we have not made changes to the main text. However, we are happy to share an illustrative image here. Below, a specimen from a floor-of-mouth tumor is shown, where the tumor is clearly delineated, demonstrating that even in more complex resections the tumor can be reliably identified.

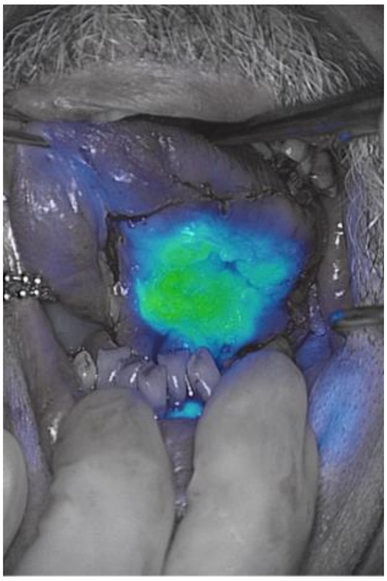

References

1. Mieog, J.S.D., et al., *Fundamentals and developments in fluorescence-guided cancer surgery.* Nat Rev Clin Oncol, 2022. **19**(1): p. 9-22.
2. Azargoshasb, S., et al., *Quantifying the Impact of Signal-to-background Ratios on Surgical Discrimination of Fluorescent Lesions.* Mol Imaging Biol, 2023. **25**(1): p. 180-189.
3. Conti, J.A., et al., *The desmoplastic reaction surrounding hepatic colorectal adenocarcinoma metastases aids tumor growth and survival via alphav integrin ligation.* Clin Cancer Res, 2008. **14**(20): p. 6405-13.
4. de Valk, K.S., et al., *First-in-Human Assessment of cRGD-ZW800-1, a Zwitterionic, Integrin-Targeted, Near-Infrared Fluorescent Peptide in Colon Carcinoma.* Clin Cancer Res, 2020. **26**(15): p. 3990-3998.
5. Smits, R.W., et al., *Resection margins in oral cancer surgery: Room for improvement.* Head Neck, 2016. **38 Suppl 1**: p. E2197-203.
6. Smits, R.W.H., et al., *Intraoperative Assessment of the Resection Specimen Facilitates Achievement of Adequate Margins in Oral Carcinoma.* Front Oncol, 2020. **10**: p. 614593.
7. Borggreven, P.A., et al., *Comorbid condition as a prognostic factor for complications in major surgery of the oral cavity and oropharynx with microvascular soft tissue reconstruction.* Head Neck, 2003. **25**(10): p. 808-15.
8. Haughey, B.H., et al., *Free flap reconstruction of the head and neck: analysis of 241 cases.* Otolaryngol Head Neck Surg, 2001. **125**(1): p. 10-7.
9. Clark, J.R., et al., *Predictors of morbidity following free flap reconstruction for cancer of the head and neck.* Head Neck, 2007. **29**(12): p. 1090-101.
10. Humphries, J.D., A. Byron, and M.J. Humphries, *Integrin ligands at a glance.* J Cell Sci, 2006. **119**(Pt 19): p. 3901-3.
11. Schittenhelm, J., et al., *Comparing the expression of integrins alphavbeta3, alphavbeta5, alphavbeta6, alphavbeta8, fibronectin and fibrinogen in human brain metastases and their corresponding primary tumors.* Int J Clin Exp Pathol, 2013. **6**(12): p. 2719-32.
12. Desgrosellier, J.S. and D.A. Cheresh, *Integrins in cancer: biological implications and therapeutic opportunities.* Nat Rev Cancer, 2010. **10**(1): p. 9-22.
13. Handgraaf, H.J.M., et al., *Real-time near-infrared fluorescence imaging using cRGD-ZW800-1 for intraoperative visualization of multiple cancer types.* Oncotarget, 2017. **8**(13): p. 21054-21066.
14. Sibinga Mulder, B.G., et al., *A dual-labeled cRGD-based PET/optical tracer for pre-operative staging and intraoperative treatment of colorectal cancer.* Am J Nucl Med Mol Imaging, 2018. **8**(5): p. 282-291.
15. Kohler, H.F., et al., *The impact of worst pattern of invasion on the extension of surgical margins in oral squamous cell carcinoma.* Head Neck, 2022. **44**(3): p. 691-697.
16. Tasche, K.K., et al., *Definition of "Close Margin" in Oral Cancer Surgery and Association of Margin Distance With Local Recurrence Rate.* JAMA Otolaryngol Head Neck Surg, 2017. **143**(12): p. 1166-1172.
17. Pathologists, T.R.C.o., *Tissue pathways for oral and head and neck pathology (G077)*. 2023: London.
18. Network, N.C.C., *NCCN Clinical Practice Guidelines in Oncology: Head & Neck Cancers, Version 5.2025*. 2025: Plymouth Meeting (PA).

19. Gokavarapu, S., et al., *Revision of margins under frozen section in oral cancer: a retrospective study of involved margins in pT1 and pT2 oral cancers.* Br J Oral Maxillofac Surg, 2015. **53**(9): p. 875-9.
20. Kerawala, C.J. and T.K. Ong, *Relocating the site of frozen sections--is there room for improvement?* Head Neck, 2001. **23**(3): p. 230-2.
21. Magliocca, K.R., *Surgical Margins: The Perspective of Pathology.* Oral Maxillofac Surg Clin North Am, 2017. **29**(3): p. 367-375.
22. Maxwell, J.H., et al., *Early Oral Tongue Squamous Cell Carcinoma: Sampling of Margins From Tumor Bed and Worse Local Control.* JAMA Otolaryngol Head Neck Surg, 2015. **141**(12): p. 1104-10.
23. Williams, M.D., *Determining Adequate Margins in Head and Neck Cancers: Practice and Continued Challenges.* Curr Oncol Rep, 2016. **18**(9): p. 54.
24. Amit, M., et al., *Improving the rate of negative margins after surgery for oral cavity squamous cell carcinoma: A prospective randomized controlled study.* Head Neck, 2016. **38 Suppl 1**: p. E1803-9.
25. Hinni, M.L., M.A. Zarka, and J.M. Hoxworth, *Margin mapping in transoral surgery for head and neck cancer.* Laryngoscope, 2013. **123**(5): p. 1190-8.
26. Kain, J.J., et al., *Surgical margins in oral cavity squamous cell carcinoma: Current practices and future directions.* Laryngoscope, 2020. **130**(1): p. 128-138.
27. Varvares, M.A., et al., *Surgical margins and primary site resection in achieving local control in oral cancer resections.* Laryngoscope, 2015. **125**(10): p. 2298-307.
28. van Lanschot, C.G.F., et al., *Relocation of inadequate resection margins in the wound bed during oral cavity oncological surgery: A feasibility study.* Head Neck, 2019. **41**(7): p. 2159-2166.
29. Aaboubout, Y., et al., *Specimen-driven intraoperative assessment of resection margins should be standard of care for oral cancer patients.* Oral Dis, 2021. **27**(1): p. 111-116.
30. de Wit, J.G., et al., *Comparison of narrow band and fluorescence molecular imaging to improve intraoperative tumour margin assessment in oral cancer surgery.* Oral Oncol, 2022. **134**: p. 106099.
31. Aaboubout, Y., et al., *Intraoperative Assessment of Resection Margins in Oral Cavity Cancer: This is the Way.* J Vis Exp, 2021(171).

Reviewer #3 (Remarks to the Author):

Although the authors attempted to address the reviewers' comments, there remains some confusion regarding the definition of diagnostic metrics and the handling of certain patients in the computation of these metrics.

1. The Statistical Analysis section still does not include a proper definition of sensitivity, specificity, PPV, and NPV with respect to what was the gold standard used for these calculations. In the response letter, the authors state that "ultimate evaluation of the efficacy (including diagnostic performance) of FI was based on the definitive histopathological assessment after surgery" however this should be clearly spelled out in the statistical section. Some details were provided in the legend of the Suppl Table 2 but these details do not clarify what the nominators and denominators in the calculations are.

We agree that the definitions of sensitivity, specificity, PPV, and NPV, as well as the gold standard used for these calculations, required clearer description in the Statistical Analysis section. We have now revised this section accordingly. Specifically, we added the following text (page 7, line 258-273):

> *"The efficacy of FI was evaluated based on its added clinical value in supporting real-time surgical decision-making, rather than as a standalone diagnostic tool. Tissue clearly distant from the tumor and judged non-suspicious by both the surgeon and pathologist (e.g., en bloc resected salivary gland tissue) was excluded from analysis to ensure a more accurate assessment of FI's clinical utility in a real-world clinical setting. Patient-level diagnostic performance for detecting inadequate resection margins was evaluated by estimating sensitivity, specificity, negative predictive value (NPV), positive predictive value (PPV), and overall accuracy; with the corresponding 95% Confidence Interval (CI) calculated using the Exact Binomial (Clopper-Pearson) method. As each patient contributed a single observation, no adjustment for intra-patient clustering was required. These metrics were derived by comparing ex vivo intraoperative fluorescence findings with definitive histopathological assessment. Fluorescent areas that corresponded to an inadequate margin were classified as true positive, whereas non-fluorescent areas that corresponded to a clear margin were classified as true negative. In addition, non-fluorescent areas that were subsequently found to have an inadequate margin were considered false negative, whereas fluorescent areas that did not show an inadequate margin on histopathology were considered false positive."*

This addition clarifies the nominators and denominators used in the diagnostic performance calculations and explicitly defines the histopathological assessment as the gold standard.

We have also expanded the Results section to provide a more detailed account of these diagnostic performance metrics, as this represents an important aspect of the study, particularly in comparison with the diagnostic performance of the current gold standard (standard IOA). We have included specific diagnostic performance metrics for the overall cohort, with additional values reported separately for close and positive margins. The following text has been included (page 10, line 381-396):

> *"This consistent detection of inadequate margins is reflected by a sensitivity of 100% (95% CI 85.0-100%). Additional metrics of diagnostic performance included a specificity of 80% (95% CI, 44.0-97.0%), a PPV of 92% (95% CI 75.0-99.0%), a NPV of 100% (95% CI 63.0-100%) and overall accuracy of 93.9% (95% CI 82.0-99.0%). The observed specificity of 80% was entirely attributable to two instances of a false-positive fluorescent signal in bone marrow. As previously described, these areas were clinically non-suspicious but fell within the resection plane and were therefore included in the analysis. These results represent a substantial improvement over standard IOA, which yielded a sensitivity of only 59.1% (95% CI 36.0-79.0%), indicating that a considerable proportion of the inadequate margins remain undetected with this conventional technique.*

Subsequently, positive and close margins were examined individually, yielding a sensitivity of 100% (95% CI 63.0-100%) and specificity of 80.0% (95% CI 44.0-97.0%) for positive margins, and a sensitivity of 100% (95% CI 78.0-100%) and specificity of 80.0% (95% CI 44.0-97.0%) for close margins. Further details on the efficacy of cRGD-ZW800-1 are provided in Supplementary Table 2."

Based on the revisions made in the Results section regarding diagnostic performance, we have also added a corresponding section in the Discussion by combining this section with an existing paragraph. This new paragraph provides a detailed comparison of our findings with the current literature and highlights the clinical relevance of our results, particularly with respect to detecting both tumor-positive and close margins. We believe this addition strengthens the discussion and situates our study clearly within the context of existing research (page 12, line 510-532):

"In this study, IOA was used as the reference standard for evaluating FI performance. Although IOA is regarded as the gold standard for intraoperative margin assessment at our institution, it is resource-intensive and routinely available only in select centres[2, 7]. Consequently, this study likely underestimates the true added value of FI, as in typical clinical practice its benefit would likely be greater, reflected by the high rate of close or positive margins after initial resection before IOA (23/31, 74%). Within this context, FI demonstrated a patient-level sensitivity of 100% for both tumor-positive and close margins, distinguishing our results from previous reports and underscoring its ability to reliably detect clinically relevant inadequate margins that might otherwise have been missed. By comparison, de Wit et al. [19] reported a patient-level sensitivity of 100% for tumor-positive margins but only 67.4% for close margins using EGFR-targeted FI with cetuximab-800CW, with a combined specificity of 41.7%. Furthermore, van Keulen et al. [39] evaluated fluorescently labelled panitumumab-IRDye800 in oral cancer, achieving 95% sensitivity and 89% specificity for detecting inadequate margins (<5 mm). Compared with these studies, our approach achieved a sensitivity of 100% and a specificity of 80%, reflecting robust detection of both positive and close margins while maintaining acceptable specificity. Although specificity varies across studies and imaging strategies, the consistently high sensitivity observed is of particular clinical relevance, as missing inadequate margins, including close margins, is strongly associated with local recurrence, the need for adjuvant therapy, and poor survival outcomes[2, 5-9]. This is especially important given that, in our cohort, the current golden standard achieved a sensitivity of only 59.1%, highlighting the substantial proportion of inadequate margins that may remain undetected with conventional techniques. Collectively, these findings underscore the potential of FI using cRGD-ZW800-1 to enhance intraoperative decision-making and improve surgical margin assessment."

Lastly, we have added the following sentence to the Abstract to highlight the clinical relevance of the abovementioned findings (Page 2, lines 51-53):

"This resulted in a patient-level sensitivity of 100% (95% CI 85.0-100%), compared with 59.1% (95% CI 36.0-79.0%) for standard intraoperative assessment."

2. Furthermore, the authors state that the calculations are at lesion-level but this is not explained anywhere and confidence intervals should be included to account for within-patient correlations.

As noted in our previous response, all analyses were performed at patient level, which is now clearly indicated in the manuscript. In addition, confidence intervals have been calculated to account for within-patient variability. This approach ensures that the reported diagnostic performance reflects clinically relevant outcomes at the patient level and provides a robust assessment of uncertainty around sensitivity, specificity, and other metrics.

3. Regarding the 4 patients for whom tissue was mistakenly taken from areas adjacent to the fluorescent spots identified by FI (point 5, R3), the authors respond that these patients were not considered false positives, but do not explain how they were counted towards the diagnostics metrics.

We would like to clarify that the four patients in whom tissue was mistakenly sampled from areas adjacent to the fluorescent spots were not counted as false positives in our diagnostic performance metrics. As specified in the Methods section, a false positive was defined as a fluorescent area corresponding to a clear margin, and a false negative as a non-fluorescent area corresponding to an inadequate margin.

In these four cases, the fluorescent signals correctly indicated the presence of inadequate margins. The apparent discrepancy arose because the fresh frozen sections were taken from adjacent tissue rather than directly from the fluorescent spots, leading to seemingly clear margins during standard IOA. Upon definitive histopathological evaluation, these margins were found to be inadequate. After reviewing these cases with the dedicated pathologist, it was confirmed that the fluorescent signal had correctly corresponded to the inadequate margins. Accordingly, these cases were classified as true positives with respect to FI, as now explicitly stated in the manuscript (page 10, line 423-429):

> *"Importantly, in four patients, the tissue samples for assessment were mistakenly taken from areas immediately adjacent to, rather than directly from, the fluorescent spots identified by FI. This sampling error resulted in seemingly clear margins during standard IOA. However, upon definitive histopathological evaluation, these margins were found to be inadequate. Review of these cases with the dedicated pathologist confirmed that the FI had correctly identified the inadequate margins. Accordingly, these cases were classified as true positives with respect to FI."*

This ensures that our reported sensitivity, specificity, PPV, and NPV accurately reflect the performance of fluorescence imaging in detecting inadequate margins.

4. Should the diagnostics metrics for standard IOA be included in Suppl. Table 2?

We have now included the diagnostic metrics for standard IOA in *Supplementary Table 2*. We agree that this is a helpful way to clearly compare the performance between the two modalities and appreciate the suggestion.

5. McNemar's test was added to the analysis but it is not listed in the Statistical Analysis section.

To address your comment, we have now explicitly included the relevant statistical tests in the Statistical Analysis section of the Methods (page 7-8, lines 276-278). The following text has been added:

> *"To assess differences in the inadequate margin detection rate between standard IOA and FI, the exact binomial McNemar's test was used. Differences in MFI were analyzed using the Wilcoxon rank test, and differences in TBR were evaluated using the Mann-Whitney U test."*

This addition clarifies which tests were applied for each type of comparison and ensures that the statistical methodology is fully transparent.

6. The statement on pg. 10, line 420 on power should be removed given that these is likely post-hoc

power calculation that is not meaningful.

We agree with your observation and have removed the statement regarding power from the specific paragraph on page 11 (line 446-457), as it represented a post-hoc calculation that is not meaningful.

7. The references in the response letter pointing to the line numbers do not match the actual line numbers in the paper.

Our sincere apologies for the inconvenience. We have carefully reviewed the manuscript and corrected the line numbers in the response letter. The references to the line numbers now accurately match the updated version of the paper.

Reviewer #3 (Remarks to the Author):

The revised manuscript attempted to address previous concerns raised after their first resubmission but managed to introduce more confusion and in fact brought to surface some serious methodological flaws with their approach. Although histopathology is stated to be the gold standard, it is only performed on tissue deemed suspicious by IOA and/or FI, biasing the estimates of sensitivity, specificity, PPV, and NPV. Fluorescence-positive but clinically "non-suspicious" tissue is selectively excluded from false positives, while missed lesions are excluded from false negatives, further inflating performance metrics. The paper mixes clinical utility arguments with diagnostic accuracy claims which are not supported given the exclusions and the decisions made in the definitions of FN, FP, TN, TP. To properly assess diagnostic accuracy, all fluorescent and non-fluorescent findings must be counted consistently against gold standard (histopathology in this case), without exclusions based on clinical relevance and this can't be evaluated in this study the way it was conducted. The paper may have clinical utility, but it requires a major revision to frame it as such and to remove the conflation with diagnostic accuracy throughout the paper.

There appears to be confusion regarding the assessment of tissue in our study. As clarified in the revised manuscript, histopathology was not limited to tissue deemed suspicious intraoperatively. All surgical specimens underwent comprehensive postoperative histopathological evaluation, including assessment of the entire resection plane, independent of intraoperative findings. Histopathology thus remains the gold standard against which all findings are ultimately evaluated, and, because the complete resection plane was assessed independently of intraoperative suspicion, the systematic omission of inadequate margins is precluded by the study design.

Nevertheless, in the context of intraoperative fluorescence imaging (FI), it is intrinsically difficult to define and quantify true negatives, and hence to report conventional diagnostic accuracy metrics such as specificity in a fully robust manner. Fluorescent foci can be classified as true positives or false positives by correlating each spot with definitive histopathology, and fluorescent-negative areas that harbor tumor on definitive histology can clearly be identified as false negatives. However, even when the entire resection plane is examined histologically, the number of discrete non-fluorescent "true negative" sites is not well defined: it remains ambiguous whether one should match each fluorescent spot with an equivalent number of negative areas, or treat large non-fluorescent surfaces as a single negative unit, and different counting schemes can dramatically alter the true negative rate and calculated specificity and accuracy. Approaches that partition the specimen into multiple planes or sides offer only a partial solution, because multiple fluorescent foci can still occur within one plane while the corresponding number of true negative sites remains arbitrary. This principle is central to our 2021 Lancet Oncology paper (Lauwerends et al.)[1], in which we state that, given these structural limitations, fluorescence-guided surgery should be evaluated primarily in terms of clinical impact and intraoperative changes in surgical management, rather than by formal diagnostic accuracy metrics that rely on an inherently ill-defined denominator of true negatives.

The current study generated a complex and highly detailed dataset, which we sought to present in what we believed to be the most clinically relevant and concrete manner. However, we

recognize that this approach could raise concerns about the methodological clarity required for diagnostic accuracy assessment. We therefore made substantial revisions to improve clarity, transparency, and interpretability, while maintaining strict scientific integrity.

We also acknowledge the reviewer's concern regarding the selective exclusion of fluorescence-positive but clinically non-suspicious tissue. Our analytic approach deliberately focused on margin-related findings that were immediately actionable during surgery, in order to highlight the real-time clinical impact and utility of FI, but we recognize that this framing may have introduced ambiguity regarding the definition of diagnostic accuracy. In light of the reviewer's suggestion, we have revised the analysis to report both perspectives in parallel: all fluorescence-positive findings, irrespective of clinical suspicion, are now included in the assessment of diagnostic performance, while margin-focused outcomes are presented separately on patient level to illustrate intraoperative utility and clinical impact. Diagnostic performance is now presented as a comprehensive evaluation in which all fluorescent and non-fluorescent findings are systematically compared to histopathology without exclusions, whereas clinical impact is described as the practical intraoperative value of FI in guiding surgical decision-making. By presenting these perspectives side by side rather than conflating them, we aim to provide a clear and methodologically robust representation of both the diagnostic properties and the clinical relevance of FI, thereby minimizing the risk of misinterpretation and aligning with established methodological standards.

Major methodological flaws

1. Sensitivity requires knowledge of all true positives that were missed. Because only suspicious margins were sent to pathology (pg. 7, line 260), undetected tumor at non-sampled margins cannot be identified, making false negatives unobservable by design.

This statement is incorrect. All tissue was sent to pathology, and all tissue was examined for the presence of malignant cells. During surgery, FI was performed first to identify any areas that might warrant additional evaluation. IOA, including fresh frozen sectioning, was then conducted independently, with both the surgeon and pathologist blinded to the FI findings. If FI indicated additional regions of interest after IOA, targeted frozen sections could subsequently be obtained. Importantly, these two processes were complementary; standard intraoperative pathological assessment was performed according to routine clinical judgement, and FI could prompt additional targeted frozen sections.

Crucially, and central to the reviewer's concern regarding sensitivity estimation, the definitive histopathological assessment was not limited to suspicious margins only. Postoperatively, all surgical specimens underwent standard pathological processing and evaluation of the entire resection plane, independent of intraoperative findings. Based on this feedback, we have clarified the Methods section to explicitly describe the comprehensive nature of the reference standard. We added the following text on page 6, lines 205–216:

> "*Following ex vivo closed-field FI, definitive histopathological assessment was performed according to standard pathology protocols, evaluating all surgical resection planes, including both deep and mucosal margins. In line with these protocols, the resection*

specimen is oriented as a six-sided cuboid, where five surfaces represent true surgical resection margins (a standard approach also used in IOA), while the original mucosal surface (the exposed tumor-bearing side) does not constitute a tissue-tissue resection interface and is therefore excluded. Mucosal margins were assessed on the surgically created lateral planes, consistent with routine practice. We applied this same systematic evaluation to FI assessment, ensuring direct comparability with pathology. This aligns with a prior report on FI in oral cancer patients[19]. All tissue blocks were subsequently examined on hematoxylin and eosin (H&E)-stained sections by a dedicated head and neck pathologist, with definitive margin status determined independently of intraoperative findings."

In addition, to avoid any remaining ambiguity regarding the basis of the diagnostic performance analysis, we added the following sentence on page 7-8, lines 287-289:

"*In the diagnostic accuracy analysis,* all *diagnostic performance metrics were derived by comparing ex vivo intraoperative fluorescence findings and standard IOA with definitive (postoperative) histopathological assessment of the entire resection plane*."

As all anatomical margins were evaluated at definitive histopathology, undetected tumor was not possible by design, and any false-negative margins would have been identifiable at final pathology.

2. Missed second primary tumors and microsatellite lesions are explicitly excluded from diagnostic performance analysis (pg. 10 400-407), even though these represent false negatives of FI. Excluding them biases sensitivity. If histopathology shows tumor and FI did not detect it, by definition this represents a false negative; see pg. 7 271-272: "In addition, non-fluorescent areas that were subsequently found to have an inadequate margin were considered false negative".

We would like to clarify that no second primary tumors were missed by FI in our study. As described in the Results section (page 11, lines 436-439), two second primary tumors were identified. One was detected using both *in vivo* and *ex vivo* FI, and the other was detected using *ex vivo* FI only. Thus, FI did not fail to detect any second primary tumor, and no false negatives occurred in this category.

We acknowledge that microsatellite lesions were not detected by FI and therefore formally represent false-negative findings from a diagnostic accuracy perspective. These lesions consisted of extremely small tumor foci (ranging from approximately 50-300 μm), collectively spanning an area of less than 0.8 mm, as described in the Results section (page 11, line 440-443). Importantly, these microsatellite lesions were located at the specimen with a clear distance (>5mm) from the resection plane and therefore did not affect margin status or intraoperative surgical decision-making. Because the primary aim of our diagnostic performance analysis was to evaluate the ability of FI to identify clinically relevant findings, these microsatellite lesions were initially excluded from this analysis.

In response to the reviewer's concern and to ensure full transparency, we have now revised our approach. Two parallel analyses are presented: the diagnostic performance analysis includes all histopathological findings, both tumor-positive and tumor-negative, assessed in relation to fluorescence regardless of their impact on margin status; and the second patient-level analysis

focuses on the detection of clinically relevant, margin-related findings. This approach allows us to provide both a strict assessment of the intrinsic diagnostic performance of FI and an evaluation of its potential clinical impact in guiding intraoperative decision-making.

In addition, the analytical approach for standard IOA has been refined. Whereas the initial analysis considered a single resection plane per patient, the revised analysis now consistently includes all five surgically created resection planes per specimen. This modification more accurately reflects routine pathological practice, as both IOA and definitive histopathological assessment systematically evaluate the specimen across these five tissue-tissue resection planes. Applying this framework ensures methodological consistency between FI and IOA and provides a more representative and clinically meaningful comparison between the two techniques. This was also part of our response to point #1, with corresponding revisions made in the Methods section (page 5, lines 187-189):

> "*During ex vivo imaging, all surgical planes were systematically evaluated based on fluorescence images acquired with the closed-field imaging system and compared to definitive histopathological assessment.*"

And (page 7-8, lines 275-315):

> *"The efficacy of FI was assessed by calculating standard diagnostic performance metrics, including sensitivity, specificity, negative predictive value (NPV), positive predictive value (PPV), and overall accuracy. Corresponding 95% confidence intervals (CI) were determined using an ANOVA-type Wilson score estimation that considers intra-patient clustering. To comprehensively evaluate both the intrinsic diagnostic performance and the clinical impact of FI, two complementary analyses were performed. First, a diagnostic accuracy analysis was conducted, in which all histopathological findings, both tumor-positive and tumor-negative, were evaluated in relation to fluorescence, irrespective of their impact on margin status. Second, a patient-level analysis was performed to evaluate findings relevant in the intraoperative surgical context. In this analysis, tissue clearly distant from the tumor and deemed non-suspicious by both surgeon and pathologist (e.g., en bloc resected salivary gland tissue) was excluded.*
>
> *In the diagnostic accuracy analysis, all diagnostic performance metrics were derived by comparing both ex vivo intraoperative fluorescence findings and standard IOA with definitive (postoperative) histopathological assessment of the entire resection plane. Fluorescent areas corresponding to either tumor localisation (including second primary lesions distant from the surgical cut) or a close margin were classified as true positives (TP), whereas non-fluorescent areas corresponding to healthy tissue (including clear margins) were classified as true negatives (TN). Furthermore, fluorescent areas corresponding to healthy tissue (including clear margins) were classified as false positives (FP), while non-fluorescent areas corresponding to either tumor localisation (including second primary lesions distant from the surgical cut) or a close margin were classified as false negatives (FN). This analysis therefore included separate tumor-positive lesions within the specimen that were not located at the surgical margin and therefore would not have impacted surgical decision-making.*

In the patient-level analysis, only margin-related findings that could potentially influence surgical management were included. This analysis focused exclusively on fluorescence findings at the surgically assessed resection planes and did not involve a combined evaluation with IOA. Fluorescent signals arising from areas clearly unrelated to the tumor margin, such as en bloc–resected salivary gland tissue outside the resection interface, were not considered relevant for intraoperative decision-making and were therefore excluded. Within this framework, fluorescent areas corresponding to inadequate (positive or close) margins were classified as TP, and a complete non-fluorescent margin surface corresponding to adequate margins classified as TN. Conversely, non-fluorescent areas corresponding to inadequate margins were considered FN, while fluorescent areas corresponding to clear margins were classified as FP. In addition, subgroup analyses were conducted, in which positive and close margins were evaluated separately. Specifically, one analysis compared positive margins with clear margins only, and one analysis compared close margins with clear margins only. In each case, fluorescence was regarded as a positive test result exclusively for the margin category under evaluation, and the same TP, TN, FP, and FN classification framework described above was applied."

Both analyses are now reported in the revised manuscript in *Table 2* and *Table 3* (formerly *Supplementary Table 2*), allowing readers to interpret the study results both for their clinical impact and under a strict diagnostic accuracy framework. Additionally, we have revised the Results section (page 10-12, lines 412-481) to more clearly present the diagnostic performance of FI relative to definitive postoperative histopathological assessment:

"The regions of highest fluorescence on the deep margin were identified and correlated with final histopathology (i.e., the sentinel margin approach[37, 38]). All close (1-5 mm) and positive (<1 mm) resection margins exhibited fluorescence intensity-peaks (example shown in Figure 3H). In the full cohort (n=31), 15 close and 8 positive margins were all successfully detected using FI, whereas nine inadequate margins were missed by standard IOA by surgeon and pathologist (three positive and six close margins). Resulting in a detection rate of 100% for FI (23/23), compared with 61% for IOA (14/23). The case in which closed-field inspection could not be performed due to malfunction of the device had a close margin (1-5 mm, n=1), which exhibited a clear fluorescence signal during postoperative FI. Focusing on the optimal-dose cohort (n=21), all 14 patients with inadequate margins (6 positive and 8 close margins) were again detected by FI, while IOA missed seven cases (three positive, four close). This corresponded to a detection rate of 100% for FI (14/14), compared with 50% for IOA (7/14). Consequently, FI demonstrated a significant higher inadequate margin detection rate compared to standard IOA for both the full cohort (100% vs. 61%, Exact binomial McNemar's test, p = 0.004) and the optimal-dose cohort (100% vs. 50%, Exact binomial McNemar's test, p = 0.008).

To assess diagnostic accuracy, all surgical resection planes were included, together with any separate tumor lesions identified within the specimen. In total, 155 surgical resection planes were assessed in 31 patients (5 resection planes for each patient). Of these resection planes, 56 exhibited fluorescence, yielding a total of 63 distinct fluorescent spots. In 47 (74.6%) of these spots, an inadequate margin was confirmed on definitive histopathological assessment. The remaining 16 (25.4%) fluorescent spots corresponded to histopathologically adequate resection margins and originated from non-tumor tissue,

specifically salivary gland tissue (n = 14) or bone marrow (n = 2). Ninety-nine resection planes were histopathologically adequate and showed no fluorescent signal. In addition, one second primary lesion (5 mm in diameter) demonstrated clear fluorescence both in vivo and ex vivo. In another case, a second primary lesion measuring 1.4 mm exhibited a distinct fluorescent signal ex vivo using closed-field flatbed imaging (Supplementary Figure 3, top row), whereas no fluorescence was detected in vivo. Furthermore, one patient presented with multiple microsatellite lesions within the specimen, at a distance of >5mm from the surgical margin, consisting of several tumor foci with diameters ranging from 50 to 300 µm (total area < 0.8 mm), none of which showed fluorescence (Supplementary Figure 3, bottom row). Overall, these findings resulted in a sensitivity of 98.0% (95% CI, 90.2-99.6%) and NPV of 99.0% (95% CI 94.5-99.9%) for FI. Additional diagnostic performance metrics included a specificity of 86.1% (95% CI 79.1-91.1%), a PPV of 75.4% (95% CI, 63.7-84.6%), and an overall accuracy of 89.7% (95% CI 84.9-94.5%). These results demonstrate robust performance of FI, even when including areas that would not have impacted surgical decision-making. The reduced specificity was largely attributable to fluorescence originating from salivary gland tissue (n=14), which can be readily distinguished from tumor tissue during surgery.

For standard IOA, each surgical specimen was systematically evaluated across the same five tissue-tissue resection planes, yielding a total of 155 assessed planes. As noted above, definitive histopathology confirmed that 47 of these margins were inadequate. IOA correctly identified 34 of these inadequate planes (72.3%), while 13 (27.7%) were missed. The remaining 108 planes were histopathologically adequate and were all classified as adequate during IOA. Notably, standard IOA detected the previously described larger second primary tumor (5.0 mm in diameter) but failed to identify both the microsatellite lesions and the small second primary tumor (1.4 mm in diameter). Consequently, IOA demonstrated a sensitivity of 70.0% (95% CI 57.2-80.5%), while maintaining a specificity of 100% (95% CI, 96.6-100%). This performance reflects a substantially higher false-negative rate compared with FI, highlighting that, despite similar overall accuracy, IOA misses a significantly greater number of tumor foci (Exact binomial McNemar's test, $p < 0.001$). Importantly, minimizing false negatives is clinically more critical than minimizing false positives, as undetected tumor tissue poses a direct risk to patient outcomes, whereas false-positive findings primarily result in additional tissue sampling without compromising patient safety. Further details on the diagnostic performance of both IOA and FI are provided in Table 2.

When evaluating the potential clinical impact of the FI performance, we found that all inadequate margins were consistently detected, yielding a patient-level sensitivity of 100% (95% CI 85.7–100%) and a NPV of 100% (95% CI 61.6–100%). In addition, the specificity was 75.0% (95% CI 40.9–93.6%), the PPV was 92.0% (95% CI 75.2–98.3%), and an overall accuracy was 93.5% (95% CI 80.5-98.5%). The observed specificity of 75.0% was entirely attributable to two instances of a false-positive fluorescent signal in bone marrow, which were clinically non-suspicious but fell within the resection plane of the primary tumor and were therefore included in the analysis. In the subgroup analysis, which assessed the detection of both positive and of close margins separately, FI achieved a sensitivity of 100% and specificity of 75.0% in both analyses. When compared with standard IOA, overall accuracy for FI and IOA was broadly similar; however, patient-level sensitivity was markedly higher for FI compared to IOA (100% vs. 70.0%, respectively), reflecting that, in our study

population, FI prevented false-negative findings altogether. Further details on the clinical evaluation of FI are provided in Table 3.

Lastly, we have added a paragraph to the Discussion section to further clarify the rationale behind the method chosen and its relevance to clinical utility (page 14-15, line 589-642):

"To comprehensively evaluate the performance of FI, we conducted two complementary analyses within this study. First, a diagnostic accuracy analysis evaluated all histopathological findings, both tumor-positive and tumor-negative, in relation to fluorescence, irrespective of their impact on margin status. Second, a patient-level analysis focused exclusively on clinically relevant margins to explore the potential implications of fluorescence for intraoperative decision-making. While specificity was higher in the diagnostic accuracy analysis compared with the patient-level analysis (86.1 vs. 75.0%, respectively), sensitivity remained consistently high in both analyses (98.0% vs. 100%, respectively). The lower specificity in the patient-level analysis resulted from the more stringent definition of true negatives, as only patients with completely adequate margins across all five surgically assessed resection planes were classified as true negatives, whereas any inadequacy in a single plane excluded the case from the true negative count. Within the study population, only one tumor focus was not detected (microsatellite lesions, total diameter < 0.8 mm); however, this focus had no clinical impact on surgical management, as it was located at the specimen at a distance of >5mm from the resection plane and therefore did not affect margin status. In comparison, IOA demonstrated substantially lower sensitivity (70%, 95% CI 57.2-80.5%). When comparing the diagnostic accuracy and patient-level analyses, the patient-level approach is particularly informative for surgical practice, as it captures the added value of FI in detecting clinically relevant inadequate margins and guiding intraoperative decisions. The consistently high patient-level sensitivity of FI is clinically significant, since missing inadequate margins is closely linked to local recurrence, the need for adjuvant therapy, and poorer survival outcomes[2,5-9]. Despite comparable overall accuracy between FI and IOA (93.5% vs. 90.5%, respectively), FI demonstrated markedly higher patient-level sensitivity (100% vs. 70.0%), underscoring its ability to substantially reduce false negatives and enhance detection of inadequate margins.

Building on these findings, it is noteworthy that, in addition to the high patient-level sensitivity for detecting all clinically relevant inadequate margins, FI also achieved 100% sensitivity in detecting both close and positive margins separately. These findings underscore the ability of FI to reliably detect clinically relevant inadequate margins that might otherwise have been missed. Although IOA is regarded as the gold standard for intraoperative margin assessment at our institution, it is resource-intensive and routinely available only in selected centres[2,7]. Consequently, the present comparison may underestimate the true added value of FI, as in typical clinical practice its benefit would likely be even greater, reflected by the high rate of close or positive margins after initial resection prior to IOA (23/31, 74%). Collectively, our findings highlight a distinct performance profile compared to earlier reports. For instance, de Wit et al.[19], reported a patient-level sensitivity of 100% for tumor-positive margins but only 67.4% for close margins using EGFR-targeted FI with cetuximab-800CW, with a combined specificity of 41.7%. Furthermore, van Keulen et al.[40] evaluated fluorescently labelled panitumumab-IRDye800 in oral cancer, achieving 95% sensitivity and 89% specificity for detecting inadequate margins (<5 mm).

Compared with these studies, our approach achieved a sensitivity of 100% and a specificity of 75%, reflecting robust detection of both positive and close margins while maintaining acceptable specificity.

The observed variability in specificity, both within the present study and relative to previously reported values, is partly attributable to the inherent challenges of defining and enumerating true negative findings in fluorescence-guided surgery. In this study, we defined true negatives as non-fluorescent surgical resection planes histopathologically confirmed as adequate, providing a practical yet approximate framework. Alternative specimen partitioning or negative area counting could alter these metrics, underscoring their limitations. Consistent with prior literature, this emphasizes interpreting FI by its clinical impact and intraoperative guidance value, rather than formal diagnostic accuracy reliant on an ill-defined denominator[10]. Still, our results show it reliably identified all clinically relevant inadequate margins, achieving 100% sensitivity and 75.0% specificity in the patient-level analysis, thereby accurately highlighting critical margins for surgical decision-making."

3. The authors exclude fluorescence-positive tissue that was deemed clinically non-suspicious from false positives (pg. 9 351-354, pg. 10 384-387), despite histology confirming absence of tumor. This directly violates the definition of specificity and inflates NPV. Only two bone marrow cases are counted as false positives, while fluorescence in other non-suspicious structures (e.g., lingual salivary gland tissue) is explicitly not counted as false positive. There is no methodological justification for this selective exclusion.

As noted in point #2, our primary analysis was intentionally restricted to clinically relevant, margin-related findings, as these are most important for guiding intraoperative surgical decisions. Fluorescence in tissue readily distinguishable from tumor (e.g., salivary gland) was therefore not counted as a false positive, reflecting its minimal impact on surgical management. We acknowledge that this approach may be confusing when evaluating strict diagnostic accuracy. As outlined above, we now complement the patient-level analysis with a dedicated diagnostic accuracy assessment, in which all histopathological findings, both tumor-positive and tumor-negative, were evaluated in relation to fluorescence, regardless of their influence on margin status. This ensures that diagnostic performance is calculated according to standard definitions. Presenting both analyses allows a comprehensive view of FI's clinical utility and intrinsic diagnostic performance, and the Results, including *Table 2* and *Table 3* (formerly *Supplementary Table 2*), have been updated accordingly.

4. There were 4 patients for whom tissue was sampled adjacent to the fluorescent signal rather than at the signal itself who had clear IOA but for whom histopathology showed inadequate margins. Classifying these cases as "true positives for FI" is a major flaw. Assuming that FI was correct just because the final margin was inadequate elsewhere assumes spatial concordance that was not histologically verified. The gold standard should be applied at the same site as the index test. If adjacency in itself is accepted as confirmation of tumor, then how are we to know that FI didn't miss tumor elsewhere (because that adjacent tissue was by the fluorescent spots but was not fluorescent itself),

and that these cases are not in fact false negatives rather than true positives.

We fully agree with the reviewer that any missed inadequate margins should be classified as false negatives rather than true positives. As explained above, in our analysis, we did not assume that FI had detected the inadequate margins. To ensure rigorous assessment, each of these cases was carefully reviewed in collaboration with the head and neck pathologist. Histological slides were examined in detail, and the spatial location of the inadequate margins was precisely correlated with the fluorescence signal observed during surgery.

This thorough evaluation confirmed that, in all four cases in question, the fluorescent signal directly coincided with the actual site of the inadequate margin, thereby providing true positive confirmation for FI. Importantly, this process ruled out the possibility of FI having missed tumor elsewhere, as the correspondence between the fluorescence signal and the inadequate margin was verified on a per-section basis.

We hope this detailed explanation demonstrates that the classification of these cases as true positives reflects accurate spatial concordance and is consistent with the intended methodological rigor.

In response to the reviewers' concern, we have revised the Results section (page 12, lines 498-506) to more clearly describe the classification of these cases:

> *"Importantly, in four patients, the tissue samples for assessment were mistakenly taken from areas immediately adjacent to, rather than directly from, the fluorescent spots identified by FI. This sampling error resulted in seemingly clear margins during standard IOA. To ensure a thorough and accurate evaluation, these cases were carefully reviewed in collaboration with the dedicated head and neck pathologist. Each corresponding histological section was examined individually, and the spatial relationship between the fluorescent signal and the inadequate margin was precisely mapped. This detailed review confirmed that the fluorescence signal had accurately indicated the location of the inadequate margin in all four cases. Accordingly, these cases were classified as true positives with respect to FI."*

Furthermore, we have added a section to the Discussion to clarify that the four cases in question were not missed due to limitations of FI (page 17, line 727-731):

> *"In four cases, accurately targeting fluorescent signals for intraoperative frozen section sampling proved challenging, limiting immediate resection. Importantly, this did not reflect failure of FI itself, as these cases were carefully reviewed in collaboration with the head and neck pathologist, confirming that the fluorescent signal accurately corresponded to the inadequate margins in all cases."*

5. The authors claim diagnostic accuracy was assessed at the patient level, but Supplementary Table 2 contains 33 TP/TN/FP/FN classifications for 31 patients, indicating that multiple observations per patient were included. This directly contradicts the authors' response raising doubts on the quality of this revision.

We acknowledge that there was an oversight in the final revision of *Table 3* (formerly *Supplementary Table 2)*. Specifically, when bone marrow cases were added as false positives, we inadvertently did not remove these two cases from the true negative category. This resulted in the table showing 33 classifications for 31 patients. We sincerely apologize for this error.

Importantly, we would like to emphasize that this clerical oversight does not reflect any underlying issue with the quality of the revisions or the analyses themselves. The correction has been made, and the table now accurately reflects each category.

6. The revision now includes sensitivity and specificity separately for "positive" and "close" margins, despite margin status being a three-category outcome (clear / close / positive). Sensitivity and specificity are defined for binary variables and the paper does not comment on collapsing categories which would result in combinations that are not meaningful, such as clear and positive tissues called as non-disease.

In the main text, we report both the overall analysis of adequate versus inadequate margins (combining close and positive margins) and a separate evaluation of close and positive margins individually. This approach was implemented for several reasons. First, positive margins are particularly prognostic and carry the greatest clinical significance, making their accurate identification especially critical for guiding intraoperative decision-making. Second, the optimal margin width (e.g., 1-5 mm) remains a topic of ongoing debate across oral cavity subsites [2-5] and evaluating close and positive margins separately allows for a transparent and clinically meaningful assessment of FI performance.

To provide this clinically relevant information, sensitivity and specificity were calculated separately for close versus clear margins and for positive versus clear margins, with each comparison treated as a binary outcome. Importantly, categories were not combined (e.g., clear and positive), ensuring that all results remain interpretable and clinically meaningful. This approach is consistent with previous work in the field. For example, de Wit et al. [6], in a comparable study published in Nature Communications, employed a similar binary comparison framework to differentiate margin status. By reporting performance for close vs. clear and positive vs. clear margins separately, we provide a nuanced and clinically relevant view of diagnostic accuracy that is aligned with both the literature and real-world surgical decision-making.

We acknowledge that the manuscript did not clearly explain that close and positive margins were analyzed separately against clear margins, and that these categories were not combined. To avoid any ambiguity, we have revised the Methods section to more clearly describe the rationale and implementation of these analyses (page 8, line 310-315):

> *"In addition, subgroup analyses were conducted, in which positive and close margins were evaluated separately. Specifically, one analysis compared positive margins with clear margins only, and one analysis compared close margins with clear margins only. In each case, fluorescence was regarded as a positive test result exclusively for the margin category under evaluation, and the same TP, TN, FP, and FN classification framework described above was applied."*

In addition, we have revised the Results section to explicitly state that close and positive margins were each analyzed in separate binary comparisons against clear margins, without combining margin categories. This clarification has been added to *Table 3* (page 30) and the Results section (page 12, lines 475-477):

> *" In the subgroup analysis, which assessed the detection of both positive and of close margins separately, FI achieved a sensitivity of 100% and specificity of 75.0% in both analyses."*

7. On one hand the authors claim that FI is "evaluated based on its added clinical value in supporting real-time surgical decision-making, rather than as a standalone diagnostic tool" yet in other places FI is presented as a standalone diagnostic tool (even the tables should refer to IOA+FI rather than FI alone): "Had FI alone guided resection decisions" (pg. 11 430).

Here, the reviewer's comment again indicates some confusion between the evaluation of diagnostic accuracy and the clinical use of FI for intraoperative decision-making. To address this, we have now deliberately presented two separate analyses and corresponding tables: one assessing the intrinsic diagnostic performance of FI, and a separate patient-level analysis reflecting its application in real-world surgical practice.

Our study protocol was specifically designed to ensure that additional resections indicated by FI could only be performed when inadequate margins were confirmed by frozen section analysis, regardless of the intraoperative assessment (IOA) findings. This criterion was independent of IOA, as the latter had missed these lesions altogether. In four such cases, FI correctly highlighted regions with inadequate margins, but the corresponding frozen section, taken adjacent to the fluorescent area, was falsely negative on histopathology. Consequently, additional resections were not permitted, although fluorescence clearly suggested that they would have been warranted.

Thus, the statement "had FI alone guided resection decisions" refers to a hypothetical scenario used solely to quantify the potential contribution of FI to surgical management. It does not imply that FI functions as a standalone decision-making tool. In practice, all fluorescence findings were interpreted in conjunction with surgical expertise and confirmatory pathology, consistent with the intended role of FI as an adjunct to intraoperative judgment, not as a replacement.

To clarify this, we adjusted the mentioned sentence as follows in the Results section (page 12, line 507-510):

> *"If fluorescence findings had been acted upon without the mandatory frozen section confirmation required by the study protocol, the inadequate margins would have been identified and addressed intraoperatively."*

References

1. Lauwerends LJ, et al. Real-time fluorescence imaging in intraoperative decision making for cancer surgery. *Lancet Oncol*. 2021;22(5):e186-e195.
2. Singh A, et al. Optimum surgical margins in squamous cell carcinoma of the oral tongue: Is the current definition adequate? *Oral Oncol.* 2020;111:104938.
3. Lin MC, et al. Adequate surgical margins for oral cancer: A Taiwan cancer registry national database analysis. *Oral Oncol.* 2021 Aug;119:105358
4. Brinkman D, et al. Impact of 3 mm margin on risk of recurrence and survival in oral cancer. *Oral Oncol.* 2020;110:104883
5. Dik EA, et al. Resection of early oral squamous cell carcinoma with positive or close margins: relevance of adjuvant treatment in relation to local recurrence: margins of 3 mm as safe as 5 mm. *Oral Oncol.* 2014;50(6):611-615.
6. de Wit JG, et al. EGFR-targeted fluorescence molecular imaging for intraoperative margin assessment in oral cancer patients: a phase II trial. Nat Commun. 2023;14:4952.

Reviewer #3 (Remarks to the Author):

The revision now is much improved and clearly separates the decision accuracy analysis vs patient-level clinical impact analysis making the approach methodologically sound.

Thank you for recognizing the methodological clarity achieved by separating the diagnostic accuracy analysis from the patient-level clinical impact analysis. We agree that this distinction strengthens the rigor of our study and underscores the complementary roles of fluorescence imaging and standard intraoperative assessment in guiding surgical decision-making.

Minor comments:

The presentation of the Results section could benefit of restructuring (possibly with separate subheadings) to clearly separate between the two analyses as now the structure is a bit dense.

We have added subheadings to clearly separate the two analyses. We agree that this restructuring enhances readability and allows the key findings of each analysis to be more easily appreciated.

There appears to be a typo: on pg. 10 line 415, 15 close margins and 8 positive margins (also listed in Table 3) are noted however pg. 13 line 547 refers to a single positive margin.

We want to clarify that the apparent discrepancy is not a typographical error. This difference reflects the timing of assessment: during intraoperative evaluation, eight positive margins were identified, whereas after additional resections and definitive histopathological assessment, only a single positive margin remained. This emphasizes the dynamic nature of margin assessment in clinical practice and highlights the added value of fluorescence guidance for real-time intraoperative decision-making.

Include the specifics of the software that was used for the analyses.

We have now included details of the software used for our analyses in the Methods section to improve transparency and reproducibility (page 8, lines 336–337):

> *"Adobe Photoshop (version 26.8.1) and R Studio (version 4.3.2) were used for statistical analysis and graph design."*
